# Effect of play-based family-centered psychomotor/psychosocial stimulation on the development of severely acutely malnourished children under six in a low-income setting: a randomized controlled trial

**DOI:** 10.1186/s12887-019-1696-z

**Published:** 2019-09-14

**Authors:** Teklu Gemechu Abessa, Berhanu Nigussie Worku, Mekitie Wondafrash, Tsinuel Girma, Johan Valy, Johan Lemmens, Liesbeth Bruckers, Patrick Kolsteren, Marita Granitzer

**Affiliations:** 1 Department of Special Needs and Inclusive Education, College of Behavioral Sciences and Education, Jimma University, Jimma, Ethiopia; 2 REVAL Rehabilitation Research Center, Biomedical Research Institute, Faculty of Rehabilitation Sciences and Physiotherapy, Hasselt University, Hasselt, Belgium; 3 Department of Psychology, College of Behavioral Sciences and Education, Jimma University, Jimma, Ethiopia; 4 Department of Population and Family Health, Jimma University, Jimma, Ethiopia; 5 Department of Pediatrics and Child Health, Jimma University, Jimma, Ethiopia; 6 Department of Healthcare, PXL University College, Hasselt, Belgium; 7 I-Biostat, Hasselt University, Hasselt, Belgium; 8 Department of Food Safety and Food Quality, University of Gent, Ghent, Belgium

## Abstract

**Background:**

The World Health Organization (WHO) recommends incorporating psychosocial stimulation into the management of severe acute malnutrition (SAM). However, there is little evidence about the effectiveness of these interventions for SAM children, particularly when serious food shortages and lack of a balanced diet prevail. The objective of this study was to examine whether family-based psychomotor/psychosocial stimulation in a low-income setting improves the development, linear growth, and nutritional outcomes in children with SAM.

**Method:**

Children with SAM (*N* = 339) admitted for treatment to the Jimma University Specialized Hospital, Ethiopia, were randomized to a control (*n* = 170) or intervention (*n* = 169) group. Both groups received routine medical care and nutritional treatment at the hospital. The intervention group additionally received play-based psychomotor/psychosocial stimulation during their hospital stay, and at home for 6 months after being discharged from hospital.

The fine motor (FM) and gross motor (GM) functions, language (LA) and personal-social (PS) skills of the children were assessed using adapted Denver II, the social-emotional (SE) behavior was assessed using adapted Ages and Stages Questionnaires: Social-Emotional, and the linear growth and nutritional status were determined through anthropometric assessments. All outcomes were assessed before the intervention, upon discharge from hospital, and 6 months after discharge (as end-line). The overtime changes of these outcomes measured in both groups were compared using Generalized Estimating Equations.

**Results:**

The intervention group improved significantly on GM during hospital follow-up by 0.88 points (*p* < 0.001, effect size = 0.26 SD), and on FM functions during the home follow-up by 1.09 points (*p* = 0.001, effect size = 0.22 SD). Both young and older children benefited similarly from the treatment. The intervention did not contribute significantly to linear growth and nutritional outcomes.

**Conclusion:**

Psychomotor/psychosocial stimulation of SAM children enhances improvement in gross motor functions when combined with standard nutrient-rich diets, but it can enhance the fine motor functions even when such standard dietary care is not available.

**Trial registration:**

The trial was retrospectively registered on 30 January 2017 at the US National Institute of Health (ClinicalTrials.gov) # NCT03036176.

**Electronic supplementary material:**

The online version of this article (10.1186/s12887-019-1696-z) contains supplementary material, which is available to authorized users.

## Background

Malnutrition is one of the global challenges to children’s health. In 2015, worldwide 156 million children under five were stunted, 50 million were wasted, and 42 million overweight [1]. In low-income countries in Africa and Asia, the prevalence of malnutrition is higher. In Ethiopia for example, it is estimated that 38% of children under five are stunted, 24% are underweight and 10% wasted [2]. For many children, the problem already starts during intrauterine life. Poor nutrition during intrauterine life and the child’s early years leads to profound and varied effects such as delayed physical growth, impaired motor and cognitive development resulting in lower IQ, more behavioral problems and deficient social skills at school age, decreased attention, deficient learning, and lower educational achievement [3–7]. Such negative consequences, however, can be ameliorated through appropriate interventions.

Providing adequate nutrition, early psychosocial stimulation at home, appropriate preschool experiences, and learning opportunities could substantially increase cognitive development of children [8] and contribute to longer-term gains in human capital [9]. Reviews of studies on nutrition and stimulation for malnourished children in general [10, 11] and for the severely acutely malnourished ones in particular [12] has shown that supplementing dietary rehabilitation with psychosocial stimulation can potentially reduce the adverse effects of undernutrition and improve developmental outcomes. The World Health Organization (WHO) already recommends using psychomotor/psychosocial stimulation for children in severe food shortage situations [13] and those receiving treatment for severe acute malnutrition (SAM) [13–16]. The recommendation has a dual objective: to help recover the psychomotor/psychosocial deficit, and stimulate the SAM children to regain their appetite more quickly and gain weight faster. It assumes that integrating the two treatments would have synergistic effects [17, 18]. However, the evidence for this comes mainly from two studies; one uses a non-randomized design with mixed outcomes.

Though the WHO recommends clinical discharge with shorter hospital admission periods followed by home-based care [15], providing strict dietary rehabilitation at some home settings is also hardly possible, especially in remote and inaccessible rural areas. As most SAM children come from poor families, they return to the same poor home situation. Though ready-to-use-therapeutic food (RUTF) can be used effectively at home, children living in remote rural areas far from health centers rarely get adequate supplies of RUTF. Thus, little is known about how much psychomotor/psychosocial stimulation benefits SAM children living in settings where not even a basic diet for survival is ensured, let alone essential dietary nutrients. Moreover, since many stimulation studies have so far focused on children less than 24 months of age, much is unknown with regard to older children. Above all, the evidence supporting the recommendation of psychosocial stimulation for children with SAM is inadequate, and has been criticized for being low in quality across important outcomes [12]. Therefore, further studies are needed in different low-income settings in order to identify the best strategies to support parents in caring for their young children [19]. This study was aimed at examining the effect of play-based stimulation on the development, linear growth, and nutritional outcomes during hospital and home-based treatment of SAM children under 6 years of age in the low-income context of Jimma Zone, South West Ethiopia. The primary outcomes were developmental performances in the form of *fine motor* (FM) functions (such as picking things up between finger and thumb, or grasping and drawing) and *gross motor* (GM) functions (such as using arms, legs, feet, or entire body for crawling, running, and jumping), *language* (LA) and *personal-social* (PS) skills (such as smiling, self-feeding, helping, and playing with others) and *social-emotional behaviors* (SE) (such as autonomy, adaptive functioning, affect, compliance, communications, interaction with people, and self-regulation) The secondary outcomes were height/length-for-age z-score [HAZ], mid-upper-arm circumference z-score (MUACZ), weight-for-age z-score (WAZ), weight-for-height z-score (WHZ) or body-mass index-for-age z-score (BAZ) at discharge from hospital and after 6 months of follow-up at home.

## Method

### Study design and subjects

A longitudinal intervention study was conducted on SAM children admitted to the nutrition rehabilitation unit (NRU) of the Jimma University Specialized Referral Teaching Hospital, South West Ethiopia. With a randomized, single blind (data collectors not knowing the treatment group of participants), parallel group trial design, eligible participants were assigned to a control or an intervention group. Data collection occurred between 8th February 2011 and 19th November 2013. The study was held up due to a delay in the adaptation process of the tools used for data collection, and it took longer than planned to enroll an adequate number of eligible participants within accessible distances for follow-up. Admission to and discharge from the NRU were based on the WHO guidelines adapted by the Ethiopian Ministry of Health for the treatment of SAM children [20]. In a small number of cases, however, patients were discharged earlier in order to free up treatment space for new, more severe patients. SAM children between 6 and 60 months of age who fulfilled the following criteria were included: weight for height or weight for length less than 70% of the median on National Centre for Health Statistics [21] of USA; or mid upper arm circumference (MUAC) < 110 mm with a length > 65 cm; or, having bilateral pitting edema and having no medical complications (at Transition Phase i.e., Second phase). There are three phases (Phase I, Transition Phase and Phase II) of treatment of the SAM children, and the details are available in the protocol prepared by the Ethiopian Ministry of Health [20]. SAM children who were completely deaf or blind, who had complications hindering mobility for play, whose primary caregiver could not provide stimulation due to physical or mental disability were excluded from the study. Only one child was randomly selected from a family with more SAM children. Children from inaccessible areas and far distances (more than a 50 km radius of Jimma Town) were also excluded.

#### Sample size

The study was intended to detect a 5% difference in developmental performance ratio score between the control and the intervention groups after 6 months of follow-up. The performance ratio is the ratio of the number of test items a child performed successfully to the number of items he/she was expected to perform for his/her age. These numbers were determined in line with the test item administration and scoring guidelines presented in Denver II [22, 23]. A power of 80% was specified at a 5% significance level, assuming a 20% loss to follow-up. Estimates of the variance in developmental performance ratio scores, to be used in the power calculations, were obtained from cross-sectional data of 22 non-malnourished, healthy Ethiopian children living in the study area (36–69 months of age; mean ± SD =51.4 ± 8.2) [Snijers, Inne: Objectivity, stability and feasibility of the Denver II-Jimma: an exploratory pilot study, unpublished MA Thesis, Unpublished]. Of the four outcomes assessed using Denver II, the mean score of language (1.05 ± 0.14) was used since it was the developmental outcome with the highest variance. The data from these children were used simply because we had no other child development data collected with a culturally adapted tool to be used in the present study. Accordingly, a sample of 136 SAM children in each group was expected to sufficiently power the study. We conducted an interim analysis which showed a larger variance in developmental performance scores for SAM children than for non-malnourished healthy children. Hence, we recruited 25% more children to each arm of the study to increase the sample size.

#### Randomization and blinding

Eligible children were randomized using computer-generated codes and allocated to the control (*n* = 170) and intervention (*n* = 169) groups. This was done every week by the researcher coordinating the study. Allocation concealment was ensured as the researcher had no physical access to the children.

Testers, who did not know whether a child belonged to the control or the intervention group, assessed the children in a separate room; intervention nurses worked in a separate play room which was accessible only for the intervention children and their caregivers (parents, grandparents and siblings).

### The intervention

#### *Play facility and intervention nurses*

Prior to the start of the intervention, an appropriate infrastructure for psychomotor and psychosocial play activities was set up at the pediatric ward of Jimma University’s Specialized Referral Teaching Hospital. A playroom and a playground were installed and furnished with basic facilities for engaging the SAM children in play-based motor, language, and personal-social activities. Three female clinical nurses, who were not members of the hospital staff, were trained as intervention nurses to stimulate the SAM children directly and also to transfer skills to caregivers on how to stimulate the SAM child through play activities. The intervention nurses who were familiar with the local cultures, and could speak the two major languages used in the area received one week of training in the theory of child development, and one month of intensive practice (4 hr. daily) in implementing developmental stimulations. A play therapist, an occupational and a physiotherapist, special educator and a psychologist in consultation with a neuroscientist and nutritionists prepared the training package (see Additional file 1 to get highlight of the package).

#### *Stimulation phases and activities*

The intervention was offered in two phases: in-patient (during the Transition Phase) and out-patient (during Phase II). The first, or in-patient phase was provided in the hospital between the Transition Phase and the discharge from the hospital. Two types of sessions were offered: individual sessions in the playroom and group sessions on the playground. A minimum of 8–10 play sessions lasting for about 20–40 min each were planned to be held in the presence of the caregiver in the playroom only, or both in the playroom and the playground, depending on the age and health status of the child. The intervention included auditory, tactile and visual stimulation, hand-eye coordination, and different types of sensory-motor training that included fine and gross motor activities. The guiding principle was enhancing a child’s holistic development—cognitive, emotional, language physical, and social—in an integrated manner by using age-appropriate play materials, cultural tools, and resources with the caregivers playing the crucial role of mediation. By attending play sessions, caregivers were trained through demonstration and active engagement on how to stimulate a child. Moreover, they received information on childcare and feeding and their importance for the development and growth of children. Simple play materials such as balls, picture cards, and animal-shaped toys were used to engage children in different age-appropriate activities that contribute to cognitive, emotional, language, physical, and social development.

The out-patient phase of the intervention occurred at home after discharge from the hospital. The standard criteria for discharge are for the SAM child to attain W/L > =85% or W/H > =85% on more than one occasion, and to have had no edema for 10 days. In addition to the play materials offered on discharge, new play material was offered during each of the three planned home visits over a 6 month follow-up. Three home visits (in the 3rd, 7th and 13th week) were made during this period to provide further stimulation to the SAM child and empower the caregivers of the SAM child. Empowerment of the caregivers included training on how to stimulate the SAM child, and further improvement of the mothers’ and other family members’ knowledge of childcare and feeding, proper nutrition, and stimulation. The family members were encouraged to show affection to the SAM child, be responsive to their cues, interact with them using the resources available at home and the simple play materials offered to them. The intervention utilized ideas from the Mediational Intervention for Sensitizing Caregivers program [24–26] and mediated learning experiences [27]. The key issue in the intervention was enhancing interactions between the caregivers and the target child through play, guided by a principle of Safety, Enjoyment and Stimulation. Different supervisors participated in home visits to ensure that the intervention nurses could work in a qualitative way with the target child (check whether the child had been using play materials given to him/her and had been taking part in interactive play) and the caregivers (providing information/feedback and education on childcare, feeding and stimulation, and demonstrating how to use the new play materials offered to child). At each home visit, the play leaders first ensured that the child was using the play materials offered to him/her in line with his/her age. Educational play materials carefully selected by child therapists were given to the children, taking in account the following three age categories: 6 months to 2 years, two to 4 years, and four to 6 years. The child and caregiver (including family members and child’s playmates in the neighborhood) were then introduced to techniques of play using the newly provided play materials, taking into account the level of the developmental status of the child. The mothers were encouraged to use additional local materials besides the commercial toys provided by the intervention nurses. Toys such as ‘African Family’ and ‘African Animals’, picture cards, cubes, acrobats and balls were provided for each child. Siblings and peers in the neighborhood were also encouraged to play with the target child. Some home visits were not conducted exactly on the scheduled date, and some visits were cancelled during the rainy season, thereby reducing the total number of home visits to less than the three planned moments. Some visits were made in the absence of the mother, but a caregiver was available at home. On the last home visit, the intervention nurses interviewed the caregivers using a structured questionnaire. This aimed to provide information on the progress of the child, the adherence of the parents to advice about offering home-based stimulation, and the major challenges they had encountered.

The control children received routine medical care and dietary treatment offered at the nutritional rehabilitation unit of the hospital. Although they had access to facilities in the playground, they had no access to the playroom and were not provided with stimulation and play materials. Both groups received standard formula diets (F-75, F-100, or ready-to-use therapeutic food) that contain vitamin A, folic acid, iron and all the other nutrients (potassium, magnesium and zinc) required to treat a malnourished child. F-75 and F100, or ready-to-use therapeutic food (RUTF) were used for in-patient care. F-75 (75 kcal or 315 kJ/100 mL) is a therapeutic milk used only during the initial phase of the treatment (Phase 1), whereas F-100 (100 kcal or 420 kJ/100 mL) is a therapeutic milk used during the rehabilitation phase (Transition Phase and Phase 2) of the treatment. Whenever patients have a good appetite and no major medical complications, they enter Phase 2, when they are given RUTF (used in both in-patient and out-patient settings) or F100 and iron (used in in-patient settings only) according to look-up tables. On discharge, some packets of RUTF are given for home intake. After returning home, the SAM child is taken by a caregiver to a nearby therapeutic feeding center or health center, where registration is conducted and the child gets a Unique SAM ID number. The SAM child is followed using an individual follow-up chart and freely given RUTF periodically.

### Outcomes and measurements

Outcomes were assessed by nurses trained for these purpose as testers. The testers were blinded of the treatment group to which the SAM children belong, and the intervention nurses had no role in testing..

#### *Developmental performance*

The primary outcomes of the study were the SAM children’s developmental scores in FM, GM, LA, PS and SE.

Performances in FM, GM, LA and PS were assessed using the Denver II-Jimma [28], a culturally adapted and standardized screening tool to assess the development of children under 6 years of age in the Jimma zone of Ethiopia. It was created by adapting 36 of the 125 test items of the Denver II child development screening test. It has an excellent inter-rater on 123 (98%) items and substantial to excellent test-retest reliability on 119 (91%) items [28]. The Denver II-Jimma comprises a total of 125 test items across the four domains. It was adapted and standardized on 1597 healthy children 4 days to 70.6 months of age. The 25, 50, 75 and 90 percentile passing ages were determined for each test item as milestones. The number of test items that a child has successfully performed (passed) is described as the performance score. Most Denver II-Jimma test items assess the performance of a child through direct observation, while a few use parental reports.

The problems in SE competences (self-regulation, adaptive functioning, affect, compliance, autonomy, interaction with people and communication behaviors) were assessed using the parent-completed Ages and Stages Questionnaire: Social-Emotional (ASQ:SE), adapted to the study context (unpublished) through a collaboration between a European child psychiatrist and two local academics: a psychologist and a special educator. The adapted items were translated into two local languages commonly used in the study area, piloted and amended before use. Through a semi-structured interview with a caregiver, the social-emotional behavior of a target child was assessed on different items. The score on each item is as follows: a score of 0 indicates a normal behavior (the absence or rarely happening of a problem behavior); a score of 5 indicates the presence of a problem behavior; 10 is a problem behavior with more frequent occurrence; 15 is a problem behavior which is a concern for the caregiver. A child’s total behavior score is obtained by adding up item-specific scores.

#### *Growth and nutritional status*

The secondary outcomes were linear growth, quantified by HAZ, and nutritional status, determined using MUACZ score, WAZ and WHZ for children below 60 months of age, and BAZ for children 60 or more months of age.

The child’s height, weight, and MUAC were measured using a stadiometer/length-mat, calibrated digital weight scale, and MUAC tape respectively, following standard procedures [29]. At each test moment, anthropometric measurements were repeated. If different values were obtained, a third measure was taken and their average was used to calculate z-scores based on WHO standards [30].

Developmental performance, growth and nutritional status were measured at the hospital before the intervention (as baseline), on discharge, and at home 6 month after discharge from hospital (end-line).

#### *Socio-demographic information*

Socio-demographic information was collected using a structured questionnaire. Caregivers were also requested to provide information on the socio-demographics of the mother and child.

#### *Follow-up information*

During each home visit, the health and dietary condition of the intervention child, and the adherence of caregivers to run the home-based stimulation sessions were documented using a structured questionnaire. Some data on factors assumed to be affecting the performances within the intervention SAM children were gathered. Information collected include caregivers’ feelings about the general health condition of the intervention SAM child, family support and engagement in the psychosocial stimulation service, the child’s access to nearby health centers after discharge from hospital, the and availability of RUTF. Caregivers were asked to give their subjective rating (always, sometimes, rarely and never at all) on how often the child was getting RUTF, and whether the family provides specially prepared food for the SAM child.

#### Assessment procedure

After informed consent was obtained from the caregivers, developmental and anthropometric assessments were made during the Transition Phase of the nutritional treatment. Caregivers were first interviewed to complete the questionnaire on socio-demographic information. Then the child’s development was assessed: first the ASQ:SE, followed by the Denver II-Jimma test. Lastly, anthropometric measurements were made in the following order: weight, MUAC, and length or height.

Five clinical test nurses were initially trained in anthropometric measurements and the administration of ASQ:SE and Denver II-Jimma test items. However, two who were not employed as hospital staff, and took part in data collection during adaptation and standardization of the Denver II-Jimma, collected 74% of the baseline, 81% of the discharge and 99% of the exit data. The testers did not know to which treatment group a child was allocated, though there was sometimes a possibility to guess during an exit testing whether a child had been visited during follow-up (which was the case only for the intervention children).

### Statistical analysis

The developmental outcomes were summarized in terms of count scores as described earlier. These scores were entered into statistical models as continuous outcomes. Anthropometric outcomes were summarized in terms of continuous z scores. Independent two samples t-test (for the count and the continuous data) and chi-square test (for categorical data) were used to compare the baseline characteristics of the: 1) control and intervention groups and, 2) children who completed the follow-up period and children lost to follow up.

For each outcome, every child (ideally) contributed three measurements: one at baseline, one at discharge, and one at the end of follow-up. A generalized estimating equations (GEE) model with an intention-to-treat approach was used to account for the longitudinal design. The working variance-covariance matrix was an unstructured matrix, except the personal-social outcome, for which an exchangeable structure was specified due to error in convergence condition.

The primary analysis aimed to: 1) examine the change in developmental outcomes during the hospital-based follow-up (from baseline to discharge) and during the home-based follow-up (discharge to end-line), and 2) test for possible group differences in these over time changes. Therefore, the statistical model included a common intercept for the control and intervention group, and fixed effects for time, group, and their interaction. The time variable in this model is a 3-level variable (i.e. baseline, discharge and end-line). No other explanatory variables were included. Effect sizes were calculated from the GEE models by transforming the intervention effects for each outcome into standardized scores. The length of stay in the hospital (in-patient phase) and the interval within the three testing moments were not controlled for by the study design. Consequently, measurements were not taken at fixed time intervals. Therefore, the basic analysis was complemented with an analysis considering a continuous timescale. The hospital-based and the home-based follow-up periods were merged into a continuous time variable containing the number of days in the study. The time variable was used in a GEE model, where a group-specific curvilinear evolution over time was allowed.

Separate analysis was conducted for each of the primary outcomes and no multiple testing correction adjustments were made. For each outcome, the model was expanded with explanatory variables: child’s sex and baseline age, baseline developmental score, and baseline WAZ or baseline MUACZ scores (for primary outcomes), and baseline anthropometric z-scores (for secondary outcomes). Variables which differed significantly between the two groups at baseline were included as covariates in models comprising the ‘group’ variable. To avoid multicollinearity between WAZ and MUACZ scores, the score with a greater correlation with the outcome variable was selected. The interactions between baseline age and time, and baseline score and time were also included simultaneously in the expanded model.

The final models included three variables potentially capable of modifying the treatment effect on the primary and the secondary outcomes. Each of these variables (sex, baseline age and baseline developmental level, or baseline anthropometric z-scores) and their interactions with the duration of follow-up and with treatment were separately examined. A backward selection procedure was used to obtain a parsimonious model. Statistical significance was set at *p* < 0.05.

For the intervention group, a GEE model was also employed to look into the effect of aspects related to the intervention on the outcomes. The frequency of daily home-based guided stimulation of the child, the social-emotional score of the child, whether the child had been sick, receiving ‘RUTF, or getting a specially prepared diet during the follow-up period were entered simultaneously in the GEE model.

Finally, the developmental performances (FM, GM, LA, PS and SE) and two anthropometric indices (WAZ and MUACZ) of the SAM children in the control and intervention groups were compared to nearly age-matched healthy children using multiple regression (with a correction for age and gender) (a) before the start of the intervention in hospital, and (b) six or more months after discharge from hospital. Data analysis was conducted using Stata (Version 12, StataCorp, College Station, Texas) [31].

### Ethics

The study was carried out in accordance with the Helsinki Declaration and international ethical guidelines for biomedical research involving human subjects. Ethical approval was obtained from the Research Ethics Review Board of Jimma University (RPGC/217/2010), Ethiopia, and Hasselt University (CME 2010/306), Belgium. Child caregivers signed informed consent to participate in the study. SAM children in hospital were provided all routine medical care, and the facilities at the playground were accessible to all, regardless of the treatment group they were assigned to. The trial was registered at the US National Institutes of Health (ClinicalTrials.gov) # NCT03036176.

## Results

### Baseline characteristics of the control and the intervention groups

**Fig. 1:**
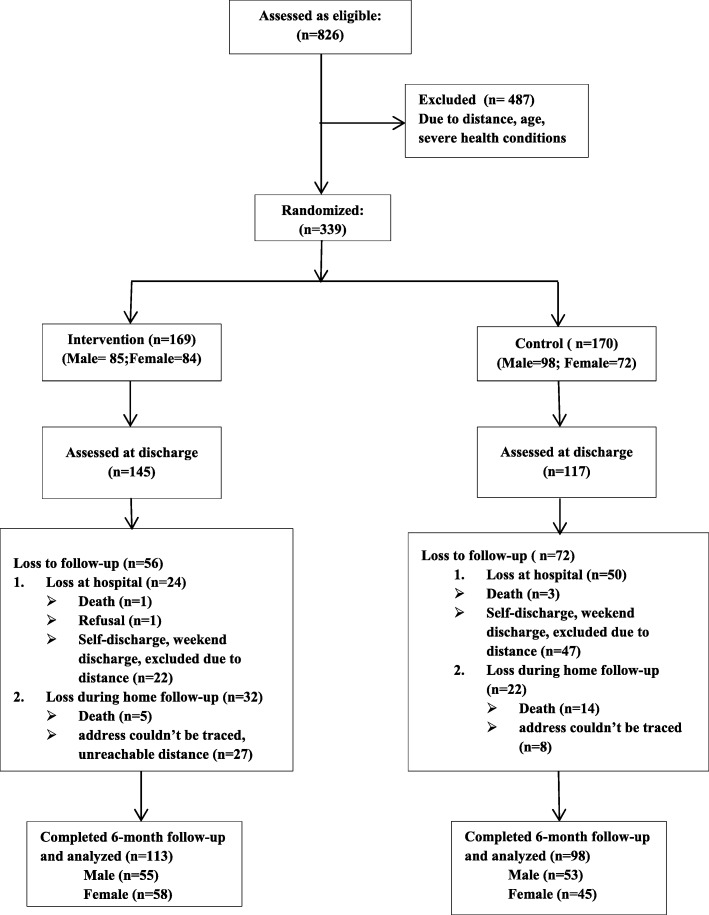
Flow chart of sample enrolled and finally analyzed

In total, 339 SAM children (male =183; mean ± SD age = 27.4 ± 15.1mo, range = 6.1—65.7mo) were enrolled in the study (control = 170 and intervention = 169) (Fig. 1). The composition of the control and intervention group was compared for baseline child, maternal, and family characteristics. The results are presented in Table 1. The two groups differ significantly in terms of living area, maternal occupation, child’s baseline WAZ and MUACZ scores. More children from urban or semi-urban areas were assigned to the intervention (30.8% versus 15.9%, *p* = 0.001). Significantly more mothers in the control group were housewives (90.4% versus 78.2%, *p* = 0.002). In terms of the MUACZ and WAZ scores, the intervention children had better baseline scores than the control children (z-score = − 3.1 versus − 3.5, *p* = 0.014; and − 3.6 versus − 3.9, *p* = 0.048 respectively). No statistically significant differences were observed between control and intervention children at baseline on child age, sex, birth order, developmental performance, family size and income. The two groups were also similar in duration of stay in hospital (mean (SD)12.12 ± 9.7, range 1–46 days for control group; 12.10 ± 8.3, range 2–48 days for intervention group), nutritional status as measured by HAZ and WHZ or BAZ, and in maternal education. Most of the children (80%) in each group belonged to illiterate mothers; more than 70% of them lived in a family of 3 to 6 persons; more than half were born after a second child; more than 98% lived in a family with a monthly income of less than 1500 birr (about 72 USD at the time); more than 78% of the mothers were younger than 31 years of age.

**Table 1:** Baseline characteristics (child, maternal and family characteristics) of SAM children by trial arm (*N* = 339), displayed as n (%) or mean [95% CI]

Child characteristics	*N* = 339	Intervention	Control	*p*-value
Female (n, %)	339	84 (49.7)	72 (42.4)	0.175
Birth order (born after second child) (n,%)	337	90 (53.3)	97 (57.7)	0.589
Age, months	339	27.5 [25.3, 29.7]	27.3 [24.9, 29.7]	0.908
HAZ baseline score	337	−3.7 [− 4.0, − 3.5]	−3.8 [−4.1, − 3.5]	0.558
MUACZ baseline score	332	−3.1 [− 3.3, − 2.9]	−3.5 [− 3.8, − 3.2]	0.014*
WAZ baseline score	338	− 3.6 [− 3.8, − 3.4]	−3.9 [− 4.1, − 3.6]	0.048*
WHZ or BAZ baseline score	337	−2.3 [− 2.5, − 2.0]	−2.6 [− 2.9, − 2.3]	0.128
Fine motor baseline score	338	15.6 [14.9, 16.3]	15.3 [14.5, 16.1]	0.580
Gross motor baseline score	339	16.2 [15.5, 17.0]	16.3 [15.4, 17.1]	0.998
Language baseline score	339	16.6 [15.7, 17.6]	17.1 [16.0, 18.2]	0.546
Personal social baseline score	339	14.2 [13.3, 15.0]	14.0 [13.0,15.0]	0.773
Social-emotional baseline score	336	67.3 [62.5, 72.0]	66.8 [62.2, 71.4]	0.888
Maternal characteristics				
Age (≤30 years) (n, %)	330	134 (81.2)	130 (78.8)	0.582
Education (illiterate) (n,%)	338	135 (79.9)	135 (79.9)	0.100
Occupation (housewife) (n, %)	331	129 (78.2)	150 (90.4)	0.002*
Family characteristics				
Family size (2–6 persons) (n, %)	339	121 (71.6)	121 (71.2)	0.932
Socio-economic status (< 2.4 USD per day) (n, %)	338	167 (98.8)	166 (98.2)	0.652
Address (rural or small village) (n, %)	339	117 (69.2)	143 (84.1)	0.001*

T-test used for comparison on continuous score summarized by Mean (SD); Chi-square test for comparison of binary scores summarized by n (%); ^*^ *p* < 0.05

### Baseline characteristics related to loss to follow-up

Of the 339 children initially enrolled, only 211 children (98 control, 113 intervention) completed the study (Fig. 1). The rate of loss to follow-up did not differ significantly between the two groups (42.4% control versus 33.1% intervention, *p* = 0.08). Reasons for loss (Fig. 1) include death of the children (*n* = 23), self-discharge, weekend discharge and/or failure to trace a child’s address or/and failure by nurses to travel long distances for home follow-up (intervention, *n* = 47; control, *n* = 55), and a refusal for follow-up by one intervention child.. The baseline characteristics of children completing the study and those lost to follow-up were compared on socio-demographic profiles, and main study outcomes at baseline (child development and growth) (Table 2).

**Table 2:** Baseline characteristics (child, maternal and family characteristics) for the SAM children completing the study and the SAM children lost to follow-up displayed is n (%) or mean [95% CI]

	N (339)	Completers (*n* = 211)	Lost to follow-up (*n* = 128)	*p*-value
Child characteristics				
Demographic characteristics				
Female (n, %)	339	105 (49.8)	51 (39.8)	0.076
Birth order (born after second child) (n,%)	337	113 (53.8)	74 (58.3)	0.258
Age, months	339	26.7 [24.7, 28.7]	28.5 [25.7, 31.3]	0.303
Linear growth and nutritional status				
HAZ baseline score	337	−3.7 [−4.0, − 3.5]	−3.8 [−4.2, − 3.5]	0.641
MUACZ baseline score	332	−3.2 [− 3.4, − 3]	− 3.5 [− 3.8, − 3.2]	0.110
WAZ baseline score	338	− 3.7 [− 3.9, − 3.5]	−3.8 [− 4.1, − 3.6]	0.420
WHZ or BAZ baseline score	337	−2.4 [− 2.7, − 2.2]	−2.5 [− 2.8, − 2.1]	0.893
Developmental status				
Fine motor baseline score	338	15.6 [14.9, 16.2]	15.3 [14.4, 16.3]	0.634
Gross motor baseline score	339	16.3 [15.6, 17]	16.2 [15.3, 17.1]	0.906
Language baseline score	339	16.4 [15.5,17.3]	17.6 [16.3, 19]	0.135
Personal social baseline score	339	14 [13.1, 14.8]	14.3 [13.2, 15.4]	0.644
Social-emotional baseline score	336	64 [59.8, 68.1]	72.1 [66.7, 77.5]	0.009^*^
Maternal characteristics				
Age (≤ 30 years) (n, %)	330	166 (79.4)	98 (81)	0.732
Education (Illiterate) (n,%)	338	203 (96.6)	123 (96.1)	0.782
Occupation (housewife) (n, %)	331	175 (84.5)	104 (83.9)	0.871
Family characteristics				
Family size (2–6 persons) (n, %)	339	158 (74.9)	84 (65.6)	0.068
Socio-economic status (< 2.4 USD per day ^a^) (n, %)	338	207 (98.6)	126 (98.4)	0.921
Address (rural or small village) (n, %)	339	154 (73)	106 (82.8)	0.038^*^

^*^ *p* < 0.05

^a^ Is about 1500 Ethiopian birr per month

At baseline, children who completed the study and those lost to follow-up did not differ significantly except on the ASQ-SE score and location of residential area (urban / rural or small village).

On average, children completing the study had fewer social-emotional problems (as indicated by the lower scores) than children lost to follow-up (64 versus 72.1, *p* = 0.009), and those living in small villages or rural areas were more likely to drop out (83% versus 73%, *p* = 0.038). The percentage of males and females among study completers is nearly the same (50%). However, the percentage of lost to follow-up is higher among males: 32.7% of the girls and 42.1% of the boys were lost to follow-up.

### Developmental and nutritional outcomes of the study completers

Both the control and the intervention groups that completed the study improved during the follow-up period. Table 3 summarizes the mean scores of the two groups at baseline, discharge and end-line measurements.

**Table 3:** Group-wise comparison of the baseline ^a^, discharge ^b^ and end-line ^c^ developmental and nutritional characteristics of study completers, displayed as mean [SD] and *p*-value of their difference (*N* = 339)

Developmental outcomes										
Time	Fine motor		Gross motor		Language		Personal-social		Social-emotional	
	Group		Group		Group		Group		Group	
	Cont.	Int.	Cont.	Int.	Cont.	Int.	Cont.	Int.	Cont.	Int.
Baseline^a^	15.3 [5.1]	15.8 [4.4]	16.1 [5.5]	16.5 [4.7]	16.2 [6.9]	16.6 [6.1]	13.5 [6.6]	14.4 [5.3]	63.9 [31.5]	64.1 [30]
*p*-value ^d^	*p* = 0.200		*p* = 0.301		*p* = 0.360		*p* = 0.141		*p* = 0.483	
Discharge^b^	15.7 [4.6]	16.8 [4.3]	16.1 [5.6]	17.7 [4.9]	16.4 [6.9]	17.3 [6.6]	13.8 [6.1]	15 [5.6]	48.6 [27.3]	50.1 [29.2]
*p*-value^d^	*p* = 0.046		*p* = 0.020		*p* = 0.177		*p* = 0.082		*p* = 0.361	
End-line^c^	17.9 [4.1]	19.3 [3.3]	20.4 [5.8]	21.1 [5.3]	20.1 [7.4]	21.0 [6.9]	16.9 [5.1]	17.7 [4.4]	50.1 [28.9]	48.3 [22.8]
*p*-value ^d^	*p* = 0.003		*p* = 0.210		*p* = 0.186		*p* = 0.110		*p* = 0.695	
Overall mean	16.3 [4.8]	17.3 [4.3]	17.6 [6.0]	18.4 [5.3]	17.7 [7.3]	18.3 [6.8]	14.8 [6.1]	15.7 [5.3]	54.5 [30.1]	54.2 [28.3]
Linear growth and nutritional outcomes										
	HAZ		MUACZ		WAZ			WHZ or BAZ		
	Cont.	Int.	Cont.	Int.	Cont.	Int.		Cont.	Int.	
Baseline^a^	−3.8 [1.9]	−3.7 [1.8]	−3.4 [1.8]	−3.0 [1.7]	−3.9 [1.5]	−3.5 [1.4]		−2.6 [1.8]	−2.3 [1.7]	
*p*-value^d^	0.202		0.042		0.054			0.069		
Discharge^b^	−3.9 [1.9]	−3.7 [1.6]	− 3.2 [1.6]	−2.7 [1.4]	− 3.8 [1.4]	− 3.3 [1.3]		−2.0 [1.7]	−1.8 [1.4]	
*p*-value^d^	0.267		0.009		0.123			0.222		
End-line^c^	−3.7 [1.6]	−3.5 [1.4]	−1.2 [1.4]	−1.0 [1.4]	−2.5 [1.5]	−2.6 [2.2]		−0.5 [1.3]	− 0.6 [1.4]	
*p*-value ^d^	0.199		0.154		0.596			0.644		
Overall mean	−3.8 [1.8]	−3.6 [1.6	−2.6 [1.9]	−2.3 [1.7]	− 3.3 [1.6]	−3.2 [1.7]		−1.7 [1.8]	−1.6 [1.7]	

T-test used to determine *p*-value for mean difference

*BAZ* body-mass-index-for-age-z score, *Con* control, *Int* intervention, *HAZ* height or length-for-age-z score, *MUACZ* mid-upper-arm-circumference-for-age z score, *WAZ* weight-for-age -z score, *WHZ* Weight-for-height/length-z score, *SD* standard deviation

^a^before start of the psychomotor/psychosocial intervention; ^b^at discharge from hospital; ^c^after 6 months of home follow-up; ^d^*p*-value for a difference between the control and the intervention means

Age determines the scores on the four development outcomes assessed with Denver II-Jimma. However, age-matching was not possible at randomization because there was a rare possibility for simultaneous enrollment of children of similar ages into hospital. Therefore, we examined whether or not there was a significant association between the group to which the children were allocated and their age distribution across six age categories (< 6 mo., 12–24 mo., 24–36 mo. 36–48 mo., 48–60 mo. and 60–65 mo.). We found no statistically significant association except for age categories > = 48 mo., where more control than intervention children (20 vs 12., *p* = 0.001) were followed up (see Additional file 4: Table S1).

### Adherence to study protocol for the intervention group

Of the 113 intervention children completing the follow-up, five did not attend any of the planned hospital-based developmental stimulation sessions. Seventy (61.9%) children had less than the initially planned minimum individual playroom sessions because they left the hospital; either their caregivers wanted to leave for home, or physicians decided a discharge to avail treatment space for patients with more severe cases. Six children (5.3%) had less than three of the planned home visits.

### Effect of psychomotor-psychosocial stimulation on development, linear growth and nutritional status of SAM children

The results from the primary GEE model (without adjusting for baseline covariates) to estimate the effects of the intervention during (a) the hospital follow-up period, (b) the home-based follow-up period after discharge from hospital, and (c) during the whole follow-up period on primary and secondary outcomes (reflected in HAZ, MUACZ, WAZ and WHZ or BAZ scores) have been summarized (Table 4). There were some improvements over time for all outcomes (development, linear growth and nutritional status), but the improvement for the control and for the intervention groups differed significantly only in gross motor score during hospital follow-up

**Table 4:** Average increase ^a^ [95% CI] in developmental performance, linear growth and nutritional status from baseline to discharge, and from discharge to end-line and baseline to end-line, and effect size for significant intervention effect

Developmental outcomes									
Outcomes	Increase from baseline to discharge ^b^			Increase from discharge to end-line ^b^			Increase from baseline to end-line ^c^		
	Control group	Intervention effect	Effect size	Control group	Intervention effect	Effect size	Control group	Intervention effect	Effect size
Fine Motor	0.73^‡^ [0.28, 1.16] *p* < 0.001	0.31^**‡**^ [− 0.29, 0.94] *p* = 0.299	0.07	1.73^**‡**^ [1.1, 2.4] *p* < 0.001	0.85^**‡**^ [0.1, 1.6] *p* = 0.023	0.15^**†**^	2.50^**†**^ [1.9, 3.1] *p* = 0.001 0.38^¥^ [0.28, 0.47] *p* < 0.001	1.09^**†**^ [0.5, 1.7] *p* = 0.001 0.13^¥^ [0.01, 0.24] *p* = 0.033	0.22 ^Ÿ^
Gross Motor	0.36 ^**‡**^ [0.01, 0.7] *p* = 0.044 0.40^**†**^ [0.07, 0.74] *p* = 0.019	0.98^**‡**^ [0.5, 1.5] *p* = 0.001 0.88^**†**^ [0.40, 1.37] *p* < 0.001	0.26	3.9^**‡**^ [3.3, 4.5] *p* < 0.001	−0.6^**‡**^ [−1.5, 0.3] *p* = 0.222	− 0.20	4.3^‡^ [3.6, 4.9] *p* < 0.001 0.63^¥^ [0.54, 0.73] *p* < 0.001	0.4^‡^ [− 0.50, 1.3] *p* = 0.376–0.004^¥^ [− 0.14, 0.13] *p* = 0.948	0.06
Language	0.5^**‡**^ [0.1, 1.0] *p* = 0.022	0.01 [− 0.7, 0.7] *p* = 0.966	0.45	3.3 ^**‡**^ [2.5, 4.0] *p* < 0.001	0.5^**‡**^ [− 0.5, 1.5] *p* = 0.326	0.07	3.8^‡^ [3.0, 4.5] *p* < 0.001 0.55^¥^ [0.44, 66] *p* < 0.001	0.5^‡^ [− 0.5, 1.5] *p* = 0.329 0.07^¥^ [− 0.07, 0.21] *p* = 0.341	0.07
Personal Social	0.7 ^**‡**^ [0.3, 1.0] *p* = 0.001	−0.06^**‡**^ [− 0.6, 0.4] *p* = 0.812	−0.02	2.6^**‡**^ [2.0, 3.2] *p* < 0.001	0.12^**‡**^ [−0.7, 0.9] *p* = 0.762	− 0.03	3.2 ^‡^ [2.6, 3.8] *p* < 0.001 0.46^¥^ [0.37, 0.55] *p* < 0.001	0.06^‡^ [− 0.7, 0.8] *p* = 0.865 0.01^¥^ [− 0.11, 0.13] *p* = 0.887	0.011
Social-emotional Behavior	−12.8^**‡**^ [− 17.0, −8.5] *p* = 0.001	1.7^**‡**^ [−4.3, 7.7] *p* = 0.583	0.04	−1.8^**‡**^ [−7.6, 3.9] *p* = 0.256	− 4.3^**‡**^ [− 12.4, 3.8] *p* = 0.295	− 0.09	− 14.6^‡^ [− 20, −9.0] *p* < 0.001 1.78^¥^ [− 2.63, − 0.94] *p* < 0.001	−2.64^‡^ [− 9.2, 3.9] *p* = 0.426–0.36^¥^ [− 1.50, 0.78] *p* = 0.541	−0.09
Linear growth and nutritional outcomes									
Outcomes	Increase from baseline to discharge ^**b**^			Increase from discharge to end-line ^**b**^			Increase from baseline to end-line ^**c**^		
	Control group	Intervention effect	Effect size	Control group	Intervention effect	Effect size	Control group	Intervention effect	Effect size
HAZ	−0.05 ^**‡**^ [− 0.2, 0.1] *p* = 0.419	− 0.05^**‡**^ [− 0.1, 0.1] *p* = 0.633	−0.03	0.2^**‡**^ [0.01,0.4] *p* = 0.039	0.03^**‡**^ [−0.2, 0.3] *p* = 0.790	0.03	0.2 ^‡^ [−0.1,0.4] *p* = 0.138 0.03^¥^ [− 0.01, 0.07 *p* = 0.104	−0.004^‡^ [− 0.3, 0.3] *p* = 0.978–0.01^¥^ [− 0.05, 0.04] *p* = 0.753	−0.002
MUACZ	0.3^**‡**^ [0.1, 0.4] *p* = 0.002 0.34^**†**^ [0.24, 0.44] *p* < 0.001	0.2^**‡**^ [− 0.04, 0.35] *P* = 0.123 0.15^**‡**^ [− 0.04, 0.35] *p* = 0.123	0.12	1.9^**‡**^ [1.6, 2.2] *p* < 0.001	− 0.2^**‡**^ [− 0.5,0.2] *p* = 0.430	0.00	2.1^‡^ [1.8, 2.4] *p* < 0.001 0.34^¥^ [0.29, 0.39] *p* < 0.001	0.002^‡^ [− 0.4, 0.4] *p* = 0.999–0.02^¥^ [− 0.09, 0.05] *p* = 0.596	- < 0.001
WAZ	0.2^**‡**^ [0.03, 0.30] *p* = 0.022	0.1^**‡**^ [− 0.1, 0.3] *p* = 0.260	0.08	1.1^**‡**^ [0.9, 1.3] *p* < 0.001	− 0.3^**‡**^ [− 0.7,0.1] *p* = 0.155	−0.20	1.3^‡^ [1.0, 1.5] p = 0.001 1.30.19^¥^ [0.15, 0.23] *p* < 0.001	− 0.2^‡^ [− 0.7, 0.2] *p* = 0.372–0.06^¥^ [− 0.13, 0.01] *p* = 0.112	−0.02
WHZ or BAZ	0.5^**‡**^ [0.2, 0.8] *p* < 0.001	0.04^**‡**^ [−0.2, 0.3] *p* = 0.791	0.02	1.5^**‡**^ [1.1, 1.8] *p* < 0.001	−0.2^**‡**^ [− 0.6, 0.2] *p* = 0.321	− 0.04	2.0^‡^ [1.7, 2.3] *p* < 0.001 0.29^¥^ [0.24, 0.34] *p* < 0.001	−0.2^‡^ [− 0.5, 0.2] *p* = 0.352–0.06^¥^ [− 0.123, 001] *p* = 0.054	−0.06

In full model, parameter estimates are based on all significant and non-significant terms

In reduced model, parameter estimates are based on a parsimonious model that includes statistically significant terms and only essential non-significant model terms

*BAZ* body-mass-index-for-age z score, *GEE* Generalized Estimating Equations, *HAZ* height/length-for-age z score, *MUACZ* mid-upper-arm-circumference z score, *WAZ* weight-for-age z score, *WHZ* weight-for-height/length z score

^a^The increase for the control group during the follow-up time and the intervention effect (additional increase for the intervention group). When there is no intervention effect, the average increase of the control group and intervention group are equal

^b^A GEE model using time as a factor variable. As a factor variable (parameters marked with “^**‡, †**^”, time indicates the follow-up phases (hospital-based, home-based or a combination of both)

^c^A GEE model using time both as factor variable and as a continuous variable (parameters marked by “^¥^” . As a continuous variable, time indicates a mean change in the outcome variable when the duration of follow-up increases by 1 day)

^**‡**^ Obtained from a full model comprising time as factor variables (y = Time2 + Time3+ Time2*Treatment +Time3*Treatment, where Time1 = 0)

^**†**^ Obtained from a reduced model comprising time as factor variables

^¥^ Obtained from a full model comprising time as a continuous variable (y = Time + Treatment+ Time*Treatment)

**Fig. 2:**
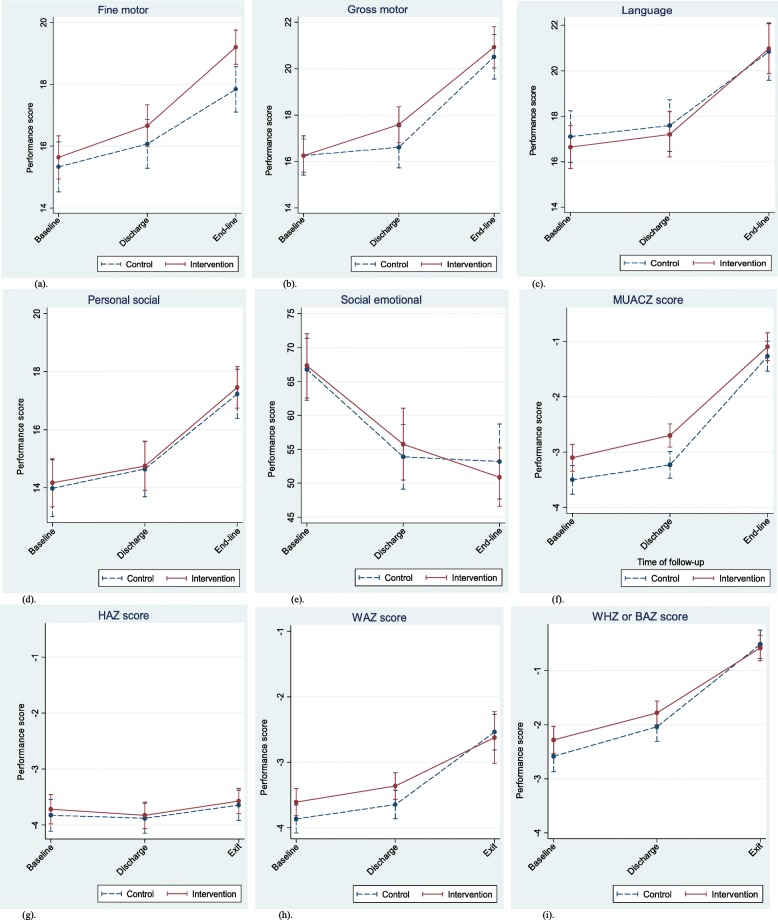
Developmental, linear growth and nutritional outcomes among the control and intervention SAM children during follow-up measurements (Figures were based on the basic GEE model comprising treatment, time (as indicator variable) and interaction between time and treatment)

and in fine motor score during the home follow-up period. The improvement in gross motor functions during the hospital follow-up period was higher in the intervention than the control group on average by 0.88 points (*p* < 0.001, effect size = 0.26 SD). The improvement in fine motor functions was higher for the intervention than the control group during the home based follow-up period on average by 1.09 points (*p* = 0.001, effect size = 0.15 SD), and each day during the whole follow-up period by 0.13 points (*p* = 0.033, effect size = 0.22 SD). No significant differences were observed between the two groups in linear growth (HAZ) and nutritional outcomes (MUACZ, WAZ and WHZ or BAZ) (Table 4, Additional file 5: Table S2 and Fig. 2).

### Moderation effects of baseline scores on treatment outcomes

There is no significant relationship of the treatment with child’s sex and baseline characteristics (age, developmental level, linear growth and nutritional status). The results are summarized in Table 5.

**Table 5:** Regression coefficients [95% CI] from the GEE models ^a^ with a continuous time scale (days in the study) depicting average change in developmental, growth and nutritional status during follow-up of the intervention

Explanatory variables			Developmental outcomes				
			FM	GM	LA	PS	SE
			β (95%CI)	β (95%CI)	β (95%CI)	β (95%CI)	β (95%CI)
Group ^b^			0.099 ^h^ [− 0.18, 0.38] *p* = 484	0.37 ^h^ [0.09, 0.65] *p* = 0.010	− 0.17 ^g^ [− 0.55, 0.21] *p* = 0.377	0.11 ^g^ [− 0.39, 0.16] *p* = 0.419	− 0.22 ^g^ [− 3.55, 3,11] *p* = 0.898
Time ^c^	Linear term		1.10 ^h^ [0.91, 1.21] *p* < 0.001	1.10 ^h^ [0.93, 1.24] *p* < 0.001	0.81 ^h^ [0.68, 00.95] *p* < 0.001	1.46 ^h^ [1.12, 1.80] *p* < 0.001	−4.30 ^g^ [− 10.11, 1.57] *p* = 0.151
	Quadratic term		−0.05 ^g^ [− 0.13, 0.02] *p* = 0.151	0.04 ^g^ [− 0.05, 0.12] *p* = 0.330	− 0.03 ^g^ [− 0.13, 0.06] *p* = 0.480	−0.06 ^g^ [− 0.10, − 0.01] *p* = 0.019	0.39 ^h^ [0.24, 0.54] *p* < 0.001
Baseline data	MUACZ		0.15 ^h^ [0.07, 0.24] *p* < 0.001	0.07 ^g^ [−0.02, 0.16] *p* = 0.127	–	0.13 ^h^ [0.06, 0.20] *p* < 0.001	−0.45 ^g^ [− 1.30, 0.41] *p* = 0.310
	WAZ		–	–	0.20 ^h^ [0.09, 0.31] *p* < 0.001	–	–
	Development		0.76 ^h^ [0.71, 0.81] *p* < 0.001	0.93 ^h^ [0.88, 0.98] *p* < 0.001	0.81 ^h^ [0.76,0.86] *p* < 0.001	0.87 ^h^ [0.83, 0.92] *p* < 0.001	0.86 ^h^ [0. 81, 0. 91] *p* < 0.001
	Age		0.06 ^h^ [0.04, 0.08] *p* < 0.001	0.03 ^h^ [0.01, 0.04] *p* < 0.001	0.08 ^h^ [0.05, 0.10] *p* < 0.001	0.04 ^h^ [0.02, 0.06] *p* < 0.001	0.13 ^h^ [0.04, 0.23] *p* = 0.008
Interactions of Time ^c^ with	Baseline development		−0.06 ^h^ [− 0.07, − 0.04] *p* < 0.001	− 0.06 ^h^ [− 0.07, − 0.04] *p* < 0.001	−0.06 ^h^ [− 0.08, − 0.05] *p* < 0.001	−0.07 ^h^ [− 0.08, − 0.06] *p* < 0.001	−0.08 ^h^ [− 0.09, − 0.06] *p* < 0.001
	Baseline Age		0.01 ^h^ [0.001, 0.012] *p* = 0.018	0.02 ^h^ [0.01, 0.022] *p* < 0.001	0.03 ^h^ [0.02, 0.04] *p* < 0.001	0.014 ^h^ [0.001, 0.02] *p* = 0.001	0.04 [0.01, 0.06] *p* = 0.010
Interactions of Group with	Time ^c^ (Linear)		0.15 ^g^ [0.07, 0.22] *p* < 0.001	0.41 ^g^ [− 0.44, 1.27] *p* = 0.344	0.02 ^g^ [− 0.92, 0. 96] *p* = 0.971	− 0.06 ^g^ [− 0.72, 0.59] *p* = 0.850	1.63 ^g^ [−6.73, 9,98] *p* = 0.703
	Interaction of Sex ^f^ with Time (Linear)	Control female	− 0.02 ^g^ [− 0.13, 0.08] *p* = 0.657	−0.07 ^g^ [− 0.20, − 0.06] *p* = 0.302	−0.08 ^g^ [− 0.21, 0.05] *p* = 0.240	−0.004 ^g^ [− 0.1, 0.09] *p* = 0.940	0.29 ^g^ [− 0.92, 0.50] *p* = 0.638
		Intervention Female	0.01 ^g^ [− 0.09, 0.11] *p* = 0.827	− 0.02 ^g^ [− 0.14, − 1.00] *p* = 0.742	0.04 ^g^ [− 0.08, 0.17] *p* = 0.525	−0.08 ^g^ [− 0.17, 0.01] *p* = 0.086	−0.47 ^g^ [− 1.60, 0.67] *p* = 0.421
	Interaction of Baseline development with Time (Linear)		0.02 ^g^ [−0.01, 0.04] *p* = 0.206	− 0.02 ^g^ [− 0.05, 0.003] *p* = 0.086	0.004 ^g^ [− 0.02, 0.03] *p* = 0.765	−0.02 ^g^ [− 0.04, 0.004] *p* = 0.102	−0.02 ^g^ [− 0.04, 0.01] *p* = 0.236
	Interaction of Baseline Age with Time (Linear)		−0.005^g^ [− 0.01, 0.004] *p* = 0.253	0.01 ^g^ [− 0.004, 0.01] *p* = 0.278	0.001 ^g^ [− 0.01, 0.01] *p* = 0.889	0.01 ^g^ [− 0.001, 0.02] *p* = 0.072	−0.01 ^g^ [− 0.07, 0.06] *p* = 0.861
			Linear growth ^d^ and nutritional ^**e**^ outcomes				
Explanatory variables			HAZ	MUACZ	WAZ		WHZ/BAZ
Group ^b^			−0.26 ^g^ [− 0.57, 0.06] *p* = 0.101 *p* = 0.113	0.07 ^g^ [− 0.12, 0.26] *p* = 0.461	−0.215 ^g^ [− 0.05, 0.47] *p* = 0.119		0.22 ^g^ [− 0.13, 0.58] *p* = 0.214
Time ^c^	Linear term		− 0.09 ^h^ [− 0.11, − 0.07] *p* < 0.001	0.19 ^g^ [− 0.05, 0.44] *p* = 0.122	0.28 ^g^ [0.04, 0.53] *p* = 0.025		0.33 ^h^ [0.07, 0.59] *p* = 0.008
	Quadratic term		0.004 ^g^ [− 0.03, 0.02] *p* = 0.765	−0.02 ^g^ [− 0.05, 0.02] *p* = 0.360	−0.03 ^g^ [− 0.06, 0.001] *p* = 0.061		−0.04 ^h^ [− 0.08, − 0.001] *p* = 0.045
Baseline data	HAZ		0.85 ^h^ [0.81, 0.88] *p* < 0.001	0.004 ^g^ [− 0.02, 0.03] *p* = 0.727	0.07 ^h^ [0.01, 0.13] *p* = 0.024		0.02 ^g^ [− 0.10, 0.14] *p* = 0.733
	MUACZ		–	0.92 ^h^ [0.88, 0.96] p < 0.001	–		–
	WAZ		0.13 ^h^ [0.08, 0.17] *p* < 0.001	0.08 ^h^ [0.04, 0.13] *p* = 0.001	0.85 ^h^ [0.79, 0.90] *p* < 0.001		0.004 ^g^ [− 0.22, 0.21] *p* = 0.972
	WHZ or BAZ		–	–	–		0.82 ^g^ [0.75, 0.88] *p* < 0.001
	Age		−0.005 ^g^ [− 0.01, − 0.001] *p* = 0.011	−0.001 ^g^ [− 0.003, 0.003] *p* = 0.949	0.002 ^g^ [− 0.001, 0.004] *p* = 0.219		0.01 ^h^ [0.001, 0.010] *p* = 0.012
Interactions of Time ^c^ with	Baseline HAZ		−0.033 ^h^ [− 0.038, − 0.028] *p* < 0.001	0.01 ^g^ [− 0.01, 0.04] *p* = 0.202	0.01 ^g^ [− 0.01, 0.04] *p* = 0.265		0.005 ^g^ [− 0.03, 0.04] *p* = 0.782
	Baseline MUACZ		–	− 0.09 ^h^ [− 0.10, − 0.08] *p* < 0.001	–		–
	Baseline WAZ		0.01 ^g^ [− 0.04, 0.05] *p* = 0.800	− 0.01 ^g^ [− 0.04, 0.02] *p* = 0.556	− 0.042 ^h^ [− 0.05,-0.036] *p* < 0.001		0.02^g^ [− 0.04, − 0.07] *p* = 0.510
	Baseline WHZ or BAZ		–	–	–		− 0.09 ^h^ [− 0.11, − 0.07] *p* < 0.001
	Baseline Age		0.001 ^g^ [− 0.002, 0.004] *p* = 0.547	−0.001 ^g^ [− 0.004, 0.002] *p* = 0.589	−0.001 ^g^ [− 0.003, 0.001] *p* = 0.328		0.0002 ^g^ [− 0.003, 0.002] *p* = 0.824
Interactions of Group with,	Interaction between Time ^c^ and baseline HAZ		0.02 ^g^ [− 0.01, 0.05] *p* = 0.260	–	–		–
	Interaction between Time and baseline MUACZ		–	−0.01^g^ [− 0.005, 0.05] *p* = 0.879	–		–
	Interaction between Time and baseline WAZ		–	–	−0.001^g^ [− 0.05, 0.05] *p* = 0.970		–
	Interaction between Time and baseline WHZ or BAZ		–	–	–		0.02 ^h^ [0.01, 0.04] *p* = 0.012
	Interaction between Time and sex	Female control	−0,02 ^g^ [− 0.08, 0.05] *p* = 0.594	−0.04 ^g^ [− 0.12, 0.05] *p* = 0.408	−0.05 ^g^ [− 0.12, 0.02] *p* = 0.136		−0.03 ^g^ [− 0.12, 0.06] *p* = 0.514
		Female Intervention	−0.004 ^g^ [− 0.05, 0.04] *p* = 0.868	−0.06 ^g^ [− 0.12, 0.01] p = 0.102	−0.05 ^g^ [− 0.17, 0.06] *p* = 0.389		−0.01 ^g^ [− 0.08, 0.06] *p* = 0.819

Only significant terms (at *p* < 0.05) were kept in the models

*BAZ* body-mass-index-for-age z score, *FM* fine motor, *GEE* Generalized Estimating Equations, *GM* gross motor, *HAZ* height/length-for-age z score, *LA* language, *MUACZ* mid-upper-arm circumference -for-age z score, *PS* personal social, *SE* social-emotional, *WAZ* weight-for-age z score, *WHZ* weight-for-height/length z score

^a^ Intervention effect controlling for baseline score, baseline characteristics, and interactions with time. ^b^ Refers to the intervention group (treatment)

^c^ ‘Time’ refers to number of days in the study (duration of follow-up); ^d^ ‘growth’ refers to baseline linear growth expressed in HAZ score

^e^‘nutritional’ refers to baseline nutritional status expressed in MUACZ score and WAZ score. ^f^ sex; the reference is female

^g^ Parameter estimates obtained from a full model by simultaneously including all potential explanatory variables

^h^ Parameter estimates obtained from a reduced model with statistically significant explanatory variables along with only essential non-significant model terms

The treatment bears no relationship to the child’s sex, baseline age and baseline developmental scores, linear growth, and nutritional status measured on MUACZ and WAZ scores. However, it is related to baseline WHZ or BAZ score: for a similar length of follow-up, the intervention children with better baseline WHZ or BAZ score benefitted more from the intervention (β = 0.02, *p* = 0.012) (Table 5).

### Factors affecting performance of the intervention children

Some factors which were thought to affect the performance within the intervention SAM children were examined. After adjusting for some child conditions (health status, baseline social-emotional scores), a significant association was observed between access to RUTF and performance on gross motor and language (Table 6). Intervention children who were reported to sometimes be receiving RUTF from nearby health centers scored significantly higher in language (β = 1.41, *p* = 0.032) than the children who rarely or never received RUTF at all. Children who were reported to have always been receiving/collecting RUTF from nearby health center scored significantly lower in GM (β = − 0.93, *p* = 0.031). This might refer to few children who were in critical need of more RUTF because their condition did not improve. These are among only 22.6% of the children, because interviews to caregivers at the final home visit showed that 73.4% the intervened SAM children did not receive RUTF at all. Compared to those who were reported to be sick, SAM children who were not sick during the home follow-up scored significantly higher on personal social (β = 0.5092, *p* = 0.047) but lower on language (β = − 1.11, *p* = 0.024). Lower SE score (i.e., better behavior) predicts better outcomes on FM. With an increase in SE score (which marks more problem behaviors), performance on FM decreased significantly (β = − 0.02, *p* = 0.016).

**Table 6:** Regression coefficients [95% CI] from the GEE models ^a^ examining some factors modifying the effect of the intervention on developmental outcomes

Explanatory variables		Developmental outcomes				
		FM	GM	LA	PS	SE
		β (95%CI)	β (95%CI)	β (95%CI)	Β (95%CI)	β (95%CI)
Duration of follow up (days in study)		0.45 [0.39, 0.52] *p* < 0.001	0.57 [0.47, 0.68] *p* < 0.001	0.62 [0.52, 0.72] *p* < 0.001	0.49 [0.41, 0.56] *p* < 0.001	−1.7 [− 2.58, − 0.75] *p* < 0.001
Number of stimulation sessions in hospital ^b^		0.07[− 0.07, .22] *p* = 0.329	0.07 [− 0.1,0.2] *p* = 0.415	0.08 [− 0.3, 0.5] *p* = 0.691	0.06 [−0.1, 0.2] *p* = 0.530	0.97 [0.07, 0.19] *p* = 0.034
Two or more ^c^ stimulation sessions at home		−0.2 [− 0.7, 0.3] *p* = 0.432	−0.001[− 0.6, 0.6] *p* = 0.998	−0.7 [− 1.9, 0.5] *p* = 0.249	−0.2 [− 0.7, 0.4] *p* = 0.571	− 3.3 [− 9.6, 2.95] *p* = 0.301
How often ^d^ the intervention SAM child received RUTF from a nearby health center	Always	−0.5 [− 1.1, 0.3] *p* = 0.234	−0.93 [− 1.78, − 0.09] *p* = 0.031	0.1 [− 1.1, 1.4] *p* = 0.842	− 0.1 [− 0.7, 0.5] *p* = 0.714	− 0.8 [− 6.9, 5.2] *p* = 0.789
	Sometimes	− 0.2 [−1.1, 0.7] *p* = 0.727	− 0.6 [− 1.3, 0.1] *p* = 0.112	1.41 [0.12, 2.70] *p* = 0.032	− 0.4 [− 1.1, 0.3] *p* = 0.281	1.3 [− 7.1, 9.6] *p* = 0.767
Whether or not the intervention SAM child was sick after discharge from hospital ^e^		0.09 [− 0.5, 0.7] *p* = 0.781	0.2 [− 0.5, 0.9] *p* = 0.520	− 1.11 [− 2.10, − 0.15] *p* = 0.024	0.51 [0.01, 1.01] *p* = 0.047	− 3.3 [− 9.6, 2.9] *p* = 0.297
Whether or not the intervention SAM child was getting home-prepared special food during the home follow-up		−0.2 [− 1.0, 0.5] *p* = 0.552	0.6 [− 0.05, 1.2] *p* = 0.071	−0.5 [− 1.6, 0.6] *p* = 0.414	−0.1[[− 0.5, 0.4] *p* = 0.789	− 3.4 [− 9.2, 2.4] *p* = 0.255
Social-emotional scores of the child during the follow-up		−0.02* [− 0.03, − 0.003] *p* = 0.016	−0.01 [− 0.03, 0.001] *p* = 0.068	−0.01 [− 0.03, 0.02] *p* = 0.543	−0.003 [− 0.01, 0.01] *p* = 0.544	–

*FM* fine motor, *GEE* Generalized Estimating Equations, *GM* gross motor, *LA* language, *PS* personal social, *SE* social-emotional

^a^ All the covariates were entered simultaneously into the GEE model for each developmental domain

^b^ ‘Stimulation sessions’ refers to total number of play sessions in the play room and playground the intervention SAM child received before discharge from hospital; ^c^ reference is only ‘once a day’; ^d^ reference is ‘rarely or not at all’

^e^ The reference is “being sick”

When entered alone in the model [not reported in table], the number of play sessions that the intervention SAM child received in hospital was associated significantly with all developmental outcomes, except with social-emotional scores. However, when entered simultaneously with other covariates, more play sessions were associated with higher social-emotional score (mean = 0.97, 95%CI [0.08, 0.19]), indicating that the children who stayed longer in hospital and attended more play sessions were those with more problem behaviors. When entered separately in the model, the duration of follow-up predicted all outcomes. Longer follow-up predicted better developmental, linear growth, and nutritional outcomes (see Additional file 6: Table S3).

Finally, a comparison with age-matched healthy children showed that both the control and the intervention SAM children could not catch up after more than 6 months of follow-up, except on social-emotional behavior, in which both groups did not differ from the healthy ones (see Additional file 7: Table S4).

## Discussion

The study shows play-based psychomotor/psychosocial stimulation benefits the motor (fine and gross) development of SAM children of 6 months to 6 years of age. For the gross motor functions, the intervention effect was significant during the shorter hospital-based period (12.1 ± 8.95, range 1–48 days), but the effect became insignificant later during the 6 months of home follow-up. For the fine motor functions, the intervention effect became significant during the home follow-up. The effect of the intervention across the whole follow-up period was examined using analysis that combined both the hospital- and home-based follow-up periods, and showed a significant improvement in only the fine motor functions. The effect of the intervention was similar for both the younger and the older children. A non-significant but positive trend of improvement was observed in the other developmental domains (GM, LA, PS and SE) (Additional file 5: Table S2). The intervention did not improve linear growth and nutritional outcomes. There was a high loss to follow-up in the study. More loss has been observed in the control than in the intervention group. The high loss in the control group could be attributed to the lack of frequent contact and support. On the other hand, the home visits after discharge from hospital and provision of play materials might have encouraged more SAM children in the intervention group to remain in the study. Different statistical approaches other than those initially planned were applied to examine if there was a significant influence from the high loss-to-follow-up. The findings from three methods (complete case analysis, direct likelihood and multiple imputation) were compared using linear mixed model, and showed the same conclusion.

An interesting finding is the difference in response of the gross motor and the fine motor functions to the intervention in different settings. The treatment effect on the gross motor skills was attained only during the hospital-based follow-up. For the SAM children, the diet at the nutritional rehabilitation unit of the hospital is not only better in quality, quantity and diversity, but is also provided at appropriate times with strict supervision of professionals on a daily basis. Moreover, caregivers dedicate their time fully to the child and remain in closer contact. This is not the case in a home setting, where diet is inadequate, less diversified, non-balanced and also not served in a timely manner, since caregivers have other family responsibilities. The improvement might have been enhanced by the combined and synergistic effect of the psychomotor stimulation and the dietary therapy. The stimulation may facilitate the gross motor functions to be reactivated as soon as the large muscles regain strength from dietary rehabilitation. The fact that this effect became non-significant during the home-based follow-up could be attributed to the lack of adequate essential nutrients in the diet at home. At home, the children lack the standard daily nutritional care provided at the NRU of the hospital. The inverse relationship between gross motor performance and more frequent collection of RUTF from nearby health center seems to contradict this finding. However, collection does not ensure provision of the RUTF to the child. Usually, caregivers who have serious food shortages collect and share it among other children in the family [32, 33]. Since such children do not get the required amount, the implication of frequent collection of RUTF has to be interpreted carefully. There were challenges related to the utilization of RUTF from nearby health centers. In some cases, there were shortages of RUTF, and in other cases the RUTF collected was shared among other children in the family. In some other cases, parents could not regularly collect the RUTF due to the lack of a health center in the vicinity. An interview with 112, 105 and 109 caregivers during the first, second and third (final) home visit respectively showed that 42.9, 54.3 and 73.4% of the intervened SAM children did not receive RUTF at all. If children were not able to obtain an adequate diet, they may not have been active enough to engage in intensive motor activities. Therefore, this might have also reduced the gross motor stimulation at home, and thereby resulted in a lack of significant intervention effect.

On the other hand, the significant intervention effect observed on fine motor only at a later time (during the home follow-up) might be explained in two ways. Firstly, unlike the gross motor activities, which demand relatively more physical strength and energy, the fine motor activities might be less demanding and more feasible for SAM children to engage in and practice. Secondly, it might reflect fine motor’s slower response to the intervention. Recovery in fine motor functions might require more than a mere recovery of muscles: more integration of the muscles and the brain functions through a gradual process of holistic recovery in both physical and emotional states. There have been inconsistent findings with regard to the effect of stimulation on motor outcomes. Psychosocial stimulation improved fine motor but not gross motor outcomes [12], and stimulation, with and without food supplementation had no effect on motor outcomes [18].

Though non-significant, the positive trend of improvement observed in the other areas of development could be related to such a gradual recovery process. The duration of the intervention in the present study might have been too short to generate a significant effect, as indicated in an earlier study [18]. Moreover, the intervention was not intensive. A maximum of only three home visits over a period of 6 months is not sufficient to provide adequate stimulation to the child and equip the mostly illiterate caregivers with effective stimulation techniques. Studies have shown a marked improvement in performance as the frequency of home visit increases [34, 35]. Interventions targeting high-risk children, such as those who are malnourished, might need more frequent home visits to be more beneficial [36]. None of the studies conducted so far on the stimulation of SAM children has used so few home visits as the present study. It is argued that the impact of short stimulation programs during malnutrition episodes is temporary, particularly in conditions of extreme poverty to which the children return [37]. A depriving home environment and poor nutrition remain detrimental to the outcomes of the intervention. This is the case with the present study. All families of the SAM children belong to low socio-economic groups and live in poor home environments, all of which could compromise the care and attention that the SAM child’s needs.. In general, a minimum number of parenting sessions needs to be offered to sustain change when children are in an acute state and parents are challenged to provide needed stimulation.

Inconsistencies across studies on some outcomes could be related to differences in study design, duration of follow-up, the children’s age and nutritional status, poverty level and maternal education, assessment tools used, and quality and intensity of home stimulation, and support from the health system.

Though their findings are inconsistent, earlier studies have targeted children under 24 months of age, or investigated the long-term effects of interventions which started at an early age and then continued later for some years [38–44]. In the present study, children 6 months to 6 years were included. It is assumed that young children are likely to have more opportunities for mother-child interactions, better stimulation, and breast-feeding, which could buffer them against extreme malnutrition. However, the present study, which addressed children of more heterogeneous ages, has shown that the treatment effect does not depend on age. It showed that both younger and older children responded similarly to the treatment, implying the potential benefits of psychosocial/psychomotor stimulation to both younger and older children under the age of six. The present study also showed that there is no significant difference in the effect of the intervention based on sex of child. But the reason for a higher loss to follow-up among boys than girls needs further investigation.

At baseline, SAM children showed marked problems in all domains of development. Gross motor was found to be the worst affected domain [45] After more than 6 months of follow-up, both the control and the intervention groups could not catch up with healthy children on nutritional and the four developmental outcomes (FM, GM, LA and PS). However, the social-emotional behavior improved markedly and was similar to that of healthy children (Additional file 6: Table S3). Studies have already shown that SAM children have behavioral abnormalities during the acute stage, which spontaneously improve during the course of recovery [37]. The improvement in the present study followed a non-linear trend. A rapid improvement during the first 4 months of follow-up seems to have slowed down afterwards (Additional file 3: Figure S1). Compared to the control SAM and the age-matched healthy children, the intervention SAM children showed substantially lower social-emotional problems (Additional file 6: Table S3).

Nonetheless, the result of our study has to be interpreted with some constraints and limitations. First, it was not possible to implement the intervention as planned. During hospital follow-up, the majority of children did not receive adequate stimulation sessions. Neither did their caregivers undergo sufficient education and training. This was because the SAM children had to leave the hospital and follow the treatment as out-patients in line with a protocol for the management of SAM children. The situation of some families also influenced the home-based stimulation. After home visits, supervisors and intervention workers often reported the presence of low motivation to engage in play among SAM children and families facing severe dietary problems. Consequently, the intervention package was less intensive and could not be implemented as planned. Second, the possibility for a contamination effect cannot be ruled out for this study. For ethical reasons, a playground meant for the study was accessible to all children. Moreover, there was a possibility for intervention and control children and their caregivers to stay in the same hospital bedroom and thus share information. Even after discharge from hospital, it was also possible that caregivers shared information, since randomization into control and intervention groups did not take the address of the child into account. Third, the study did not address a specific type of severe acute malnutrition, since enrollment was based on broader admission criteria. Hence, children may have edematous malnutrition, severe wasting, a combination of both, and/or a combination of stunting and wasting. Therefore, the severity of the nutritional deficiency may have varied among the study subjects. As a result, they may have responded differently to the intervention. An earlier study indicated that the presence of heterogeneous groups of conditions is a factor that constrains the interpretation of literature regarding severe acute malnutrition, and that the outcome of intervention depends to a large extent on the quality of the subsequent environment [37]. The fourth limitation is related to the sample size of the study. The statistical power computed from the cross-sectional data is insufficient to power the group*time interaction effect examined in the longitudinal data set. Ideally, a pilot study or an established literature is needed to determine a more accurate sample size. The present study lacks either of the two, and is a sort of ‘hypothesis generating type’. Another limitation of the study is related to estimation of the size of the intervention effect. The GEE model estimates the intervention effect by including information of missing cases. However, the effect size calculated for the gross motor used complete case analysis due to the dependency between baseline and discharge scores. The lack of baseline and endline information on the child’s diet and quality of home environment stimulation measured on the Home Observation Measurement of the Environment (the HOME) are also among the other limitations.

Regardless of its limitations, the study has some strengths. The involvement of a multidisciplinary team of local and European professionals and practitioners enabled the development of a contextually relevant intervention program integrating theory with practice. Developmental outcomes were assessed using an adapted tool, and another one adapted and standardized in the study context. The home visits and support provided at an individual family level not only allowed a single caregiver to work with the SAM child, but also with all family members, and in some case immediate neighbors and peer groups. It also provided in-depth qualitative data for a better understanding of the child’s real context, and the challenges impeding the practical implementation of the intervention program. Besides including children of wider age ranges, it is the first randomized controlled trial attempted to examine the effect of adding psychomotor/psychosocial stimulation in the treatment of SAM children both at the in-patient and the out-patient phases under the real circumstances of different family settings.

## Conclusions

This study has shown that play-based stimulation contributes in the treatment of SAM children under six in low-income settings. Stimulation significantly improved the gross motor functions during the hospital stay, and the fine motor functions after discharge during the home follow-up. Both younger and older children benefited similarly from the intervention. The intervention effect on fine motor functions is, however, small. This could be due to the lack of access to an adequate and balanced diet at home, non-intensive stimulation, the short period of follow-up, and non-adherence by caregivers to strictly implement the home-based stimulation. Though the effect of the intervention is small, attaining it in such a context with limited support and resources shows that it has the potential to bring a better effect. On the other hand, the positive trends of improvement in other developmental areas show a promising effect of the intervention. It shows the possibility of designing a simple, feasible and cost-effective mechanism to engage families in a low-income context in ameliorating the damaging effect of SAM on young children. Future studies need to find the right model for improving motor, nutritional and mental status of very deprived children. Intervention packages ensuring access to balanced diets and extending longer than 6 months in duration might be better to uncover particularly the gradual developmental changes in older children.

## Additional files

Additional file 1: Highlight of the psychomotor/ psychosocial stimulation program. (PDF 1450 kb)

Additional file 2: Dataset file. (CSV 466 kb)

Additional file 3: **Figure S1** Developmental outcomes of the control and intervention SAM children during 6 months of follow-up (DOCX 36 kb)

Additional file 4: **Table S1** Baseline and end-line developmental performance and WAZ scores of SAM children ^a^ followed up for 6 months after discharge from hospital compared with healthy children ^b (DOCX 17 kb)^

Additional file 5: **Table S2** The overtime change ^**a**^ in developmental ^**b**^ performance, linear growth ^**c**^ and nutritional status ^**d**^ of SAM children during a follow-up in hospital as an in-patient and at home after discharge for a period of 6 months (DOCX 20 kb)

Additional file 6: **Table S3** Regression coefficients [95% CI] from the GEE models ^a^ separately examining some selected factors modifying the effect of the intervention on development, linear growth and nutritional outcomes (DOCX 18 kb)

Additional file 7: **Table S4** Baseline and end-line developmental performance and WAZ scores of SAM children ^a^ followed up for 6 months after discharge from hospital compared with healthy children ^b^ (DOCX 18 kb)

## Data Availability

The datasets analyzed during the current study are submitted as a supplementary file (Additional file 2).

## Abbreviations

BAZ: Body-mass-index-for-age z score
FM: Fine motor adaptive
GM: Gross motor
HAZ: Height/length-for-age z score
LA: Language
mo: Month
MUAC: Mid-upper arm circumference
MUACZ: Mid-upper arm circumference Z-score
NRU: Nutrition rehabilitation unit
PS: Personal social
SAM: Severe acute malnutrition
SD: Standard deviation
WHO: World Health Organization
WHZ: Weight-for-height/length-for-age z score
