# Supplementary material for: Effect of play-based family-centered psychomotor/psychosocial stimulation on the development of severely acutely malnourished children under six in a low-income setting: a randomized controlled trial
Source: BMC Pediatr. 2019 Sep 14;19:336. doi: 10.1186/s12887-019-1696-z (PMC6744679; doi:10.1186/s12887-019-1696-z)
Supplement: Supplementary file 1 — Highlight of the psychomotor/ psychosocial stimulation program. (PDF 1450 kb) [file 12887_2019_1696_MOESM1_ESM.pdf]

Developmental stimulation program Jimma, Ethiopia

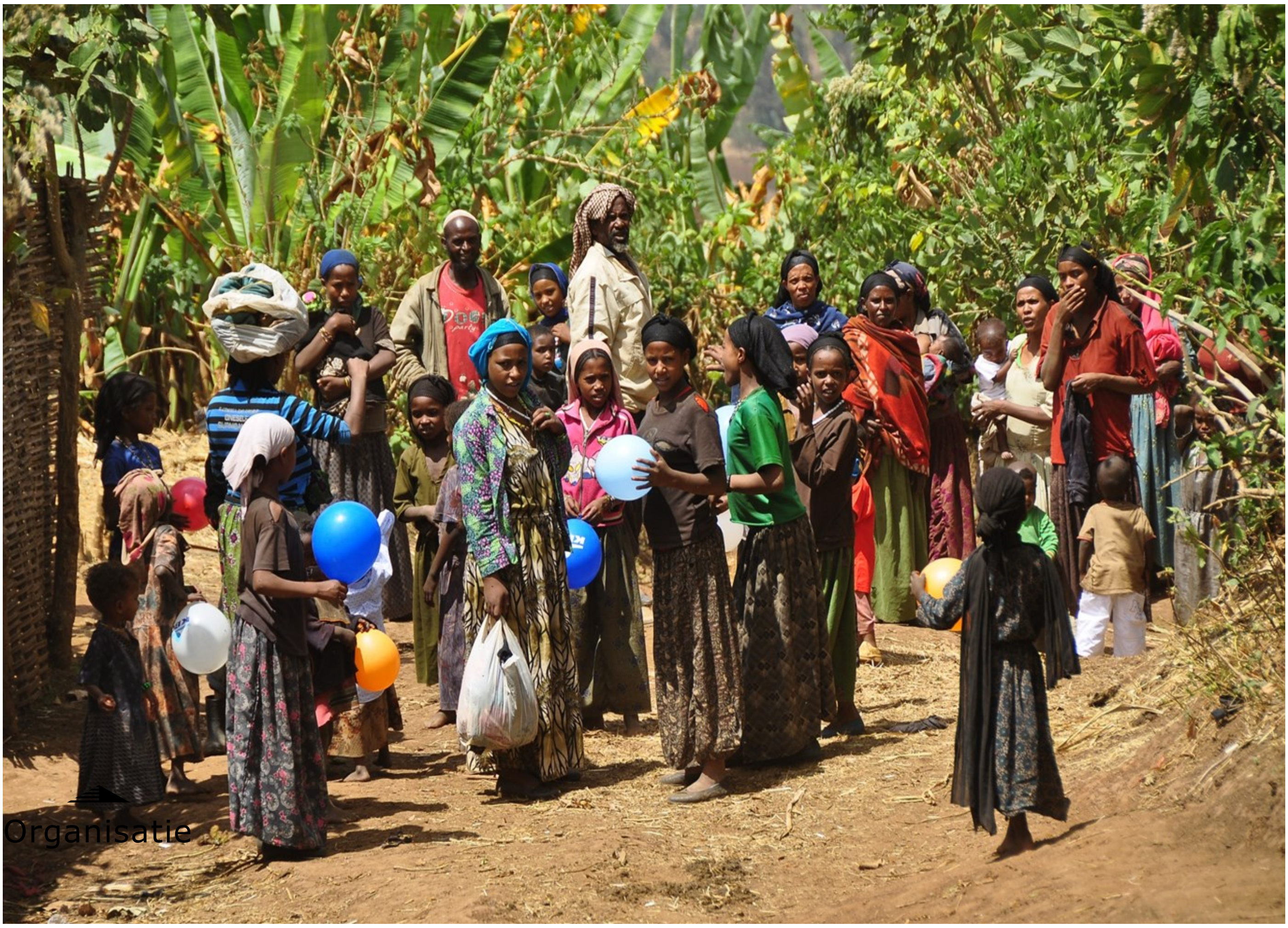

Sharing minds, changing lives

History

In 2009, an interuniversity collaboration between Jimma University (Ethiopia) and the University Association Hasselt and Ghent University (Belgium) was started on child health and nutrition. One of its subprojects is a psychomotor stimulation study.

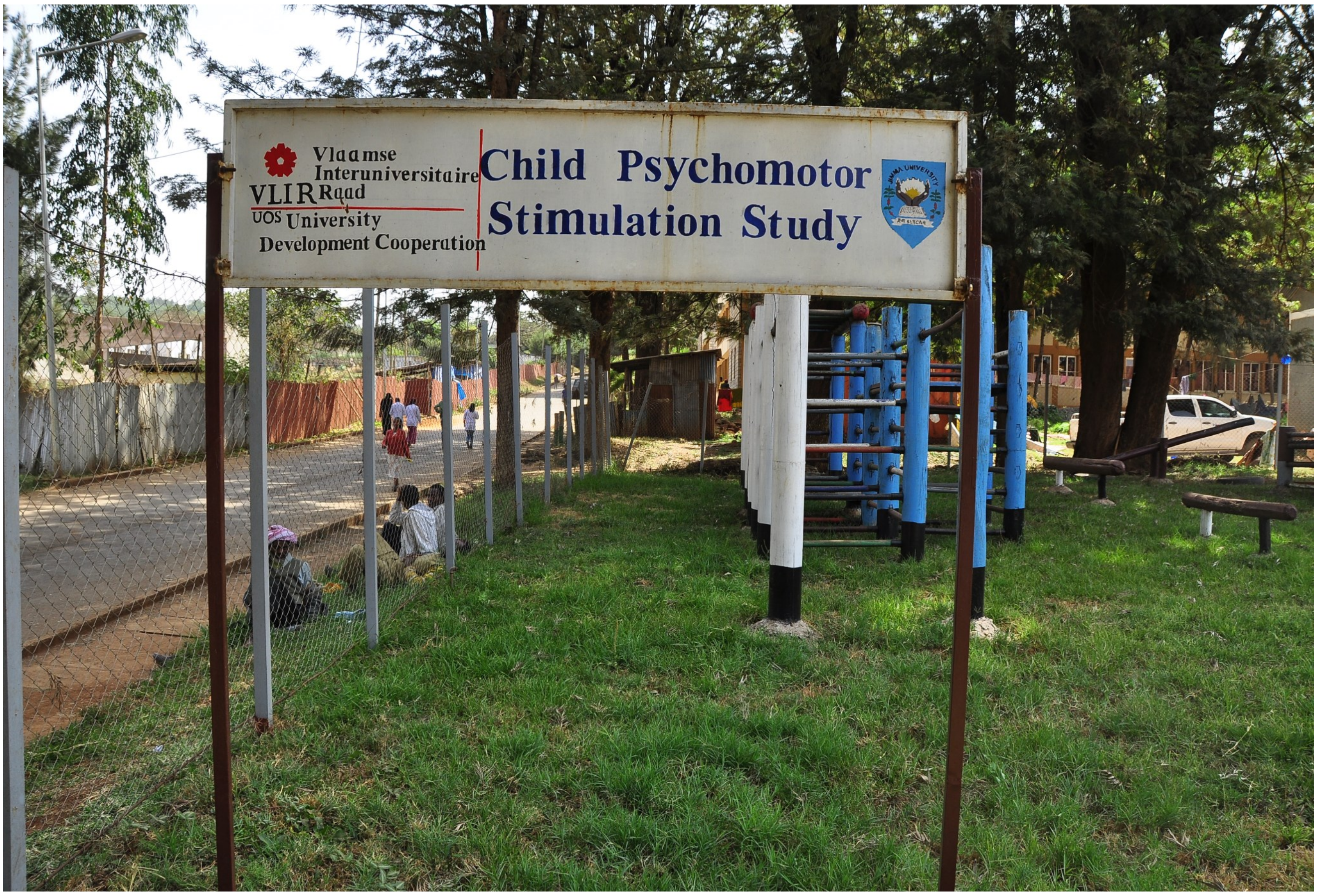

Malnourished children are known to have an important psychomotor deficit. Therefore, the study wanted to investigate to what degree family-oriented psychomotor/ psychosocial play activity could stimulate the recovery of growth and development of acutely malnourished children aged 6-60 months in the Jimma region of Ethiopia.

A team of the University Association Hasselt (Belgium) started up the research with local experts in the area of developmental delays. In the first stage, the Denver Developmental Screening Test was adapted to the local context (Denver II Jimma) and a reference was established with local children. In the second stage, a stimulation program was developed that focusses both on the child and its environment.

Local health workers (the testers) were trained to screen with the adapted Denver II Jimma test. Other health workers were trained in implementing the stimulation program in the family.

Adapting the Denver II Jimma

The original Denver II test was selected for its ease in application and short screening time. Health workers can easily be trained to do the testing. The adaptation to the local context was done with multiple discussion sessions and test performances with local

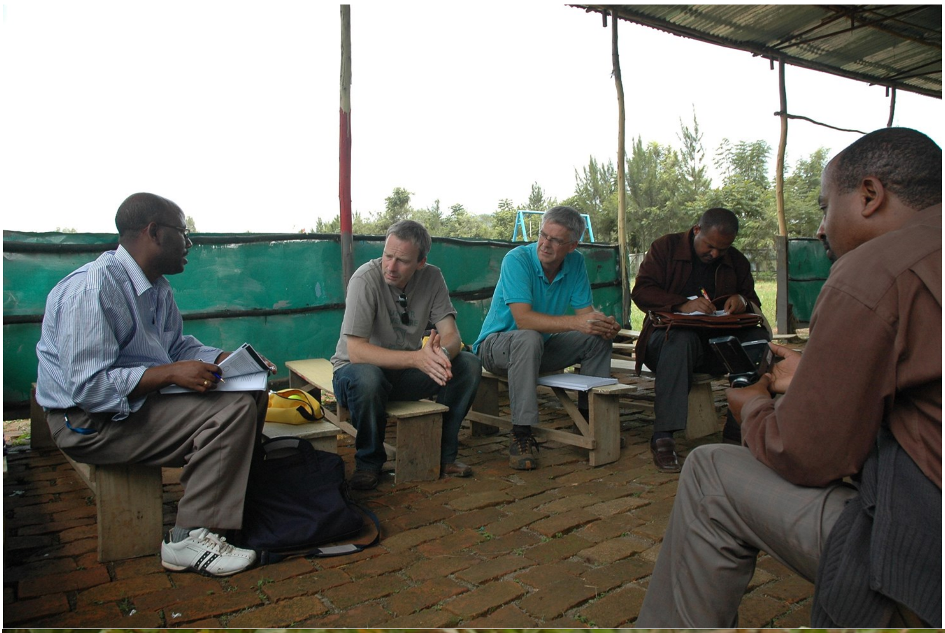

psychologists. This resulted in the Denver II Jimma. To validate the test, 1400 children were tested so that a reference value set could be made. Urban children from the Jimma area in Ethiopia served as reference children.

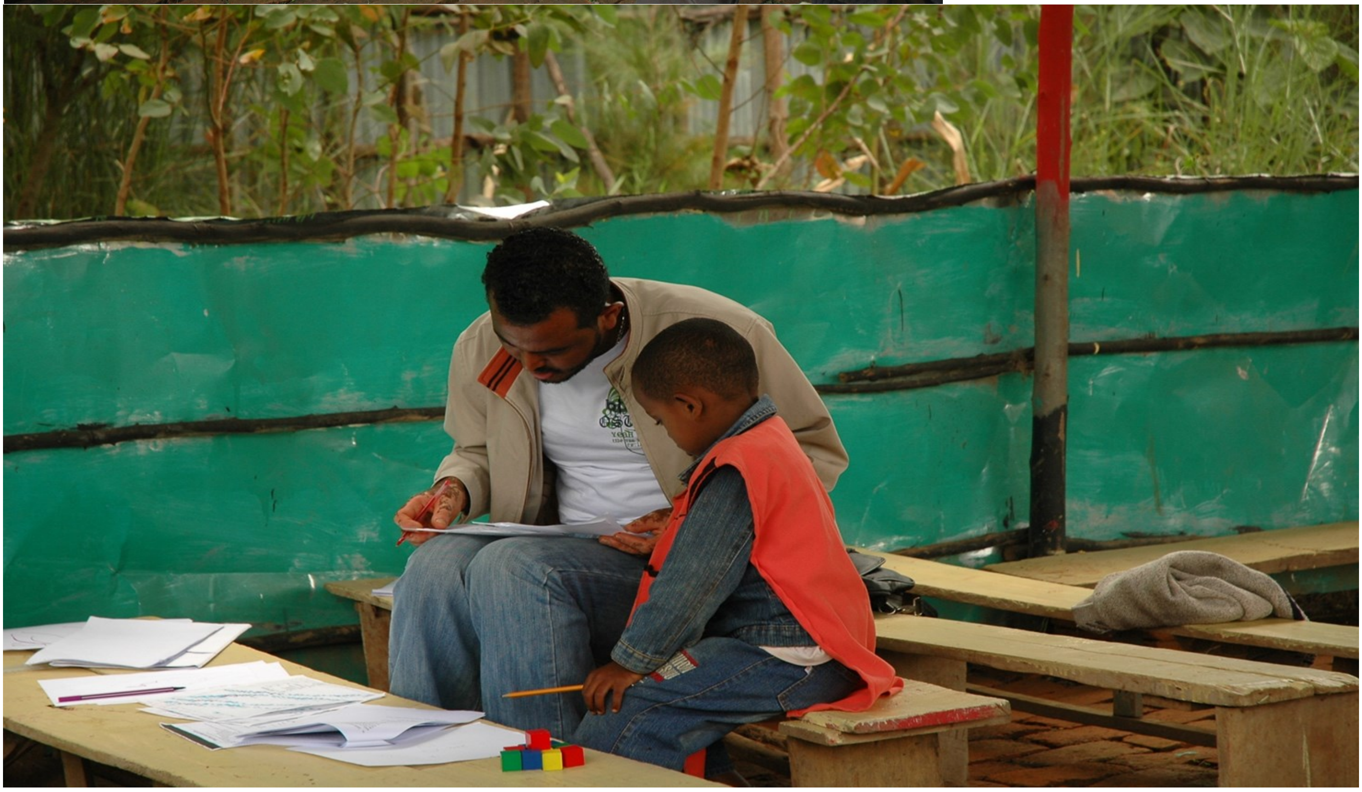

Vision child development

The stimulation program was developed by using the Nurture Nature theory of Khan as the entry reflection point on neuro-psychological development. The dynamical systems approach of Swaab identifies the period between conception and 6 years of age as the most sensitive period. Development is made possible when a child is placed in a developmentally stimulating environment. A good example is language acquisition. This is only possible when the child is in contact ….

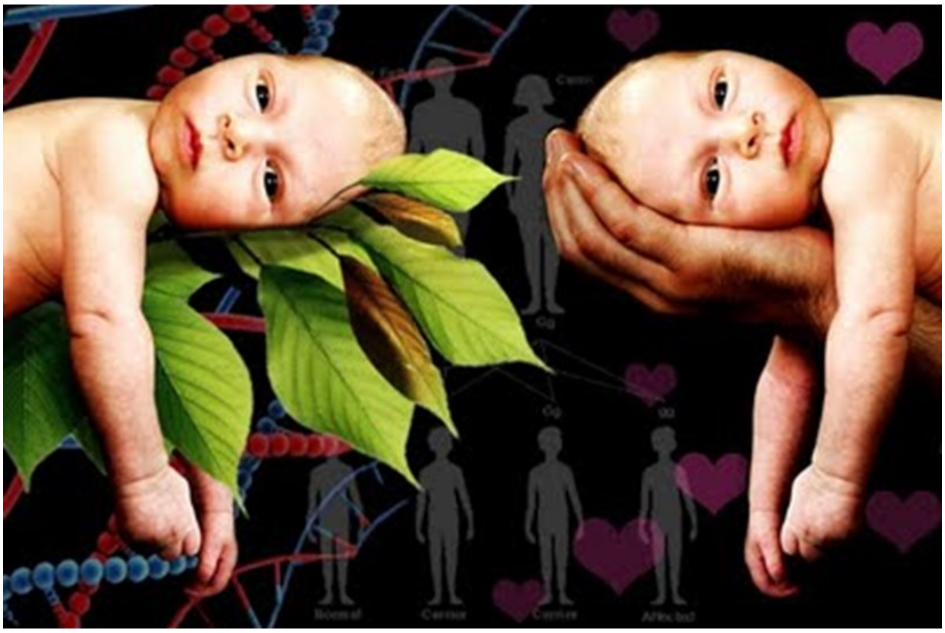

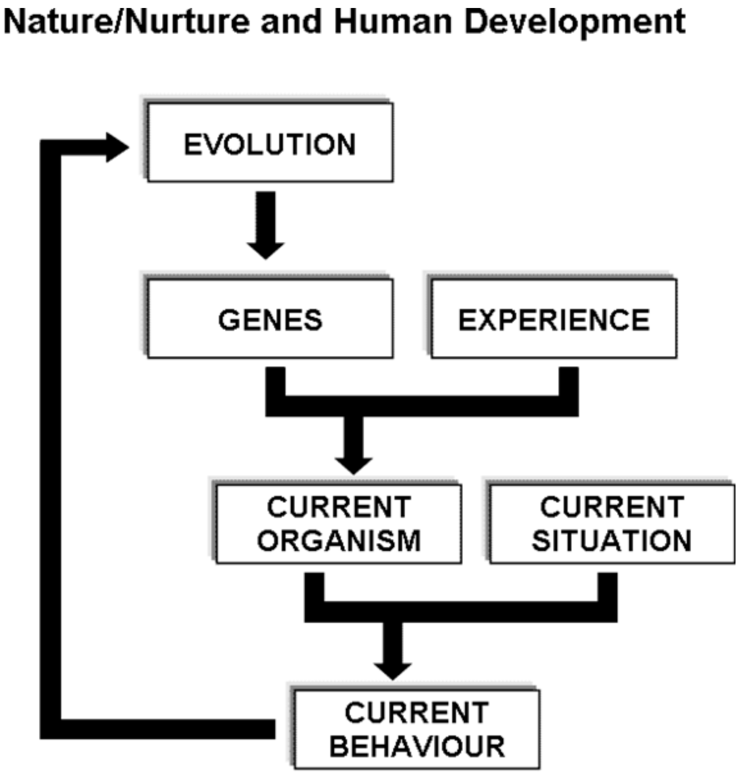

Source: Pinel, P.J. (2000). *Biopsychology* (4th Edition). Boston: Allyn and Bacon.

… with language and the meaning of words.

In a similar way, motor development can only happen when a child is placed in an environment where this is actively stimulated. Development is seen as a complex interplay where new skills (like grasping, reaching) are acquired when there is active interplay between the child, the environment and the circumstances of the tasks.

This dynamical system approach is the opposite of the "maturation theory" inspired by the evolution theory of Darwin.

Vision child development

Neural development of the child – the functional neuroanatomy of behavior

Over the last years, a lot of new insights have been gained into the brain's function due the availability of new methods. Clinical neuropsychology is a rapidly growing research are and focuses on the relation between behavior and the developing brain's function (Swaab).

The development and the growth of the central nervous system starts at conception and evolves throughout a long process that eventually results in the support of complex tasks in daily life. To cross a river by jumping from one stone to another, the brain rapidly needs to register and process multiple sources of information and evaluate these against acquired knowledge to plan and perform the required muscle strength – direction – foot position to reach the other side without difficulty.

We can distinguish 3 periods throughout the brain's development (Swaab):

<u>Prenatal:</u> when all brain structures are formed. Developmental processes are characterized by proliferation – migration and differentiation. Eventually, brain structures are developed that in turn go through periods of proliferation – migration and differentiation. Axons are formed during migration, and dendrites are formed after migration. This is a process of synaptogenesis (contacts between nerve cells) and the formation of receptors where signal transduction occurs.

<u>The period between birth and 4 years:</u> is characterized by an explosive growth. Within 4 years' time, the brains will be 4 times as big as at birth. Synapses that are formed are the so-called experience expectant-synapses. These synapses come from stimuli that are expected in a normal environment.

<u>The period between 4 years until young adulthood:</u> is characterized by refining of the structure. The development takes on a character of reorganization that is managed by genes and the environment. On the cellular level the experience expectant-synapses continue at a slower pace for some time. Meanwhile, already formed neural circuits are further expanded under the influence of stimuli from the environment. These are called the experience dependant-synapses and have the chance to form and refine throughout life. This gives us the opportunity to continue learning new things.

The period in which brain development takes place is considered sensitive. The period in which the most intensive brain development takes place is considered critical (period 1 and 2).

Child development within PEO
Person - Environment - Occupation

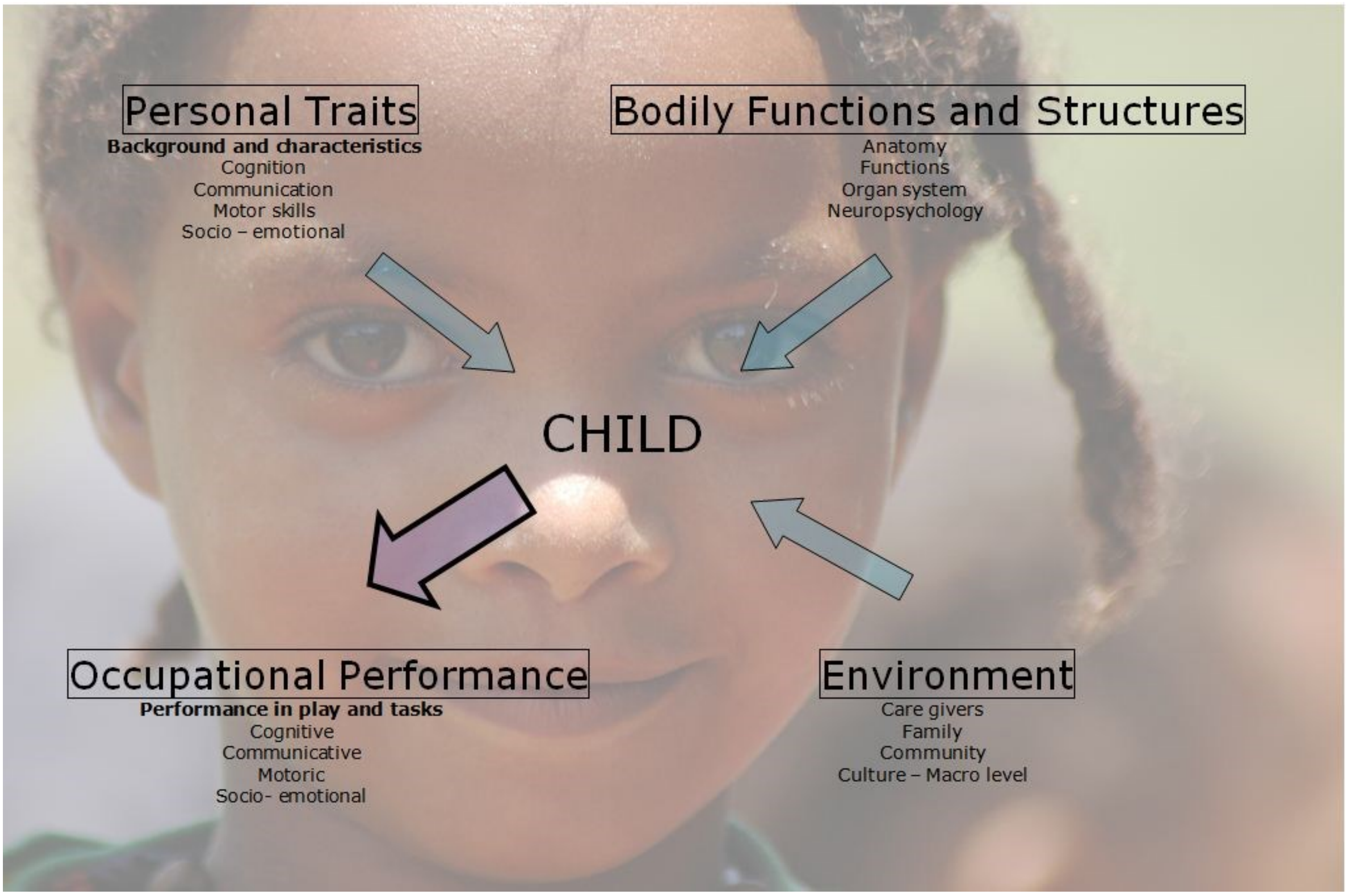

A child's behavior is determined by the interaction of the environment, the organism, personal traits and the circumstances of the tasks.

The environment contains the whole set of structures within the micro-, meso-, and macro-level. A child is born into a family with brothers and sisters and in a community of certain culture with its own laws and practices.

The organism contains the bodily functions and structures. More specifically, this refers to the neural, muscle, skeletal, cardiovascular and metabolic system. The body has to undergo ripening to perform actions.

The personal traits are all experiences that the child has went through. The child's history is brought forward in this. We can also show the child's development level here.

All of these lead to "occupational performance": the individual development level and the individual skills of the child.

Child development within PEO
Person - Environment - Occupation

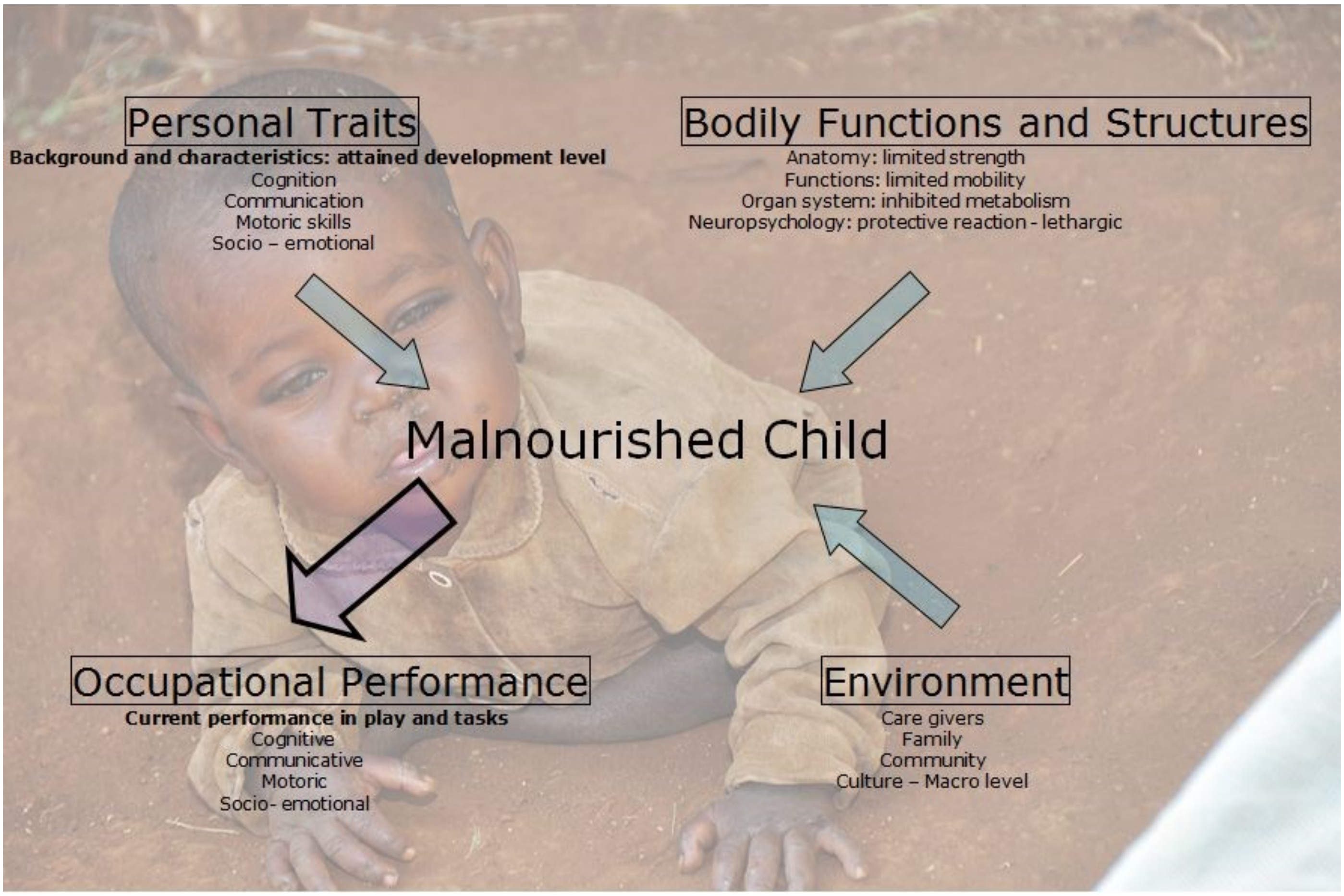

A malnourished child demonstrates specific behavior: he/she sleeps a lot, he/she cries very little, … They are usually seen as 'good' children that pose little problems.

The environment is focused on survival, i.e. the fulfillment of basic needs. Usually, the strongest children receive food first in their culture. Because malnourished children are hardly active, they usually stay at home in a less stimulating environment.

Bodily functions and structures: a malnourished child is weak, has limited strength and is lethargic. The body protects itself by expending as little as possible energy.

Within personal traits it is important to look at the past, the development level of the child during the period of malnourishment.

Developmental stimulation program

The basic principles of the developmental stimulation can be recognised in the keywords:

Stimulate - Enjoy - Safety

Stimulate: offering and reoffering opportunities of new experiences to the child taking in account its developmental level. Such training is not possible without knowledge of child development.

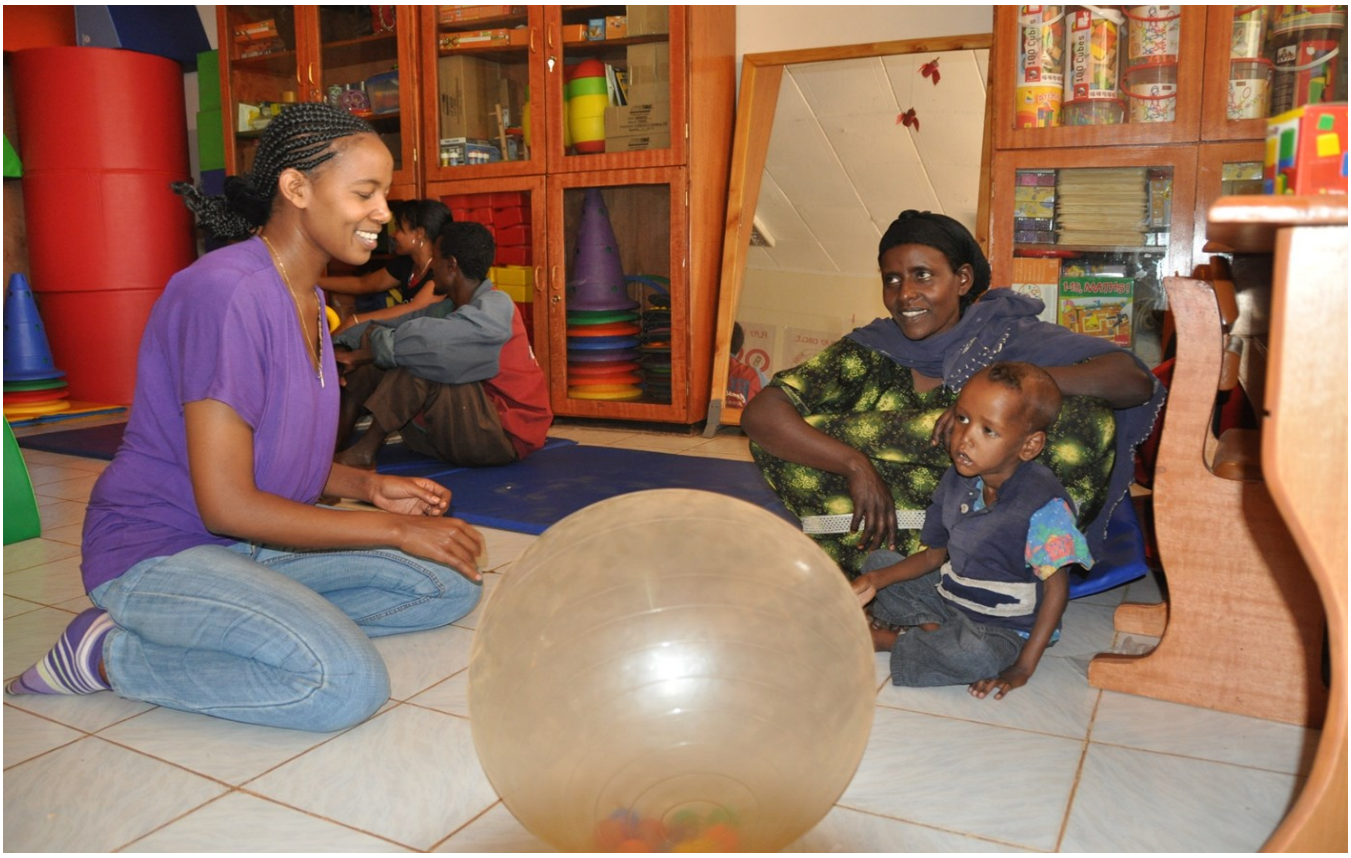

During such stimulation one of the caregivers (father/mother/…) is always present. The trainer explains to the father or mother which exercises will be done and why they are important.

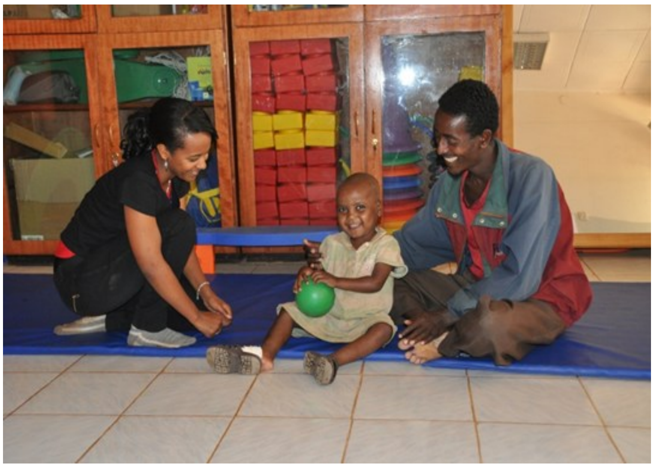

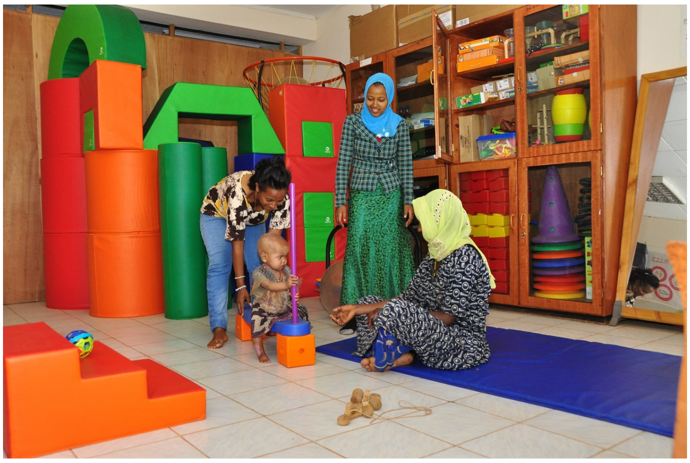

Developmental stimulation program

Stimulate - **Enjoy** - Safety

Enjoy: to be accessable for developmental stimulation, the child has to enjoy the new experiences. In this perspective the developmental stimulation is considered to be ‘learning by playing’. A child develops by playing. It is important that the person who facilitates playing also shows the child that she/he enjoys it. Such trainer will be labeled as a playleader. Play is every spontaneous activity of the child that she/he enjoys. After all, it is his natural environment (biotope).

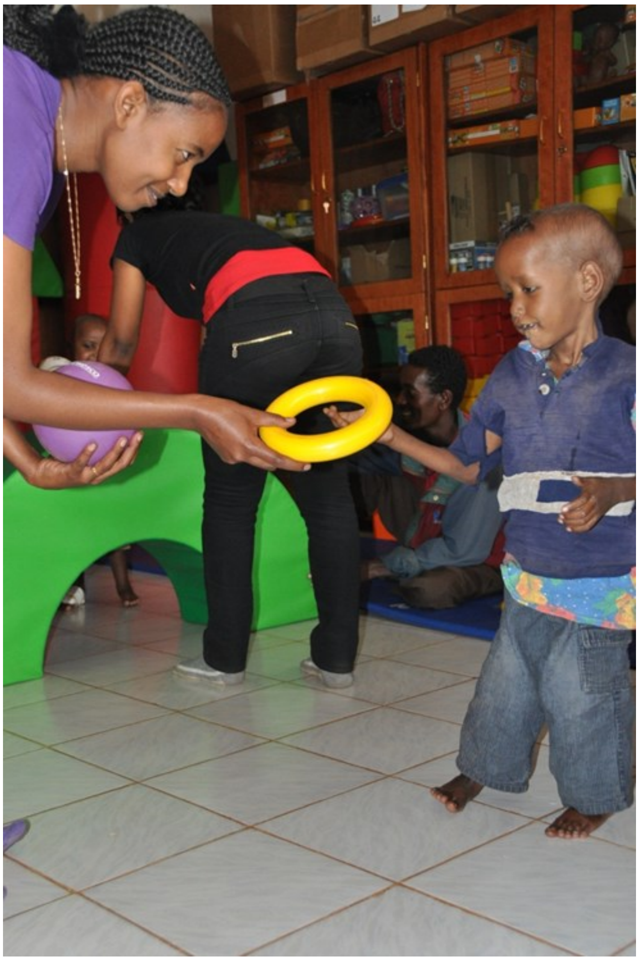

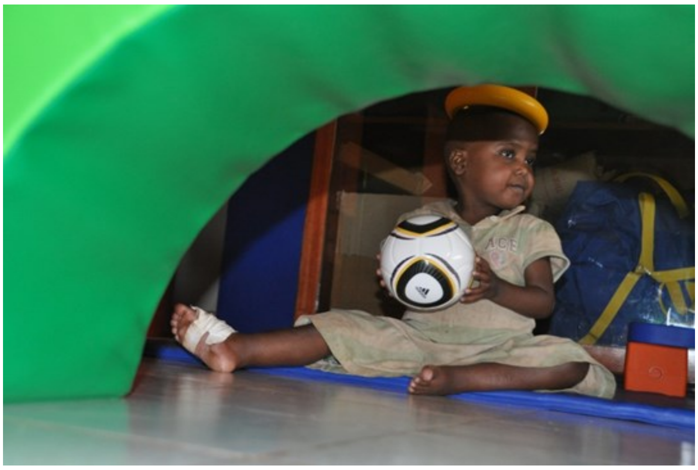

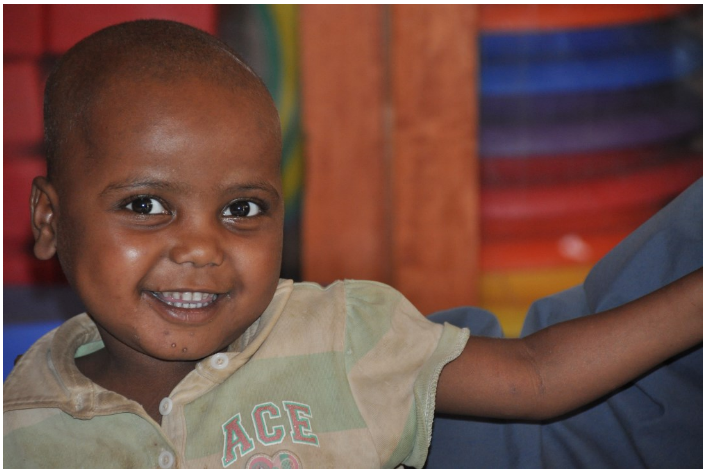

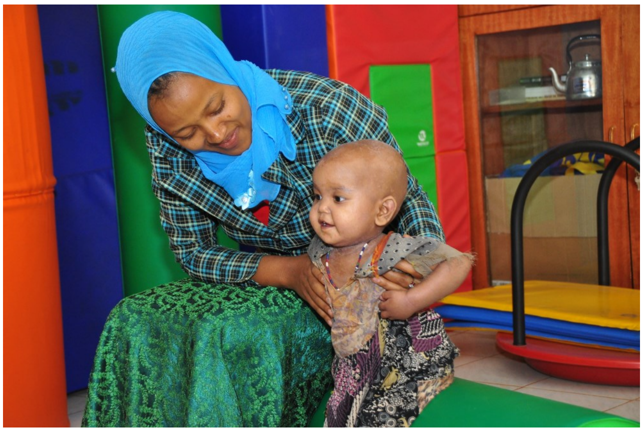

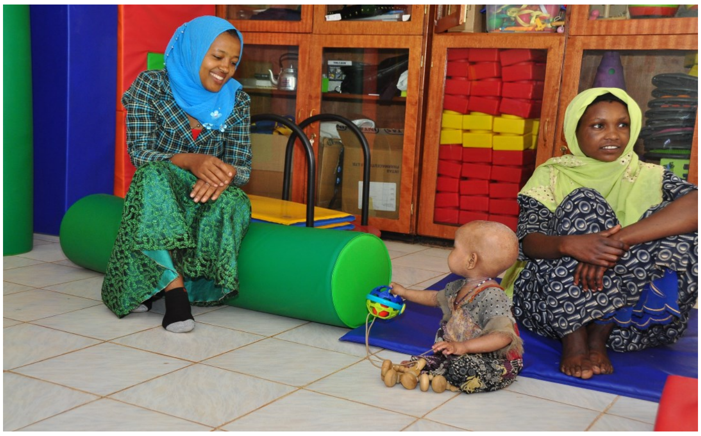

Stimulate - Enjoy - **Safety**

Safety: within each play situation it is necessary to estimate very well the strengths and weaknesses of the child. A child should feel safe. Basic trust is a must to trigger play. On the other hand, it is also important to take into account the vulnerability of a child. We let the child spontaneously explore without forcing him. The pictures show that little by little the child leaves his mother.

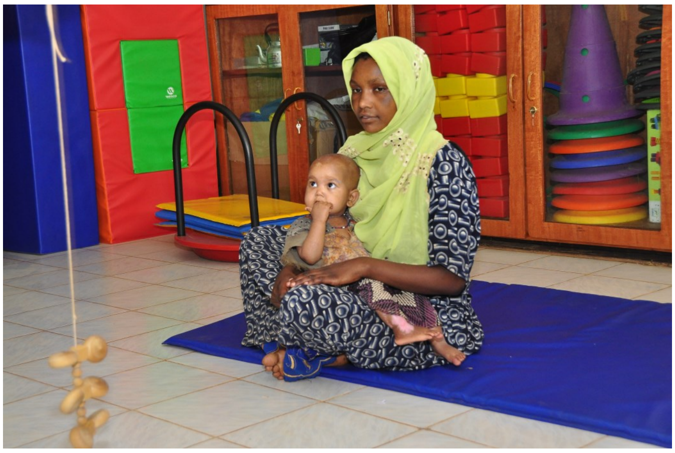

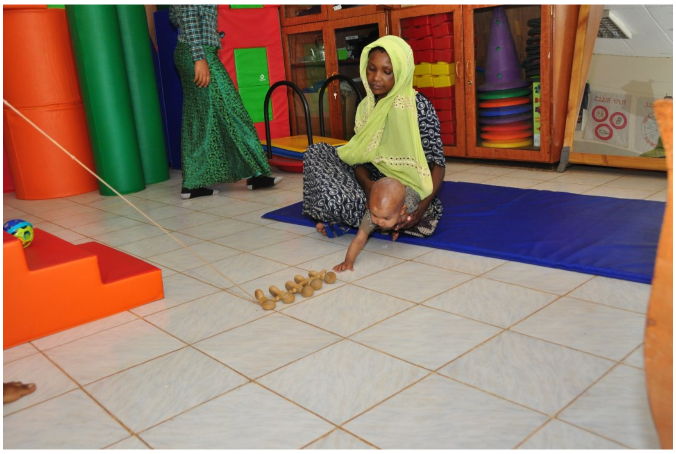

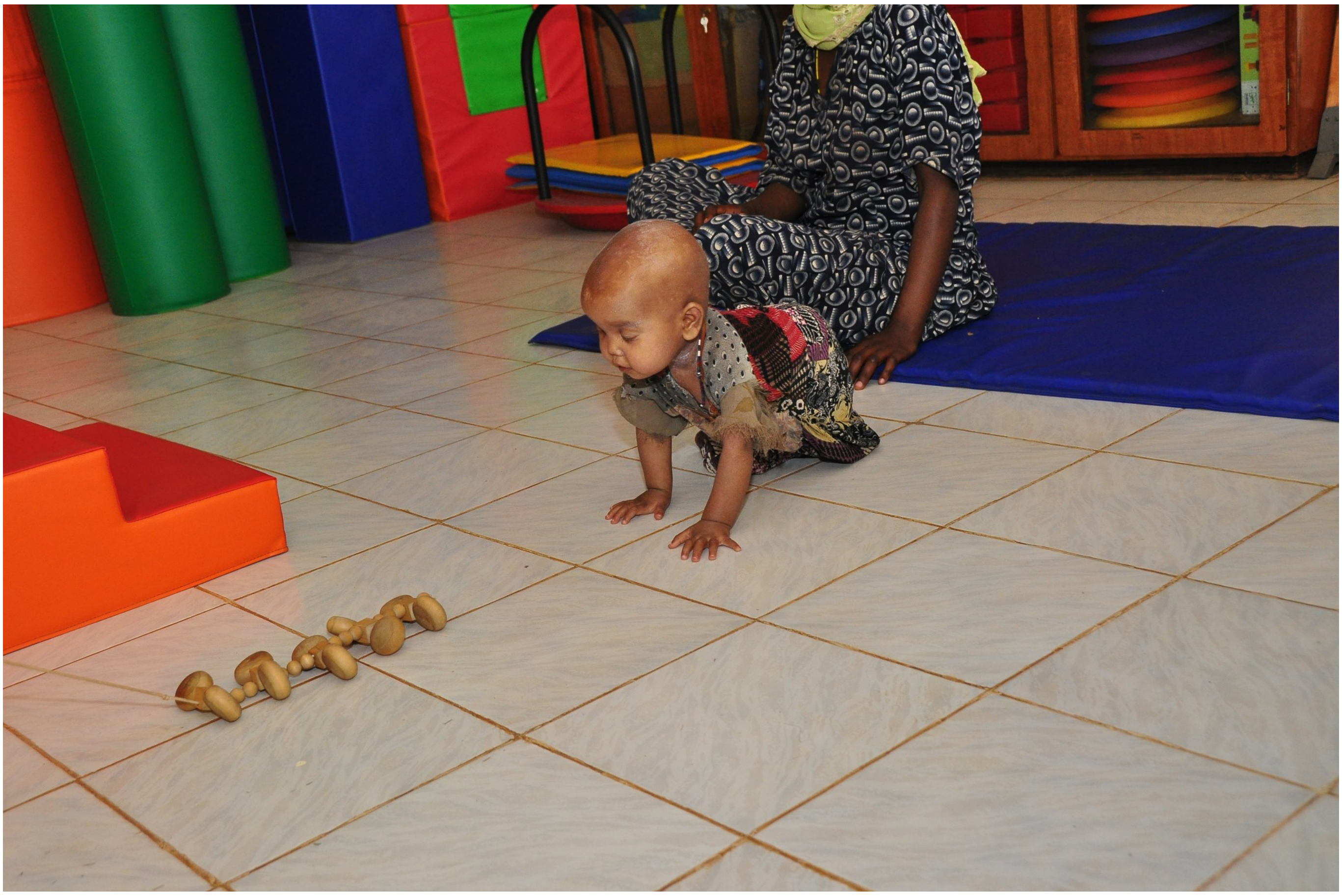

Operational design of SES in Jimma

The SES concept is designed in cooperation with the playleaders.

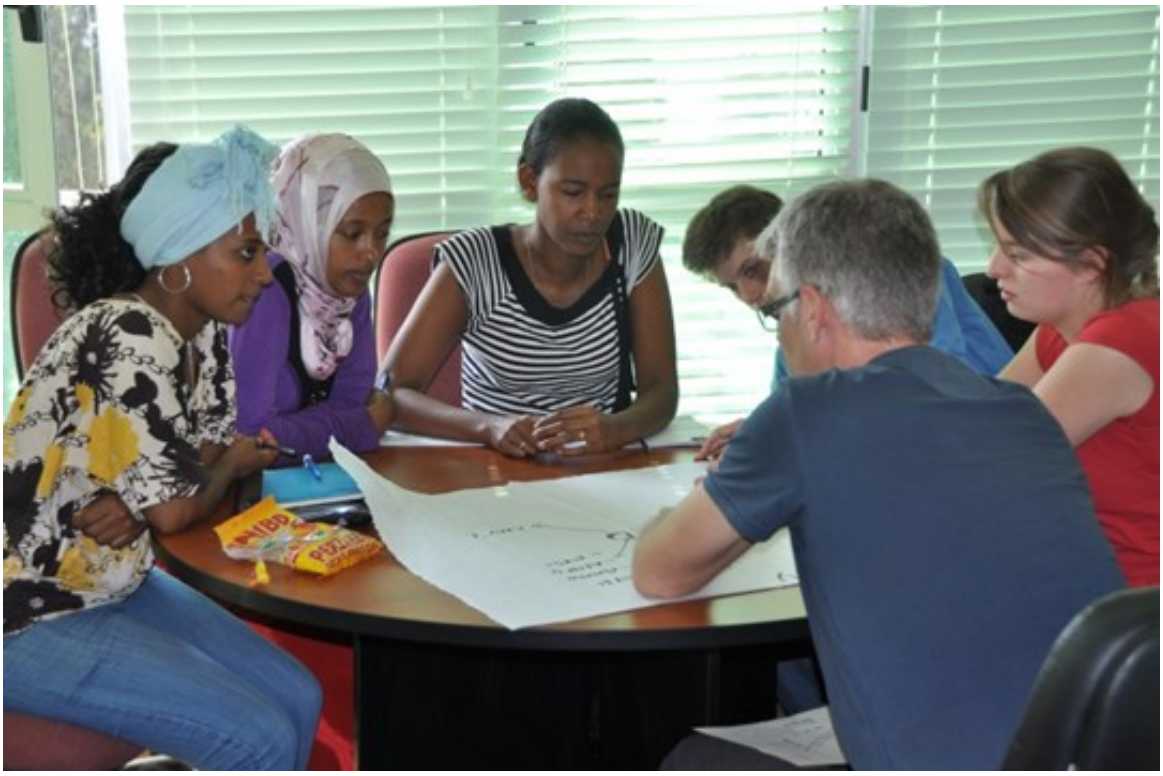

How to use cubes as basis for constructional games.

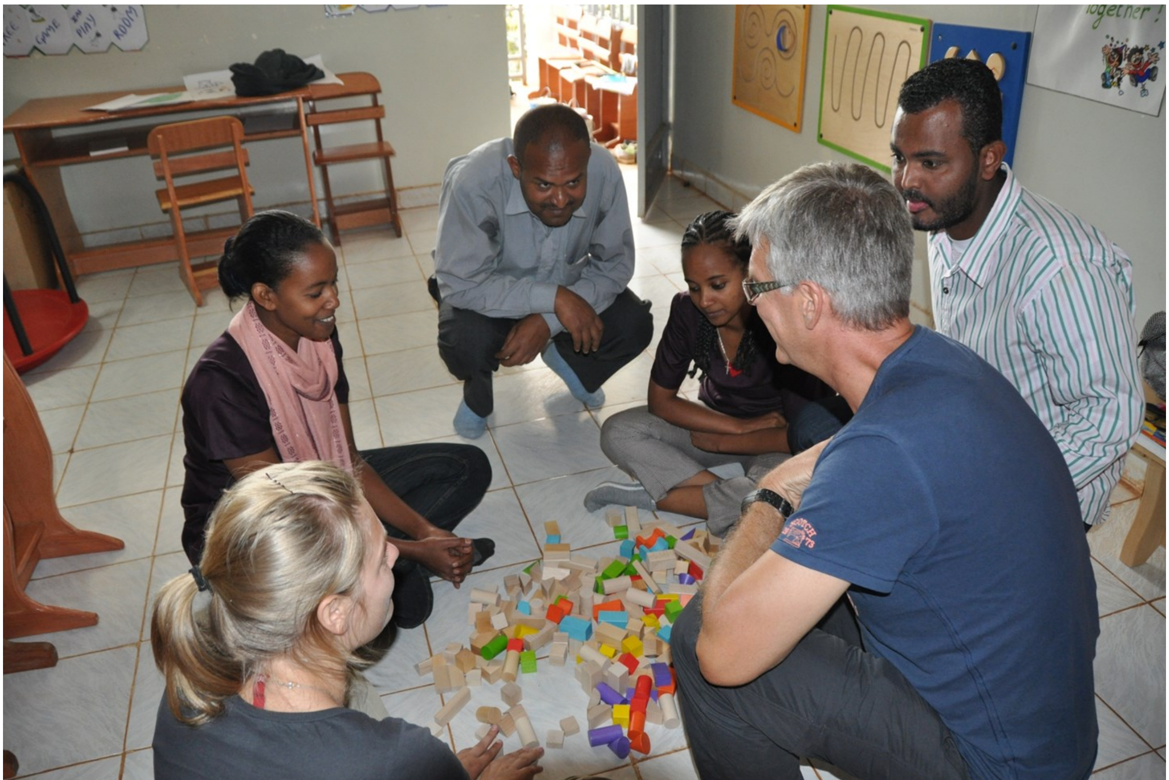

Operational design of SES in Jimma

Working together to compile a developmental rehabilitation program.

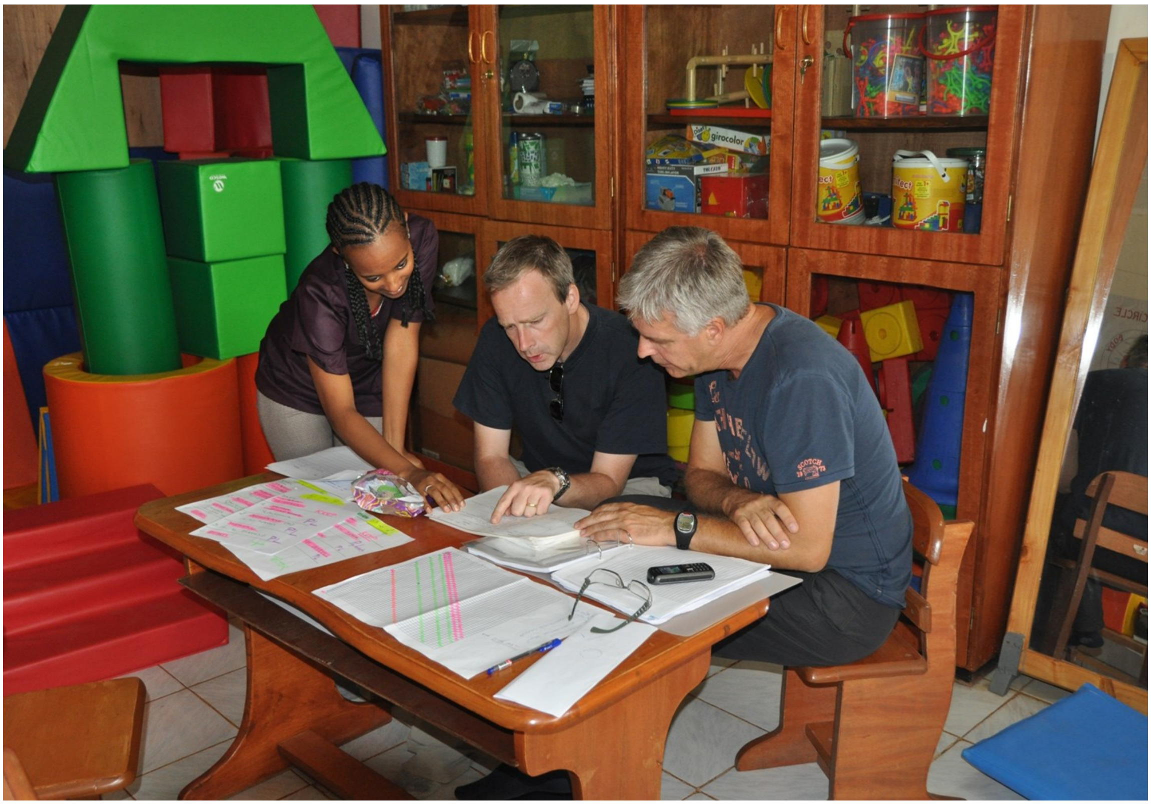

Lessons in the playroom.

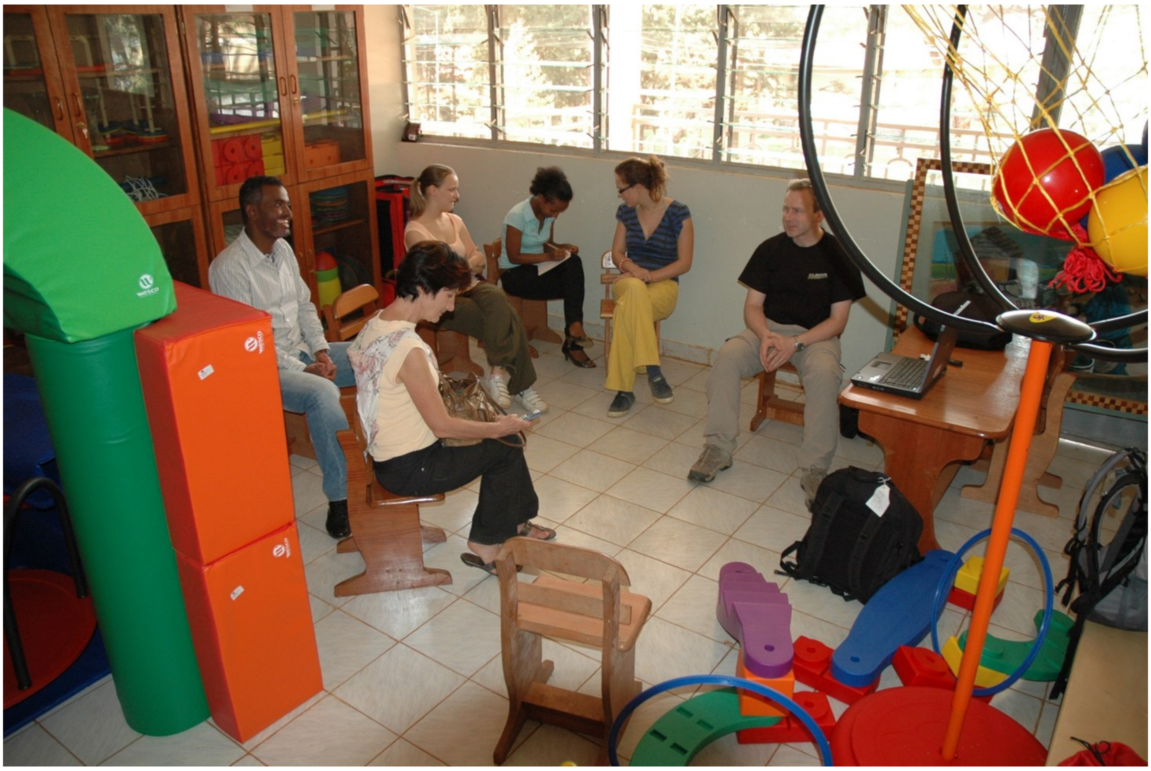

Practical design of SES in Jimma

Sharing experiences in relationships.

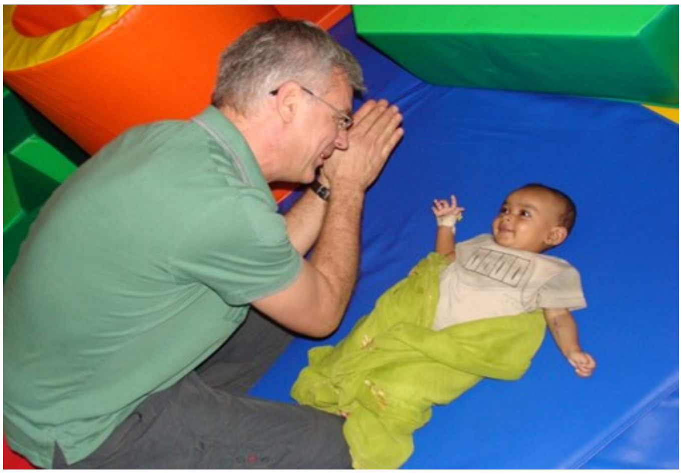

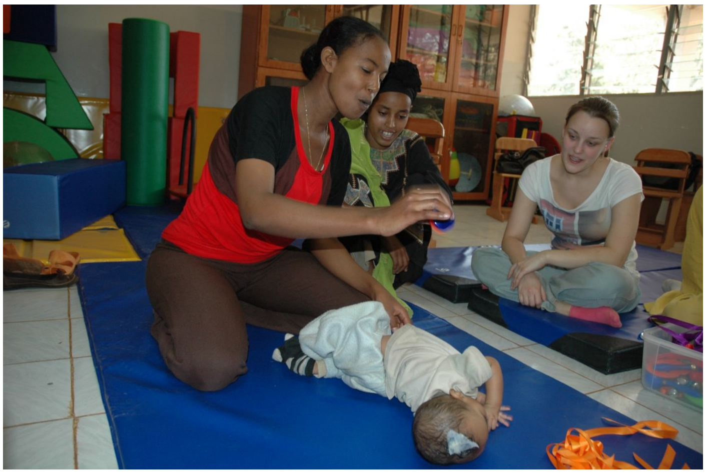

Showing how to catch the attention. The skills are first transferred to the playleaders but the main goal is teach the mother how to do it.

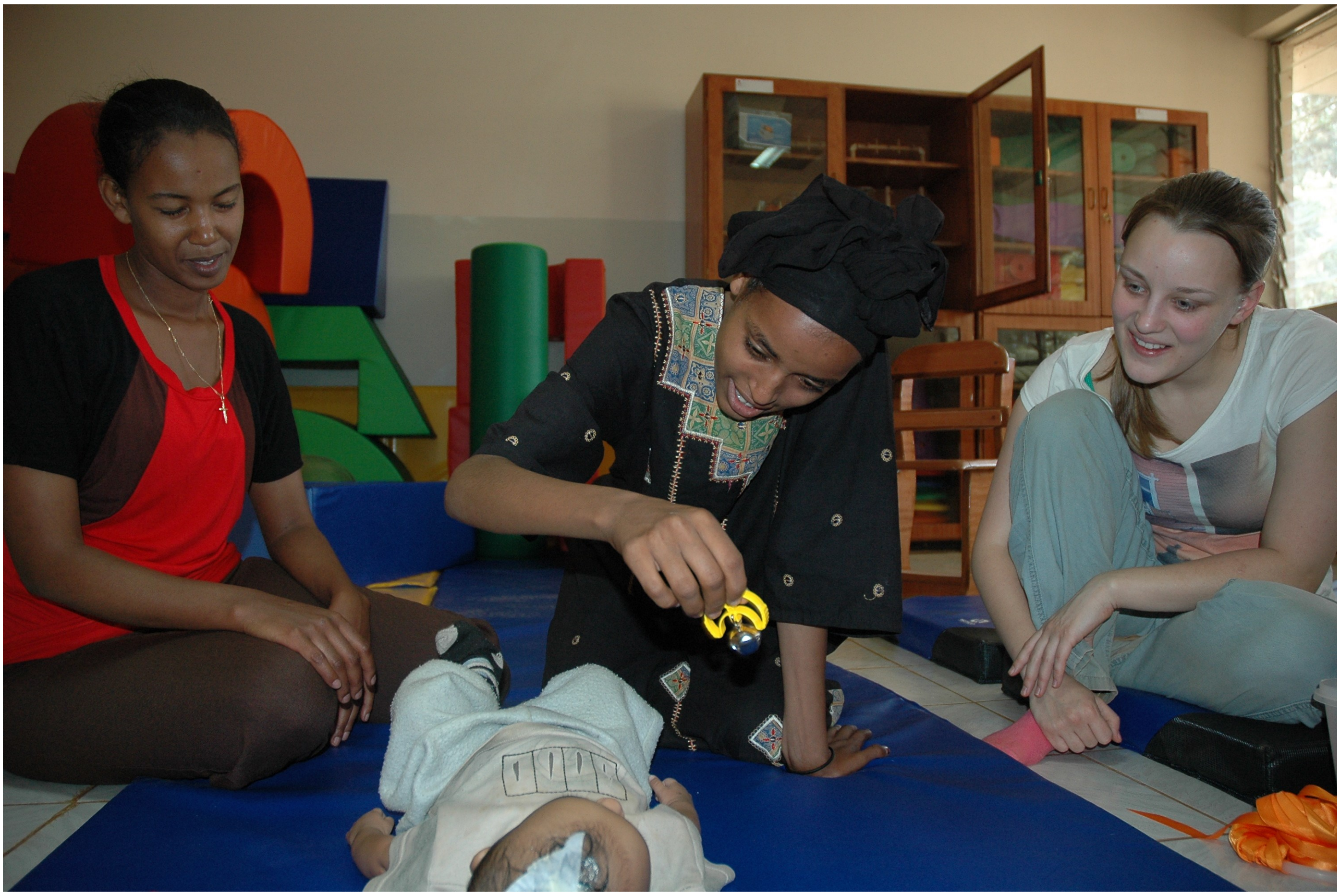

Practical design of SES in Jimma

Within the research project at Jimma University, 3 playleaders were recruited taking in account the following 3 important selection criteria: enough experience with small children, child-friendly attitude, willing to learn. These playleaders received a practice-oriented training with focus on the following aspects:

- Knowledge of play levels: knowledge of development in a child's play level, of the different components of development (motor - language - cognition - social skills)
- Knowledge of play and different ways of play
- Knowledge of play therapeutic guidelines:
    - Feuerstein: Mediational Intervention for Sensitizing Caregivers (MISC)
    - Movement Pedagogics of Sherborne
    - Small steps: Early Intervention Program Macquarie
    - Neuromotor Task training (NTT)
    - Sensory Integration Theory of J. Ayres (SIT)
- Importance to observe children

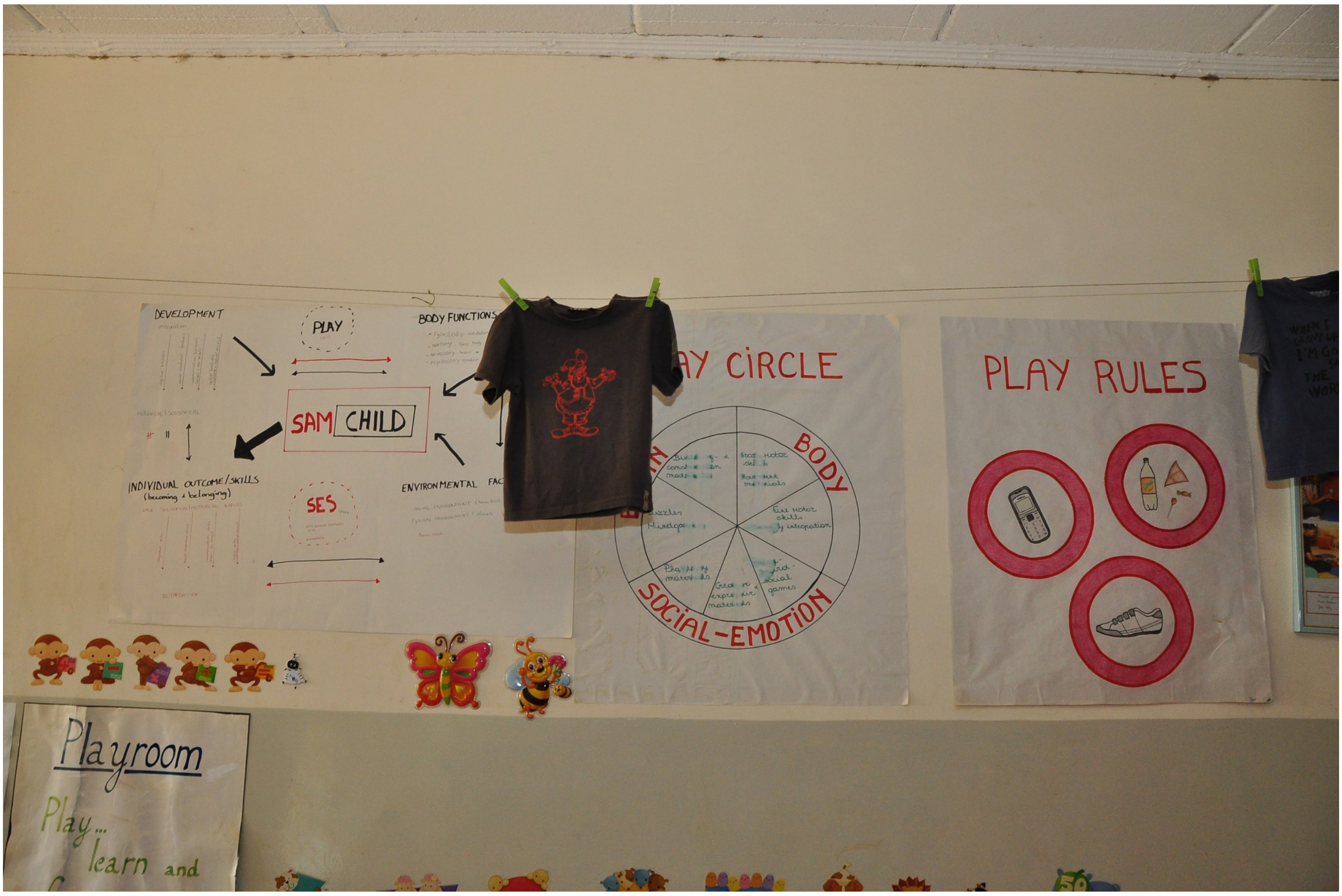

Certification for SES competencies

Evaluation and approval of SES competencies by providing a SES certificate.

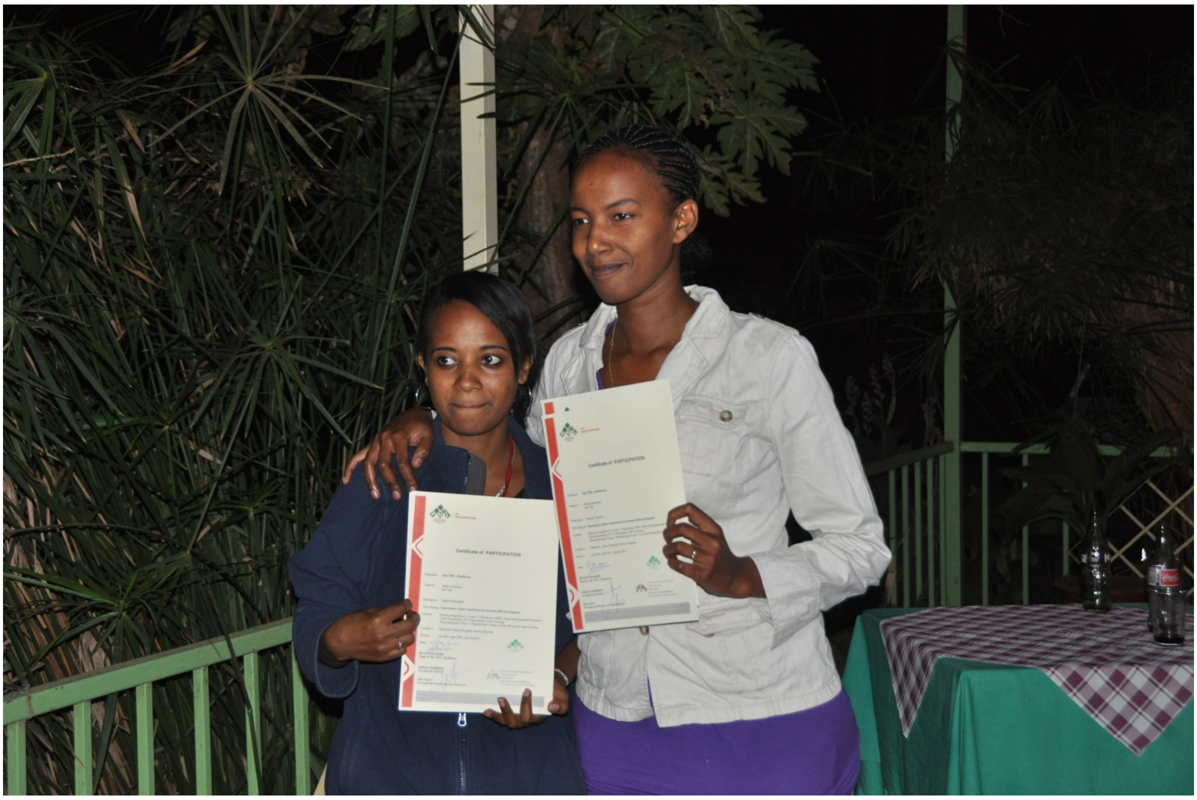

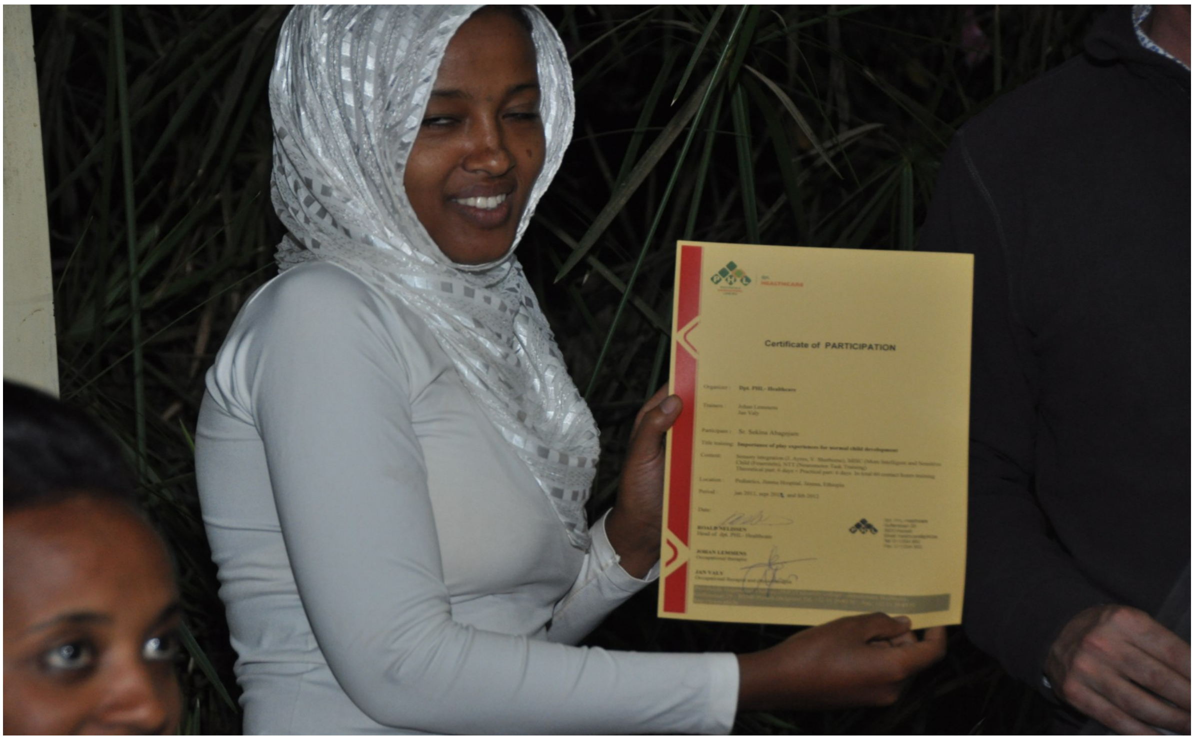

SES at Jimma hospital

At the intake of a severely malnourished child in the hospital priority will be given to specific treatments to stabilise the child. After medical approval by the physician the child will be refererred to the SES program. Before entering the program the child will be tested with the Denver II Jimma. The SES developmental stimulation program in the hospital consists of 8 individual sessions and 8 sessions in group.

Training of individual skills of the child within a separate room (playroom) in the pediatric unit in the presence of the caregiver

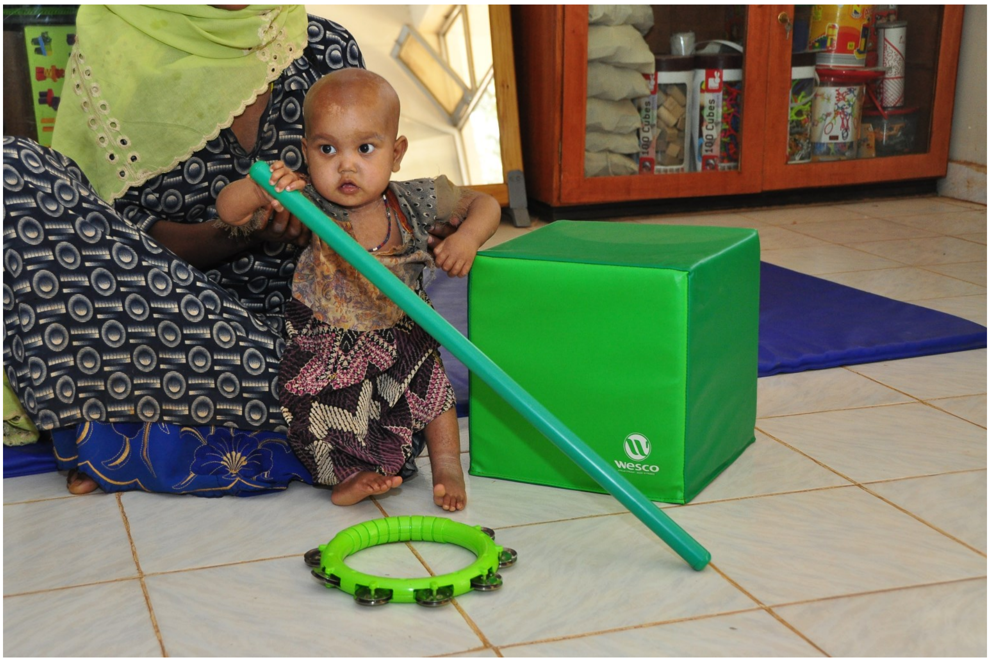

Practising social skills in group on a playground in the presence of the caregiver.

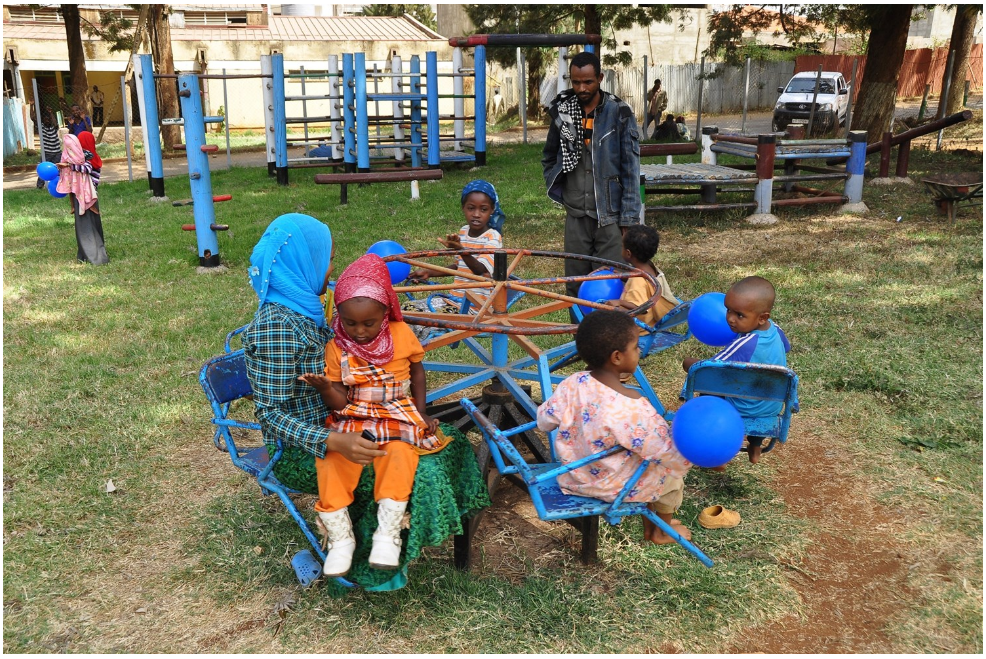

Application of the SES in Jimma

At discharge, the children will be tested again with the Denver II Jimma and they receive a basic kit to practise at home.

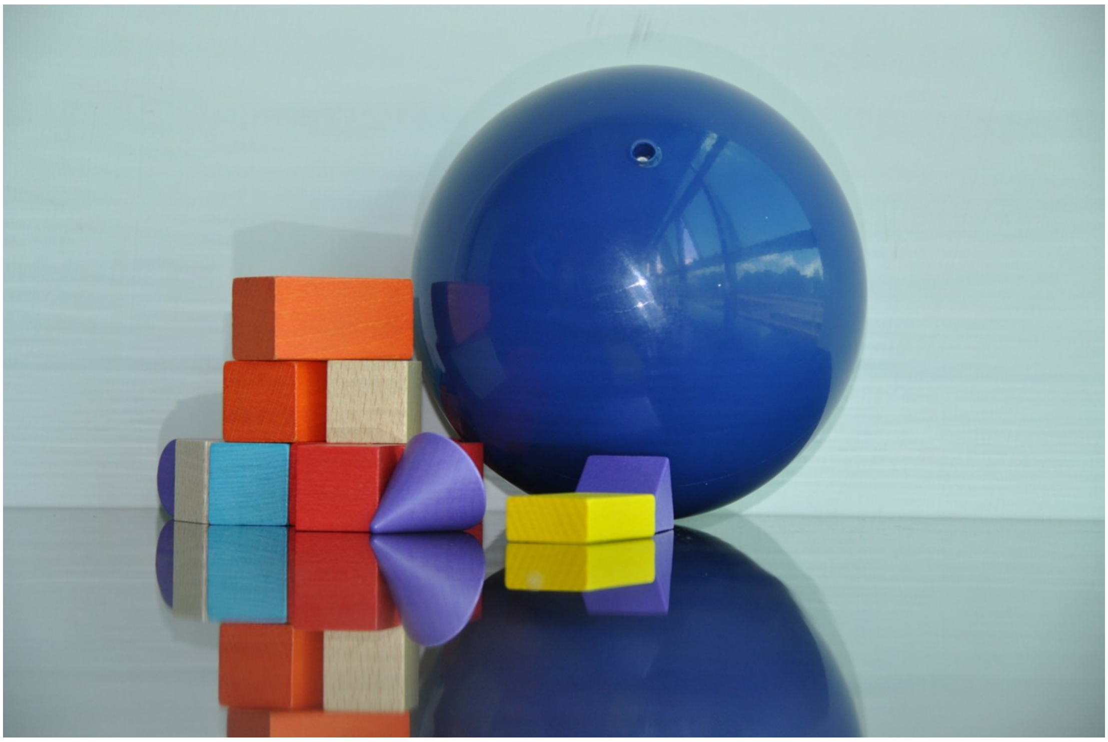

After 3-6-12 weeks, playleaders plan a visit to the child's home to follow up on the child and to provide new play material, specifically adjusted to the developmental level of the child.

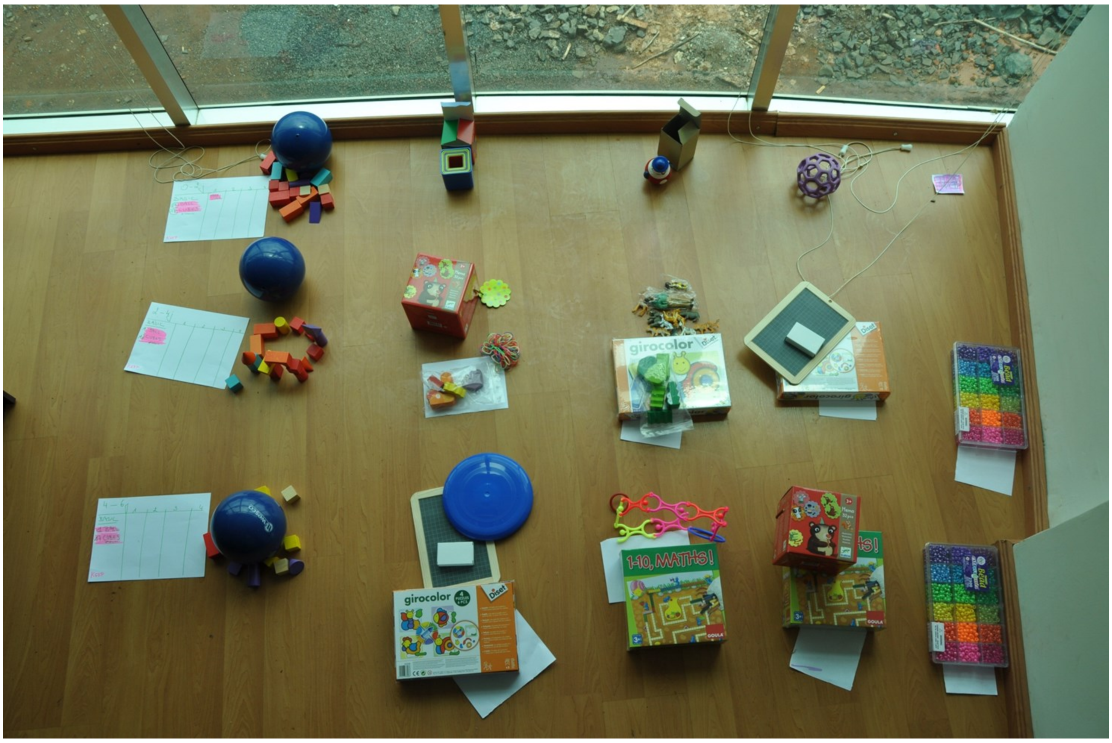

SES during home visits

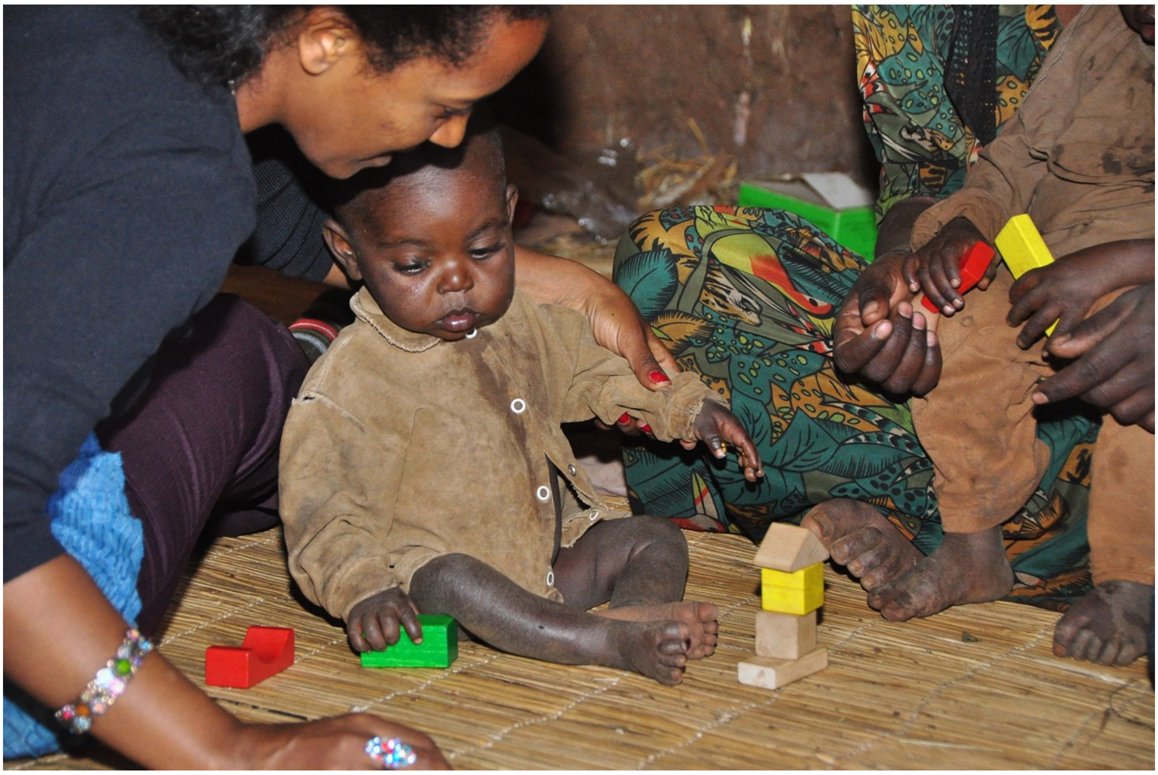

The playleader is guiding the child to play with cubes, making a tower.

The father shows his son how to write on the blackboard, he is at the same eyelevel.

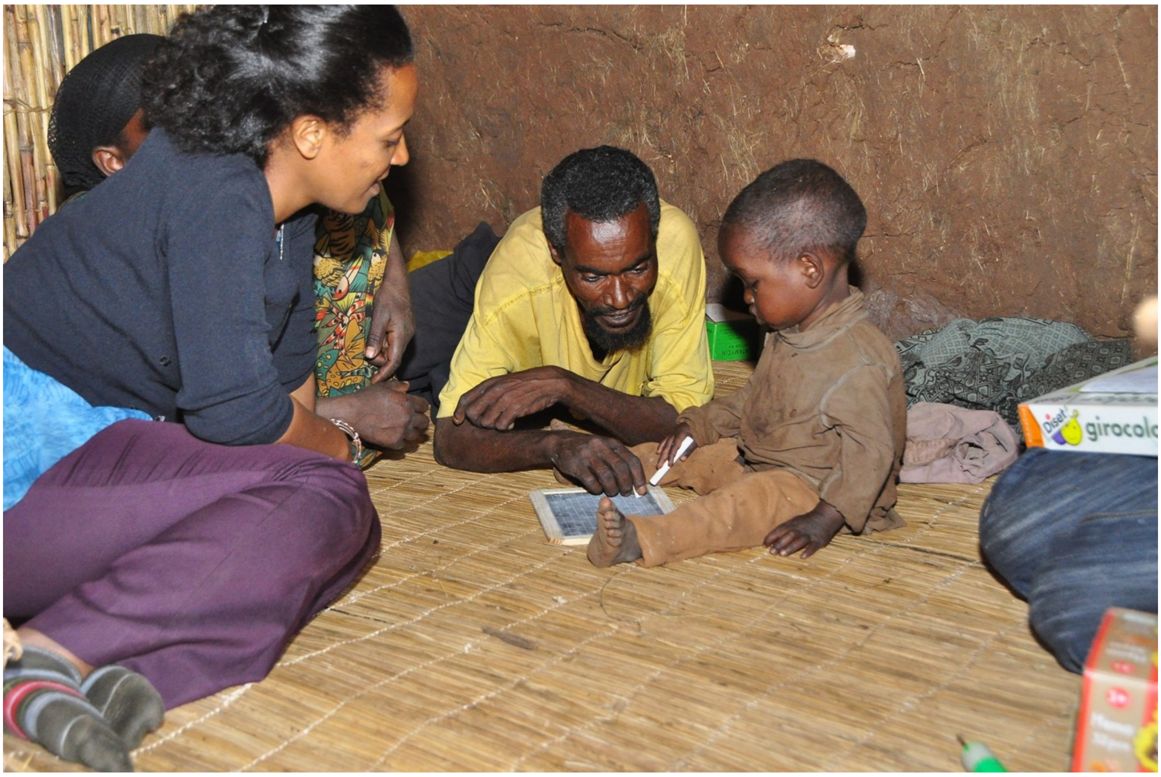

SES during home visits

Community based playing: a girl from next door is playing together with the child while the playleader guides him.

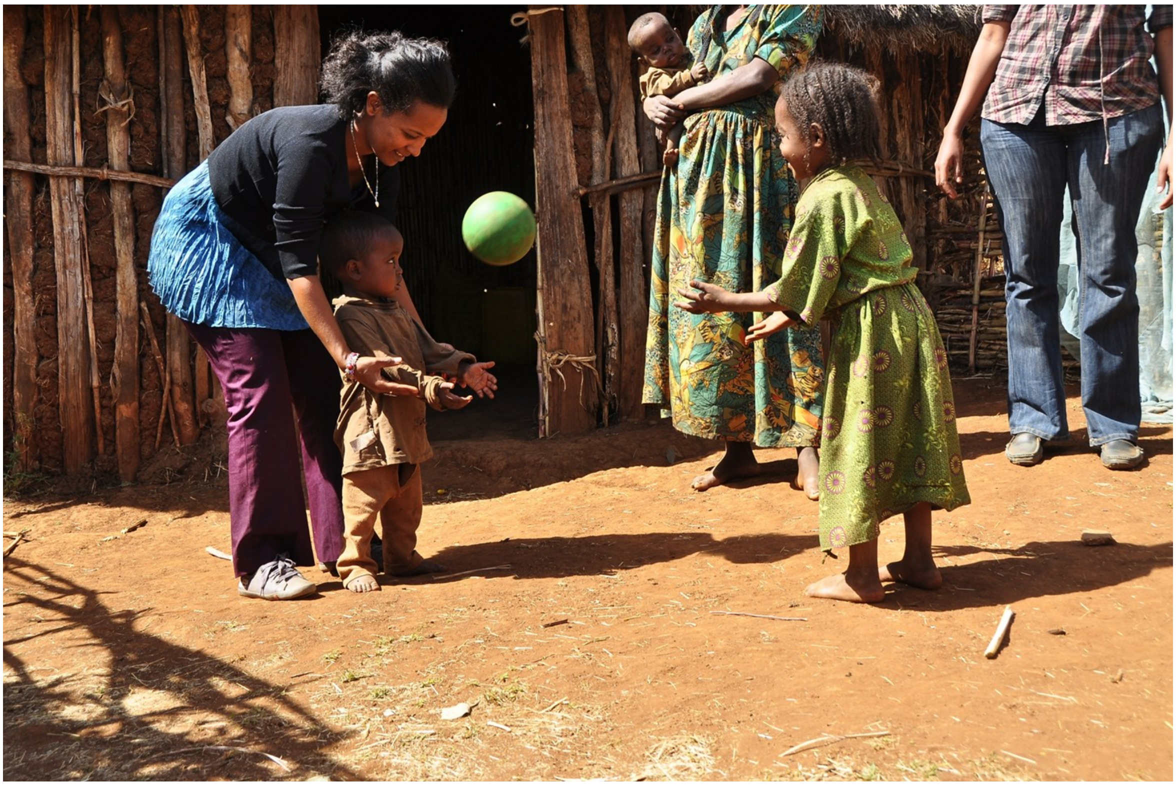

The child is exploring new playtools.

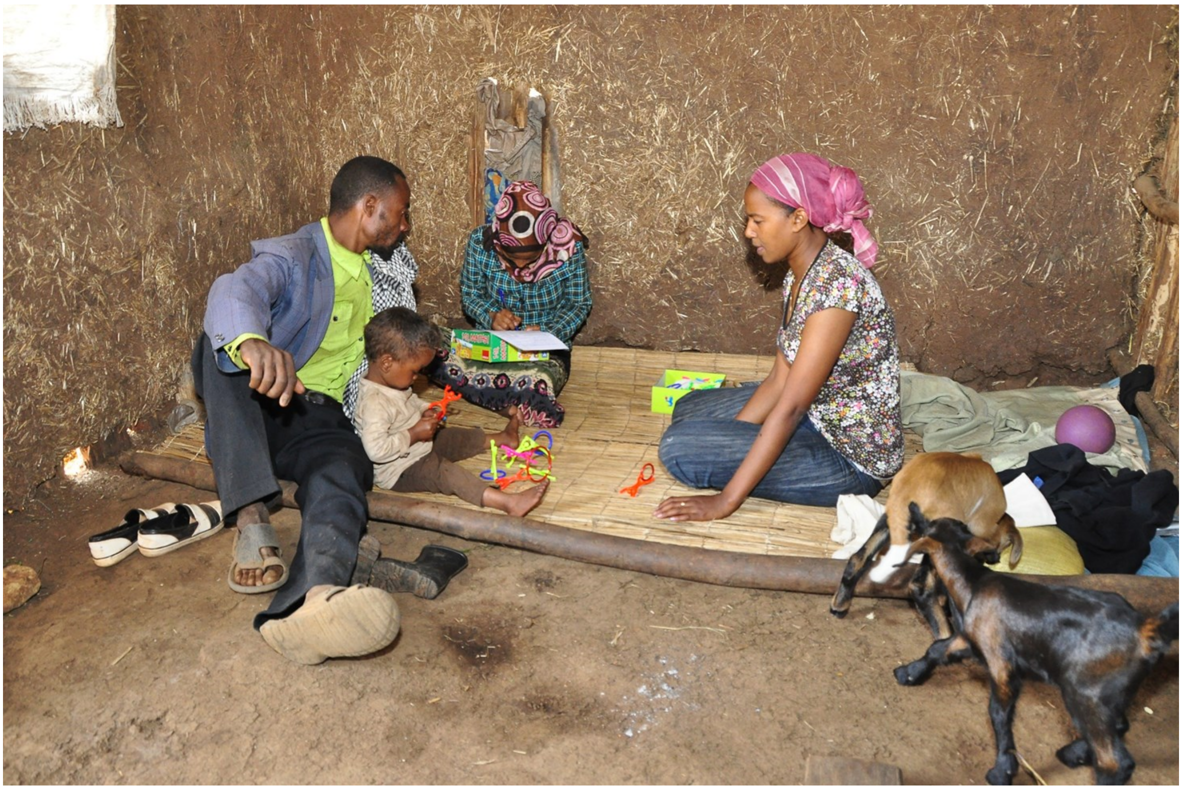

SES during home visits

Child and playleader working together.

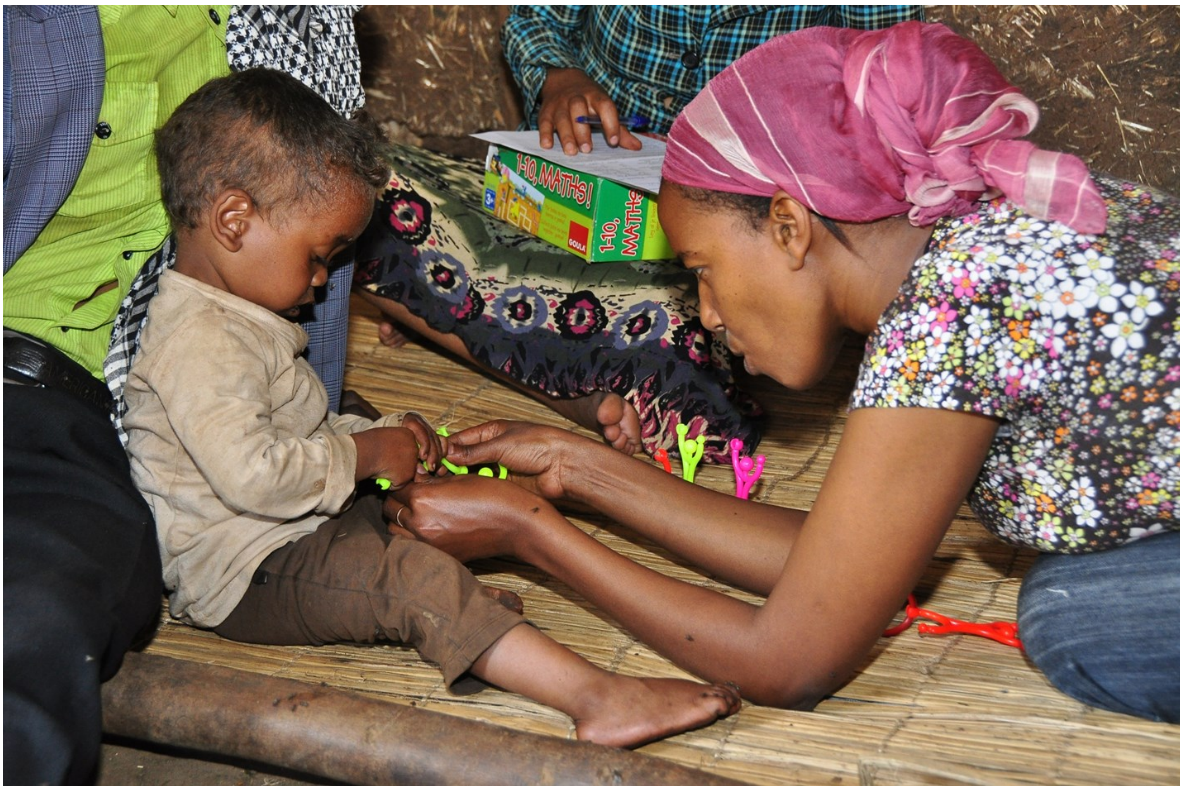

The father is involved in the fantasy game, making a kings crown.

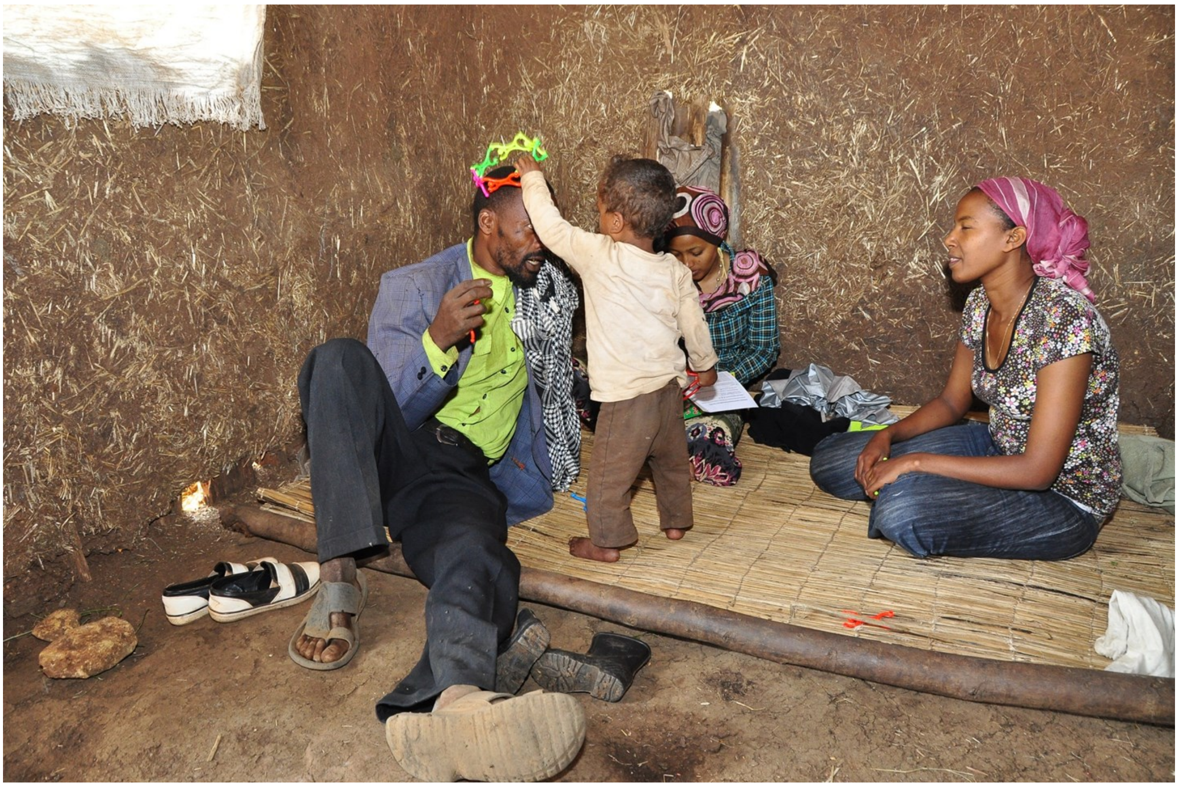

SES during home visits

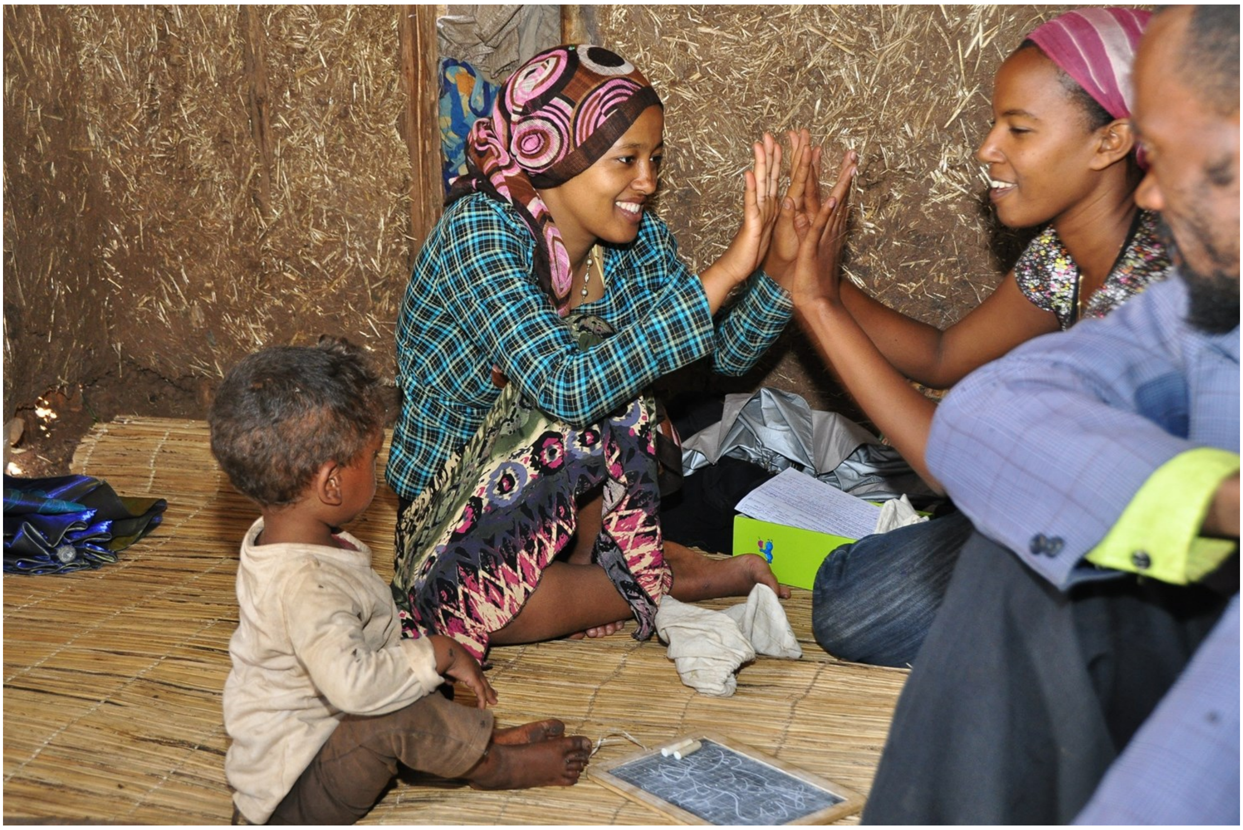

Playing without tools, using own body. Clapping hands while singing

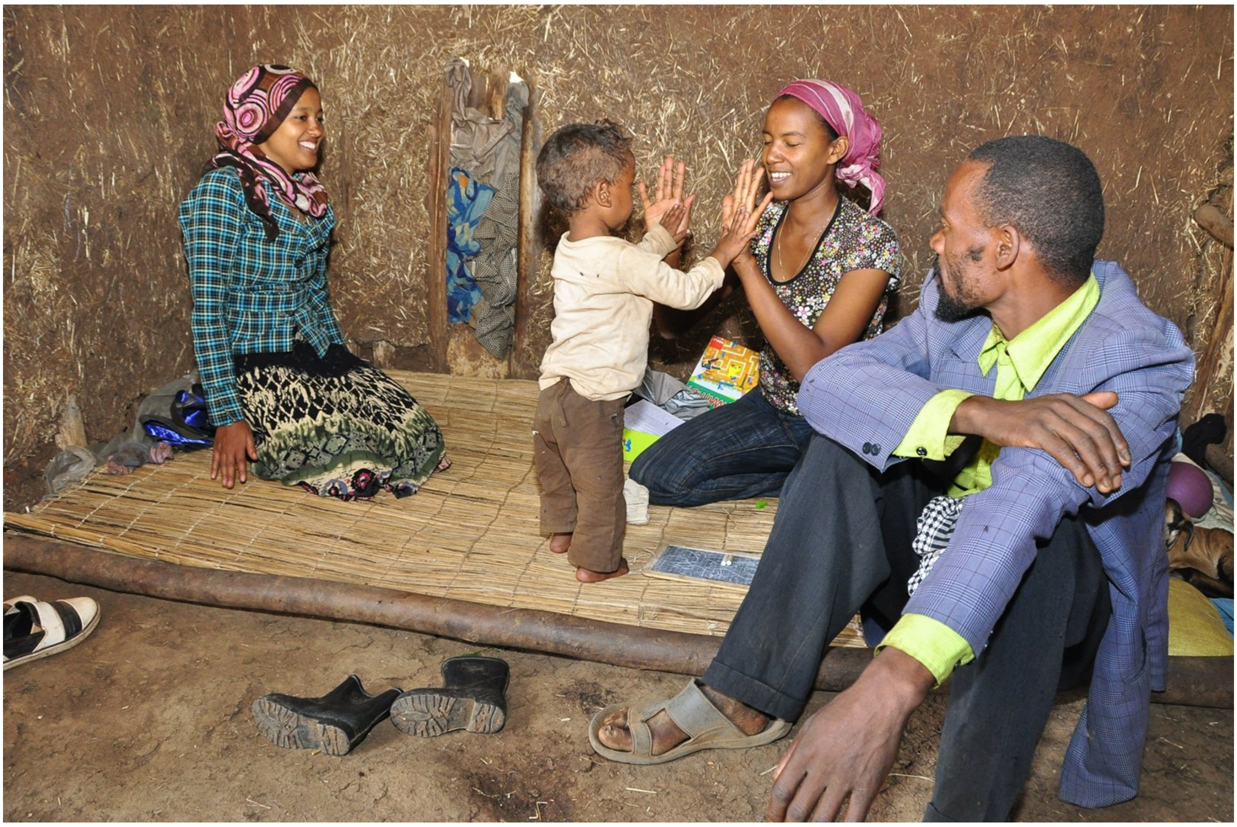

Developmental testing after 24 weeks

After 24 weeks the child is tested at home by the testers using the Denver II Jimma.

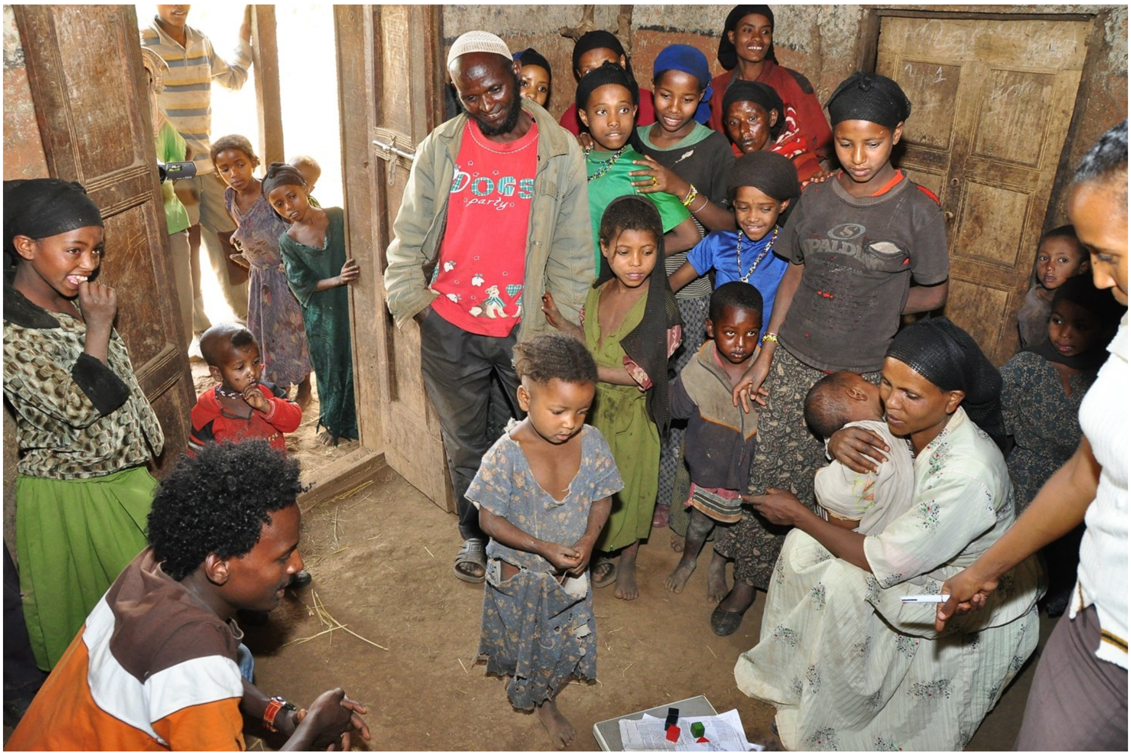

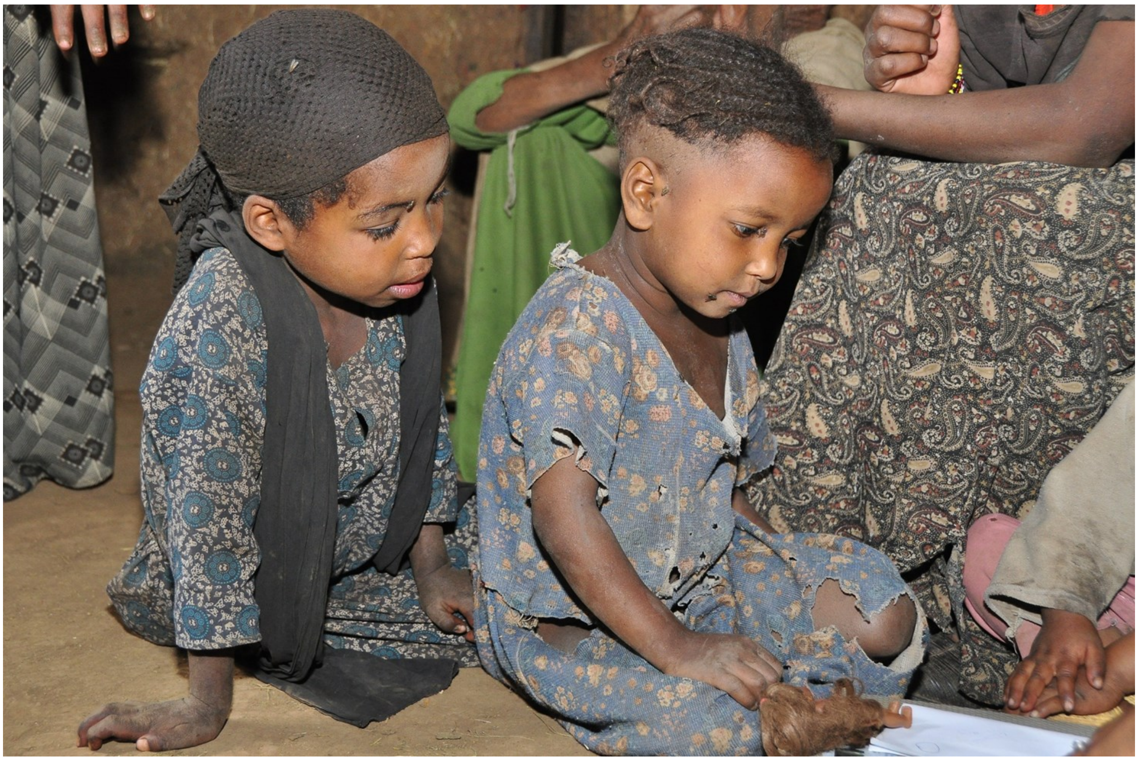

Measuring growth after 24 weeks

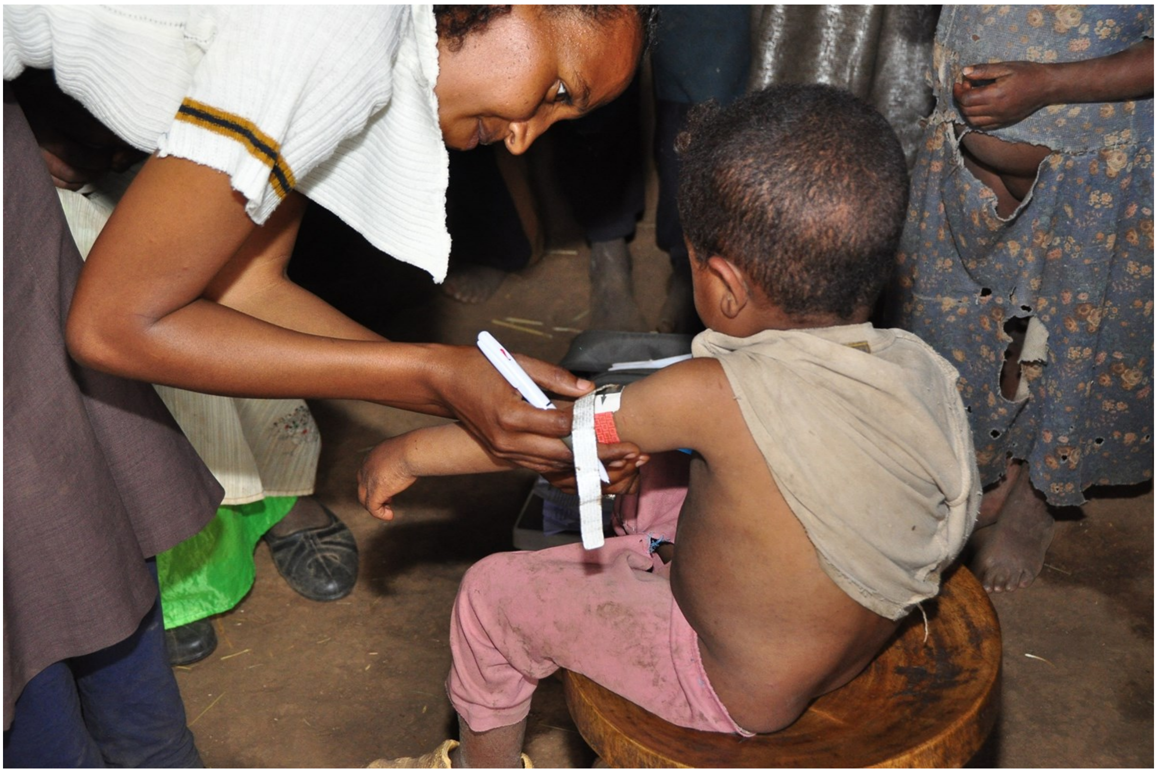

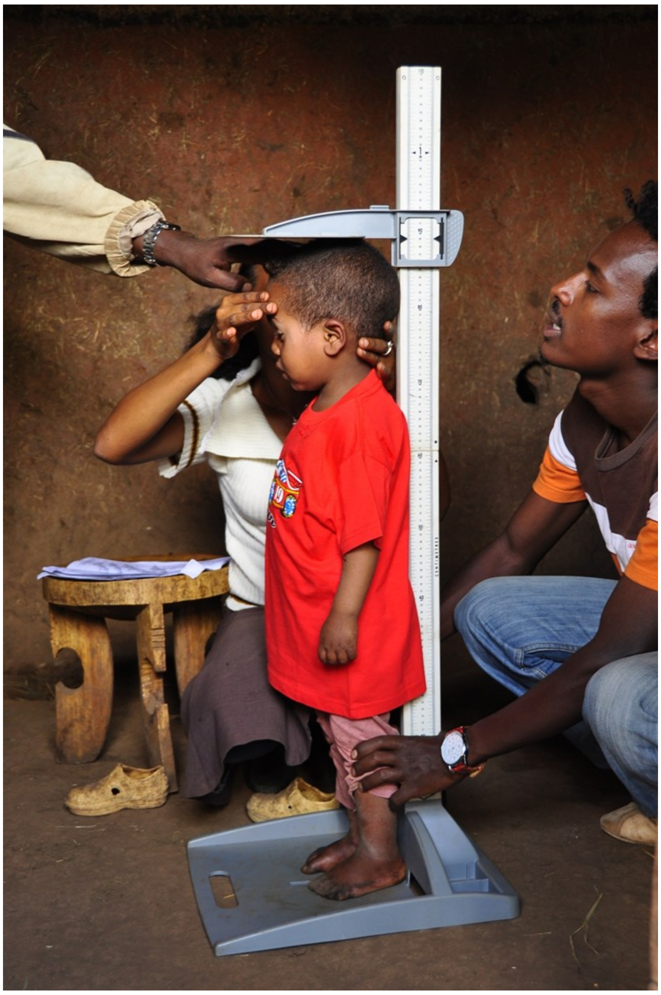

Biometric measurements

Sharing minds, changing lives

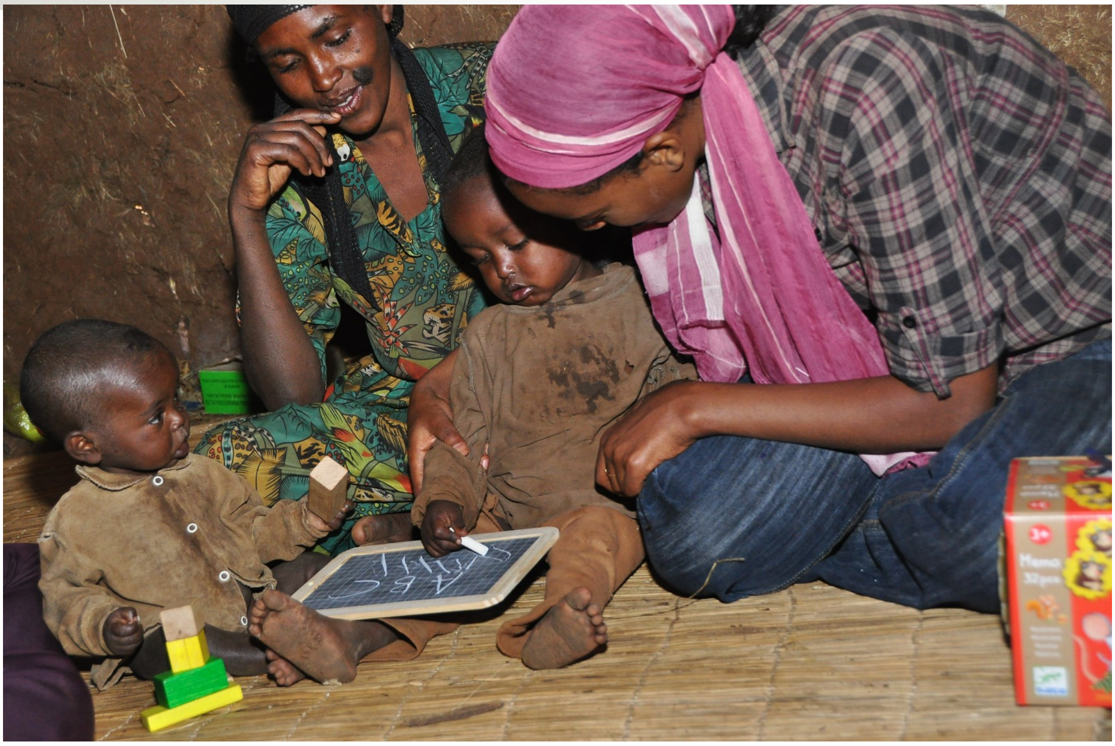

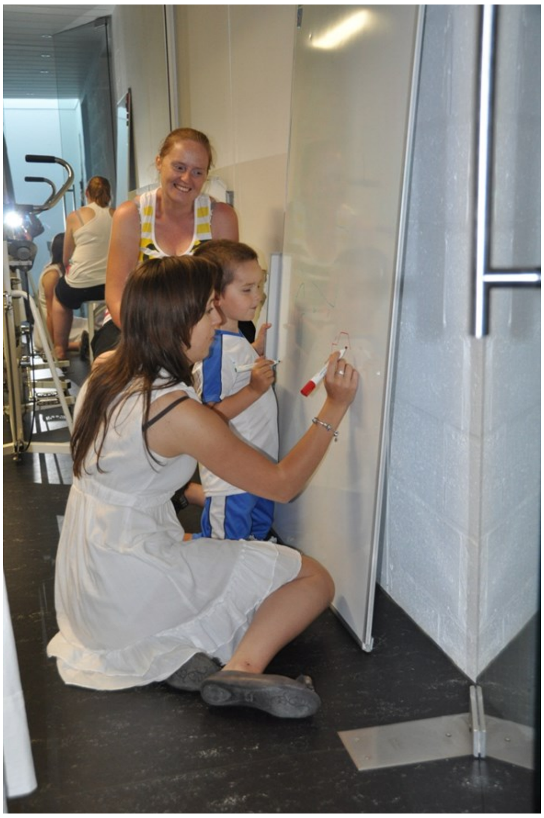

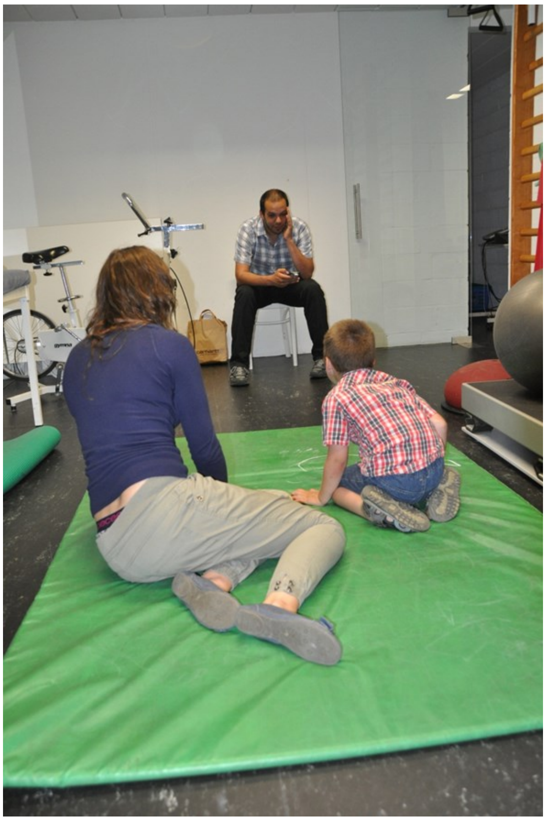

The exchange of ideas results in changing minds. Also in Belgium, parents become more involved in developmental stimulation therapy for their children.

Expertises of the team

Our team is multidisciplinary and multicultural.

The team consists of therapists with longstanding practical experience in treating children with developmental problems. They designed the SES developmental stimulation and apply it every day in their own practice. Many members are scientific researchers who test and evaluate the benefit of the interventions on the child's recovery. All of them are linked to a teaching institution (university college or university) or research institution. Multidisciplinary and multicultural cooperation makes the team strong in designing rehabilitation programs and scientific research in developmental problems of children in low– and middle -income countries.

The team is currently active

- in theoretical and practical training of nurses at Jimma University hospital
    - in assessment of child development (healthy/malnourished)
    - in developmental SES stimulation of malnourished children (and other children at developmental risk)
- in conducting scientific research.

The team offers

- support of the process of culture-specific assessment of overall development and growth of malnourished children in the hospital and on the field
- support of all activities which concern developmental stimulation of the child

Team members

Patrick Kolsteren (Prof, PhD paediatrician, UGent and ITM, Belgium)

Mekitie Wondafrash (MD, Jimma University, Ethiopia)

Marita Granitzer (PhD neurophysiologist, PHL Healthcare and UHasselt, Belgium)

Teklu Gemechu (Drs. psychologist with special educational needs, Jimma University, Ethiopia)

Berhanu Nigussie (Drs. psychologist, Jimma University, Ethiopia)

Johan Lemmens (OT, PHL Healthcare, Belgium)

Jan Valy (Pt, OT, PHL Healthcare, Belgium)

Kimberley Bouckaert (MSc Bioscience engineering, UGent, Belgium)

Tsinuel Girma (Drs. Pediatrician, Jimma University hospital, Ethiopia)

Sakina - Tigist - Amele (Playleaders, nurse, Jimma University hospital, Ethiopia)

Masresha - Aynaylem (Testers, nurses, Jimma University hospital, Ethiopia)

Literature

Swaab H., Bouma A., Hendriksen J., König C. (2011) Klinische kinderneuropsychologie, Uitgeverij Boom, Amsterdam

Mönks F., Knoers A., (2009)Ontwikkelingspsychologie. Inleiding tot de verschillende deelgebieden. Koninklijke Van Gorcum, Assen.

Prins P., Braet C., (2008) Handboek klinische ontwikkelingspsychologie. Bohn Stafleu van Loghum, Houten

Njiokikitjien C. (2004). Gedragsneurologie van het kind. Uitgeverij Suyi, Amasterdam

Bakker J., (2007), Gedragsneurologie voor paramedici. De Tijdstroom, Utrecht

Firth C. (2007) Nederlandse vertaling door Nancy Seghers (2011) Ons Brein. Hoe de hersenen ons verstand te boven gaan.EPO VZW, Berchem.

Swaab D. (2011) Wij zijn ons brein. Van baarmoeder tot Alzheimer. Uitgeverij Contact, Amsterdam – Brussel.

Kahn R. (2011) De appel en de boom. Waarom ben je wie je bent? Is dat aanleg of opvoeding? Uitgeverij Balans, Amsterdam
