# Supplementary material for: Effect of play-based family-centered psychomotor/psychosocial stimulation on the development of severely acutely malnourished children under six in a low-income setting: a randomized controlled trial
Source: BMC Pediatr. 2019 Sep 14;19:336. doi: 10.1186/s12887-019-1696-z (PMC6744679; doi:10.1186/s12887-019-1696-z)
Supplement: Supplementary file 3 — Figure S1 Developmental outcomes of the control and intervention SAM children during 6 months of follow-up (DOCX 36 kb) [file 12887_2019_1696_MOESM3_ESM.docx]

|  |  |  |
| --- | --- | --- |
| (a) |  | (b) |
|  |  |  |
| (c) |  | (d) |
|  |  |  |
| (e) |  |  |
| **Figure S2**  Developmental outcomes of the control and intervention SAM children during six months of follow-up | | |
