# Supplementary material for: Effect of play-based family-centered psychomotor/psychosocial stimulation on the development of severely acutely malnourished children under six in a low-income setting: a randomized controlled trial
Source: BMC Pediatr. 2019 Sep 14;19:336. doi: 10.1186/s12887-019-1696-z (PMC6744679; doi:10.1186/s12887-019-1696-z)
Supplement: Supplementary file 4 — Table S1 Baseline and end-line developmental performance and WAZ scores of SAM children a followed up for 6 months after discharge from hospital compared with healthy children b (DOCX 17 kb) [file 12887_2019_1696_MOESM4_ESM.docx]

| **Table S4**. Age wise comparison **^a^** of the control (n=98) and the intervention (n=113) SAM children who completed the study | | | | | | | | | | | | | |
| --- | --- | --- | --- | --- | --- | --- | --- | --- | --- | --- | --- | --- | --- |
|  | Number of SAM children allocated to control and intervention by age category | | | | | | | | | | | | |
|  | < 12 mo. | | 12-24 mo. | | 24-36 mo. | | 36-48 mo. | | 48-60 mo. | | 60-65 mo. | |  |
| Follow-up phase | Con | Int | Con | Int | Con | Int | Con | Int | Con | Int | Con | Int | $chi2$ |
| Baseline | 21 | 15 | 34 | 39 | 16 | 29 | 14 | 18 | 8 | 11 | 5 | 1 | 7.7  p=0.183 |
| Discharge | 19 | 14 | 33 | 38 | 16 | 39 | 10 | 20 | 6 | 11 | 14 | 1 | 19.97**^±^**  p=0.001 |
| End-line | - | - | 39 | 29 | 24 | 38 | 14 | 27 | 14 | 13 | 7 | 6 | 7.84  p=0.096 |
| **^a^** Comparison using Chi-square test of association  **^±^** When age categories >= 48 mo. are excluded there is no significant association ($Chi2$ = 5.3, p=0.153)  Con, control; Int, intervention | | | | | | | | | | | | | |
