# Supplementary material for: Effect of play-based family-centered psychomotor/psychosocial stimulation on the development of severely acutely malnourished children under six in a low-income setting: a randomized controlled trial
Source: BMC Pediatr. 2019 Sep 14;19:336. doi: 10.1186/s12887-019-1696-z (PMC6744679; doi:10.1186/s12887-019-1696-z)
Supplement: Supplementary file 5 — Table S2 The overtime change a in developmental b performance, linear growth c and nutritional status d of SAM children during a follow-up in hospital as an in-patient and at home after discharge for a period of 6 months (DOCX 20 kb) [file 12887_2019_1696_MOESM5_ESM.docx]

| **S2 Table**. The overtime change **^a^** in developmental **^b^** performance, linear growth **^c^** and nutritional status **^d^** of SAM children during a follow-up in hospital as an in-patient and at home after discharge for a period of six months | | | | | | | |
| --- | --- | --- | --- | --- | --- | --- | --- |
|  |  | Increase during hospital follow-up, mean [95%CI] | | | Increase during home follow-up, mean [95%CI] | | |
| Outcome measures | Baseline mean  [95% CI] | control | intervention | Diff, mean [95%CI]  p-value | Control | intervention | Diff, mean [95%CI]  p-value |
| FM | 15.5 [14.9, 16.1 | 0.73 [0.3, 1.2]  0.9^±^ [0.6, 1.2] | 1.04 [0.6, 1.5]  0.9^±^ [0.6, 1.2] | 0.3 [-0.3, 0.9] p=0.316  - | 2.5 [1.8, 3.1]  2.5^±^ [1.9, 3.1] | 3.6 [3.2, 4.0]  3.6^±^ [3.2, 40] | 1.2 [0.5, 1.8] p= 0.001  1.09^±^ [0.3, 0.5] p= 0.001 |
| GM | 16.3 [15.7, 16.8] | 0.36 [0.02, 0.7] 0.4^±^ [0.1, 0.7] | 1.3 [1.0, 1.7]  1.3^±^ [0.9, 1.6] | 0.97 [0.5, 1.5] p<0.001  0.88^±^ [0.4, 1.4] p<001 | 4.3 [3.6, 4.9]  - | 4.7 [4.0, 5.3]  - | 0.4 [-0.5, 1.3] p= 0.377  - |
| LA | 16.9 [16.1, 17.6] | 0.5 [0.1, 1.0] | 0.5 [0.01, 1.0] | 0.01 [-0.7, 0.7] p= 0.989 | 3.8 [3.0, 4.5] | 4.3 [3.6, 5.0] | 0.5 [-0.5, 1.5] p=0.332 |
| PS | 14.1 [14.7, 43] | 0.7 [0.3, 1.0] | 0.6 [0.3, 0.9] | -0.06[-0.6, 0.4] p= 0.808 | 3.4 [2.6, 3.8] | 3.3 [2.9, 3.8] | 0.06 [-0.7, 0.8] p=0.867 |
| SE | 67 [40, 70.3] | -12.8 [-17, -8.5] | -11.1 [-16,6.4] | 1.7 [-4.3, 7.7] p= 0.585 | -14.6[-20, -9] | -17.2[-22, 12.7] | -2.7[-9.2, 3.9] p=0.425 |
| HAZ | -3.8 [-4, -3.6] | -0.05 [-0.2, 0.1] | -0.1 [-0.3, 0.1] | -0.05 [-0.3, 0.2] p=0.623 | 0.2 [-0.1, 0.4] | -0.15[-0.03, 0.3] | -0.02[-0.3, 0.3] p=0.891 |
| MUACZ | -3.3 [-3.5, -3.1] | 0.3 [0.1, 0.4] | 0.4 [0.3, 0.5] | 0.2 [-0.1, 0.3] p=0.140 | 2.1 [1.8, 2.4] | 2.1 [1.8, 2.4] | -0.01[-0.4, 0.3] p=0.927 |
| WAZ | -3.7[-3.9, -3.6] | 0.2[0.03, 0.3] | 0.3[0.2, 0.4] | 0.1[-0.1, 0.3] p=0.270 | 1.3[1.0, 1.5] | 1.1[0.7, 1.4] | -0.2 [-0.7, 0.2] p=0.365 |
| WHZ or BAZ | -2.4 [-2.6, -2.2] | 0.5[0.3, 0.8] | 0.5[0.4, 0.7] | 0.04[-0.3, 0.3] p=0.810 | 2.0[1.7, 2.3] | 1.8[1.6, 2.0] | -0.2 [-0.5, 0.2] p=0.347 |
| **^a^** parameter estimated from the GEE model which assumed same intercept for both the control and the intervention children at baseline measurement.  **^b^** developmental outcomes measured on performances in FM, GM, LA, PS and SE; **^c^** linear growth indicated as HAZ score; **^d^** nutritional outcome indicated in MUACZ, WAZ and WHZ or BAZ scores  BAZ, body-mass-index-for-age z score; FM, fine motor; GEE, Generalized Estimating Equations; GM, gross motor; HAZ, height/length-for-age z score; LA, language; MUACZ, mid-upper-arm circumference-for-age z score z score; PS, personal social; SE, social-emotional; WAZ, weight-for-age z score; WHZ, weight-for-height/length z score.  ^±^ Estimates calculated from only significant parameters in the GEE model | | | | | | | |
