# Supplementary material for: Effect of play-based family-centered psychomotor/psychosocial stimulation on the development of severely acutely malnourished children under six in a low-income setting: a randomized controlled trial
Source: BMC Pediatr. 2019 Sep 14;19:336. doi: 10.1186/s12887-019-1696-z (PMC6744679; doi:10.1186/s12887-019-1696-z)
Supplement: Supplementary file 6 — Table S3 Regression coefficients [95% CI] from the GEE models a separately examining some selected factors modifying the effect of the intervention on development, linear growth and nutritional outcomes (DOCX 18 kb) [file 12887_2019_1696_MOESM6_ESM.docx]

| **S3Table.** Regression coefficients [ 95% CI] from the GEE models ^a^ separately examining some selected factors modifying the effect of the intervention on development, linear growth and nutritional outcomes | | | |
| --- | --- | --- | --- |
|  | **Selected explanatory variables** | | |
| Outcomes | Duration of follow up (days in study) | Number of stimulation sessions in hospital **^b^** | Whether or not the intervention SAM child was sick after discharge from hospital |
| FM | 0.48 [0.41, 0.54] p<0.001 | 0.14 [0.01, 0.27] p= 0.040 | -0.4 [-1.2, 0.4] p= 0.335  -0.4 [-1.2, 0.4] p= 0.289 |
| GM | 0.62 [0.52, 0.71] p<0.001 | 0.18 [0.02, 0.33] p= 0.026 |  |
| LA | 0.59 [0.49, 0.69] p<0.001 | 0.23 [0.03, 0.44] p= 0.024 | -0.01 [-0.8, 0.7] p= 0.976 |
| PS | 0.47 [0.40, 0.54] p<0.001 | 0.17 [0.003, 0.33] p= 0.046 | -0.3 [-1.0, 0.4] p= 0.417 |
| SE | -1.9 [-2.6, -1.09] p<0.001 | 0.22 [-0.5, 1.0] p= 0.550 | -4.3 [-11.0, 2.3] p=0.204 |
| HAZ | 0.03 [0.003, 0.05] p=0.027 | -0.004 [-0.05, 0.04] p= 0.862 | -0.2 [-0.4, -0.05] p= 0.010 |
| MUACZ | 0.32 [0.27, 0.36] p<0.001 | -0.02 [-0.07, 0.02] p= 0.369 | -0.3 [-0.5, 0.06] p= 0.128 |
| WAZ | 0.13 [0.07, 0.20] p<0.001 | -0.004 [-0.04, 0.04] p= 0.836 | -0.2 [-0.4, 0.06] p= 0.128 |
| WHZ or BAZ | 0.24 [0.20, 0.28] p<0.001 | -0.01[-0.06, 0.03] p= 0.500 | 0.1 [-0.2, 0.4] p= 0.496 |
| **^a^** All the covariates were entered separately into the GEE model for each outcome.  **^b^** ‘Stimulation sessions’ refers to total number of play sessions in play room and playground the intervention SAM child received before discharge from hospital; **^c^** reference is only ‘once a day’  BAZ, body-mass-index-for-age-z score; FM, fine motor; GEE, Generalized Estimating Equations; GM, gross motor; HAZ, height/length-for-age-z score; LA, language; UACZ, mid-upper-arm-circumference-for-age z-score; PS, personal social; SE, social-emotional; WAZ, weight-forage-z score; WHZ, weight-for-height/length-z-score; | | | |
