# Supplementary material for: Effect of play-based family-centered psychomotor/psychosocial stimulation on the development of severely acutely malnourished children under six in a low-income setting: a randomized controlled trial
Source: BMC Pediatr. 2019 Sep 14;19:336. doi: 10.1186/s12887-019-1696-z (PMC6744679; doi:10.1186/s12887-019-1696-z)
Supplement: Supplementary file 7 — Table S4 Baseline and end-line developmental performance and WAZ scores of SAM children a followed up for 6 months after discharge from hospital compared with healthy children b (DOCX 18 kb) [file 12887_2019_1696_MOESM7_ESM.docx]

| S4 Table. Baseline and end-line developmental performance and WAZ scores of SAM children ^a^ followed up for six months after discharge from hospital compared with healthy children ^b^ | | | | |
| --- | --- | --- | --- | --- |
|  | **Baseline: before start of intervention** | | **End-line: more than six months after start of intervention** | |
|  | Control  Vs  Healthy | Intervention  Vs  Healthy | Control  Vs  Healthy | Intervention  Vs  Healthy |
|  | Mean difference **^c^**  (95% CI) | Mean difference **^c^**  (95% CI) | Mean difference **^d^** (95% CI) | Mean difference **^d^** (95% CI) |
| Fine Motor **^e^** | -3.5***  (-3.9, -3) | -3.2***  (-3.7,-2.9) | -3***  (-3.4,-2.6) | -2***  (-2.4, -1.6) |
| Gross Motor **^e^** | -5.76***  (-6.3, -5.2) | -5.79***  (-6.3,-5.3) | -4***  (-4.7, -3.6) | -4***  (-4.5, -3.5) |
| Language **^e^** | -5.1***  (-5.7, -4.5) | -5.4***  (-6, -4.8) | -4.6***  (-5.3, -4) | -4.6***  (-5.3. -4) |
| Personal Social **^e^** | -2.9***  (-3.6, -2.3) | -2.4***  (-3, -1.8) | -1.9***  (-2.4, -1.5) | -1.5***  (-1.9, -1.1) |
| Social-Emotional **^f^** | 18.3***  (13.1, 23.5) | 17.9***  (13.1,22.8) | -0.7  (-4.53, 6) | -1.9  (-6.7, 3) |
| WAZ **^f^** | -3.6***  (-4.3, -2.9) | -3.3***  (-3.9,-2.6) | -2.4***  (-2.6, -2.1) | -2. 4***  (-2.7,-2.2) |
| **^a^** SAM control (n=98); intervention (n=113)  **^b^** Healthy (n=1528 for baseline, and n=1318 for end-line)  **^c^** ANCOVA controlling for age  **^d^** ANCOVA controlling for **^e^** age; **^f^** age and sex.  Mean difference significant at *p<0.05; **p<0.01; ***p<0.001  ANCOVA= analysis of covariance; SD, Standard deviation; SAM= Severe acute malnourished; SE= social-emotional; WAZ= weight-for-age z-score | | | | |
